# Development of a Core Set of Outcomes for Randomized Controlled Trials with Multiple Outcomes – Example of Pulp Treatments of Primary Teeth for Extensive Decay in Children

**DOI:** 10.1371/journal.pone.0051908

**Published:** 2013-01-03

**Authors:** Violaine Smaïl-Faugeron, Hélène Fron Chabouis, Pierre Durieux, Jean-Pierre Attal, Michèle Muller-Bolla, Frédéric Courson

**Affiliations:** 1 AP-HP, Hôpital Bretonneau, Service d'Odontologie, Paris, France; 2 Université Paris Descartes – Sorbonne Paris Cité, Faculté de Chirurgie Dentaire, Unité de Recherches Biomatériaux Innovants et Interface EA4462, Montrouge, France; 3 AP-HP, Hôpital Charles Foix, Service d'Odontologie, Ivry-sur-Seine, France; 4 Université Paris 13 – Sorbonne Paris Cité, Ecole doctorale Galilée, Villetaneuse, France; 5 Institut National de la Santé et de la Recherche Médicale, UMR S 872, Equipe 20, Centre de Recherche des Cordeliers, Paris, France; 6 Université Paris Descartes - Sorbonne Paris Cité, Faculté de Médecine, Paris, France; 7 AP-HP, Hôpital Européen Georges Pompidou, Département d'Informatique Hospitalière, Paris, France; 8 Université de Nice-Sophia Antipolis, Service d'Odontologie, Faculté de Chirurgie Dentaire, Nice, France; University of York, United Kingdom

## Abstract

**Objectives:**

Evidence-based comparisons of interventions can be challenging because of the diversity of outcomes in randomized controlled trials (RCTs). We aimed to describe outcomes in RCTs assessing pulp treatments for primary teeth and to develop a core set of component outcomes to be part of composite outcome defining the failure of a pulp treatment.

**Methods:**

We systematically reviewed articles of RCTs comparing pulp treatments for primary molars published up to February 2012. We abstracted all outcomes assessed in each trial, then used a small-group consensus process to group similar outcomes, which were reduced to a composite outcome of failure of a pulp treatment by a 3-round Delphi process involving expert authors and dentists.

**Results:**

We included 47 reports of RCTs in the review, for 83 reported outcomes (median 11 outcomes per RCT). These outcomes were grouped into 24 overarching outcome categories. We contacted 210 experts for the Delphi process and 25% to 30% participated. The process identified the following 5 component outcomes as part of a composite outcome of failure of a pulp treatment: soft-tissue pathology, pain, pathologic mobility, pathologic radiolucency and pathologic root resorption.

**Conclusions:**

RCTs of pulp treatments for primary teeth investigate diverse outcomes. Our consensus process, involving clinicians but no patient, allowed for compiling a core set of component outcomes to define the composite outcome failure of a pulp treatment for primary teeth.

## Introduction

In children, extensive tooth decay is the most common disease of primary teeth; 42% of children aged 2 to 11 have dental caries in their primary teeth, with an average of 1.6 decayed teeth per child [1]. Depending on the severity of the disease, 3 pulp treatments are available: direct pulp capping, pulpotomy and pulpectomy [2]. A large number of biomaterials are available to fill the cavity after treatment. The efficacy of these interventions, combining 1 pulp treatment and 1 biomaterial, needs assessment and many randomized controlled trials (RCTs) have been conducted to compare interventions.

The efficacy of pulp treatments may be measured in various ways, commonly by both clinical and radiological dimensions. A success or failure composite outcome is often used but is defined by various component outcomes across trials. Consensus is lacking regarding the most relevant outcomes, especially for the definition of success or failure. Most investigators use their own criteria. Moreover, all these outcomes are frequently assessed at different times within and across trials.

Although multiple endpoints may be a scientific requirement, the diversity in reported outcomes and the lack of broadly accepted criteria for success hampers secondary research [3], [4]. In fact, the multiplicity of outcome selection and measurement in pulp treatment trials may hinder or affect the findings and synthesis of results across trials with meta-analyses [5], [6], [7]. Moreover, without a consensual and validated set of outcomes, clinical researchers may favor outcomes that enhance trial feasibility or results rather than clinician- or patient-important outcomes; as well, assessing the extent of selective outcome reporting may be difficult [8], [9].

Our first aim was to describe exhaustively the range of outcomes used in RCTs assessing the effectiveness of pulp treatments in primary teeth. Particularly, we aimed to identify in each trial all component outcomes that were part of a composite outcome defining the success or failure of a pulp treatment. The second objective was to assess the similarity of reported outcomes and group the outcomes that could be lumped together in a meta-analysis. Our third objective was to develop a core set of preferred component outcomes as part of a composite outcome defining failure of a pulp treatment.

## Methods

First, we systematically reviewed RCTs assessing the effectiveness of pulp treatment techniques in primary molars to identify the diversity of outcomes in general and particularly component outcomes that were part of a composite outcome defining the success or failure of a pulp treatment. Second, we used a small-group consensus process to assess the similarity of outcomes and component outcomes. Third, we used a Delphi process to identify a core set of component outcomes as part of a composite outcome defining failure of a pulp treatment.

### Systematic review of outcomes

**Search and selection of trials. Eligible studies were RCTs comparing different pulp treatments with each other (direct pulp capping, pulpotomy or pulpectomy) in children with extensive decay in primary molar teeth.**

To identify trials, we searched bibliographical databases (Cochrane Oral Health Group's Trials Register CENTRAL; MEDLINE; EMBASE; Science Citation Index Expanded; Social Science Citation Index; Index to Scientific and Technical Proceedings; System for Information on Grey Literature in Europe). Search equations for each database were built by the Trial Search Coordinator of the Cochrane Oral Health Group. The equations combined free text words and controlled vocabulary pertaining to the condition and interventions (see Supporting Information, Text S1 [10]). The last search for articles was conducted in February 2012, with no restriction on date or language. We also screened the reference lists of included reports and searched ClinicalTrials.gov for the protocols of included studies and to identify ongoing trials.

Two authors (V. S. and H. F.) independently and in duplicate screened the titles and abstracts of records retrieved by the search, then screened the selected full-text reports. Any disagreements were resolved by discussion. These 2 reviewers independently collected data on publication year, number of arms in the trial, treatments compared, duration of follow-up, number of patients enrolled and number of treated teeth. Finally, the risk of bias within each RCT was assessed by the Cochrane Collaboration Risk of Bias tool, which includes the following items: methods for sequence generation and maintaining allocation concealment, blinding, incomplete outcome data, and selective outcome reporting [11], [12]. Each domain was rated as having low, high, or unclear risk of bias. Then, each RCT was assigned an overall risk of bias score: low risk (low for all key domains), high risk (high for 1 key or more domains), or unclear risk (unclear for 1 key or more domains).

#### Outcomes

The aim of a pulp therapy is to retain the primary tooth, maintain its supporting tissues and preserve the space required for the eruption of the permanent tooth, without compromising the development of the succedaneous permanent tooth germ. The efficacy of a pulp treatment is commonly assessed by both clinical and radiological dimensions. Moreover, the assessment of efficacy is commonly based on a success or failure composite outcome, defined by various component outcomes and/or individual outcomes.

We identified all outcomes assessed in each included trial. An outcome designates individual components of the success or failure composite outcome or other individual outcomes. We abstracted the precise definitions of clinical, radiological and overall success or failure (ie, which component outcomes were part of the composite success or failure outcomes). Each individual outcome or component outcome was detailed in terms of its specific name or label, the time(s) when it was assessed and whether it was the “primary outcome” of the study (explicitly described as such or used in a power calculation to determine study design). For each report of RCT, we also assessed whether each outcome was defined in the Methods section and whether numerical data for each outcome (ie, number of patients with the outcome in experimental and control arms) were reported in the Results section. All outcomes and component outcomes reported for a given trial were recorded on a single data extraction form. We created a comprehensive inventory of outcomes and component outcomes from all data extraction forms.

### Assessment of the similarity of outcomes

Because we expected a large diversity in reported outcomes, we grouped similar outcomes into overarching outcome categories by a small-group consensus process. The group of experts consisted of 6 doctors in dental surgery specialized in pediatric dentistry, including 3 clinical research investigators. First, the group identified outcomes that were identical despite different terms used across trials. Second, different but close outcomes (ie, outcomes that could be compared across studies or combined in a meta-analysis) were grouped together into outcome domains. Finally, the group, with consensus, determined several outcome categories and produced a reduced-outcome inventory.

### Assessment of the most relevant component outcomes to be part of the composite outcome defining failure of a pulp treatment

We used a 3-round electronic Delphi survey design to achieve consensus on a core set of component outcomes for clinical trials evaluating pulp treatments for primary teeth. The Delphi method, a well-recognized method to elicit expert consensus, is based on an iterative process with anonymous consultation with experts, with controlled feedback and quantified analysis of the group's responses [13], [14]. We chose the important methodological features for our Delphi process (composition of the group, anonymity, assessment of the importance of outcomes, feedback of results to participants, how consensus was reached, attrition) using the results of a systematic review of studies that used the Delphi technique to determine which outcomes to measure in clinical trials (Supplementary Information, Table S1) [15]. We used the checklist recommended in this review to report our Delphi process.

We used SurveyMonkey for the Delphi process [16]. Each round had a response closing date of 21 days after the date of invitation. An e-mail reminder was sent to contacts on days 7 and 14. Non-responders were invited to participate in subsequent rounds. The setting was multinational. We contacted authors of the trials included in our systematic review and dentists from the French College of Pediatric Dentistry Teachers (Collège des Enseignants en Odontologie Pédiatrique, COLEP). To support the content validity of the process, we contacted experts who had several years of experience in the diagnosis and treatment of extensive decay in primary teeth, had published articles on the topic and/or had been study investigators. We contacted members of COLEP by the president of COLEP sending our letter of invitation to participate, and we invited international authors to take part in the Delphi process by the Deputy Managing Editor of the Cochrane Oral Health Group (COHG) sending our letter of invitation to participate on behalf of the COHG.

#### Round 1

We presented the reduced outcome inventory to Delphi participants and asked them to rate the importance of each (component) outcome on a 5-point Likert-type scale: 1, no importance; 2, some importance; 3, moderate importance; 4, very important; and 5, extremely important. We also invited the participants to choose the minimum follow-up duration.

#### Round 2

The results from the first round were relayed back to participants. Only outcomes or component outcomes rated very or extremely important by at least 50% of participants were carried forward to round 2. Round 2 presented the overall group's percentage rating for each outcome and component outcome retained from round 1. First, participants were asked to define failure of a pulp treatment by clinical, radiological, or both component outcomes. In addition, participants were asked to choose the component outcomes (among outcomes and component outcomes carried forward to round 2) that should be part of the composite outcome defining failure of a pulp treatment. Finally, the results from round 1 for the minimum follow-up duration were relayed back to participants, who were asked to determine the optimal follow-up duration.

#### Round 3

Round 3 sought to obtain broader consensus on the core set of outcomes. The results from round 2 were relayed back to participants. Only component outcomes chosen by at least 50% of participants were carried forward to round 3. The overall group percentage rating was presented for each component outcome retained in round 2. The participants were asked to re-select the component outcomes (among those carried forward to round 3) that should be part of the composite outcome defining failure of a pulp treatment with knowledge of the group's previous ratings. In addition, they were asked to re-determine the optimal follow-up duration. Component outcome measures chosen by 70% or more of participants were retained in round 3.

### Data analysis

Data are summarized as number (percentage) or median (25–75% percentile). To visualize the variety of outcomes used, we drew an adjacency matrix showing the outcome domains reported by each trial, with outcomes reported in the trial reports listed in rows and the different trials listed in columns [17]. We also drew a network of outcomes [18]. Each node in the figure represented each possible outcome domain (in the reduced inventory from the small-group consensus process). The size of nodes was proportional to the number of trials that assessed the corresponding outcome. A vertex linked 2 nodes when the 2 respective outcome domains had been assessed together in a same trial. The width of vertices was proportional to the number of trials that assessed the 2 corresponding outcome domains. Finally, we performed the same analyses but for only component outcomes as defined in the Methods section of included reports. Analyses involved use of R v2.12.0 (R Development Core Team, Vienna, Austria).

## Results

### Systematic review of outcomes

#### Description of trials

**Figure 1:**
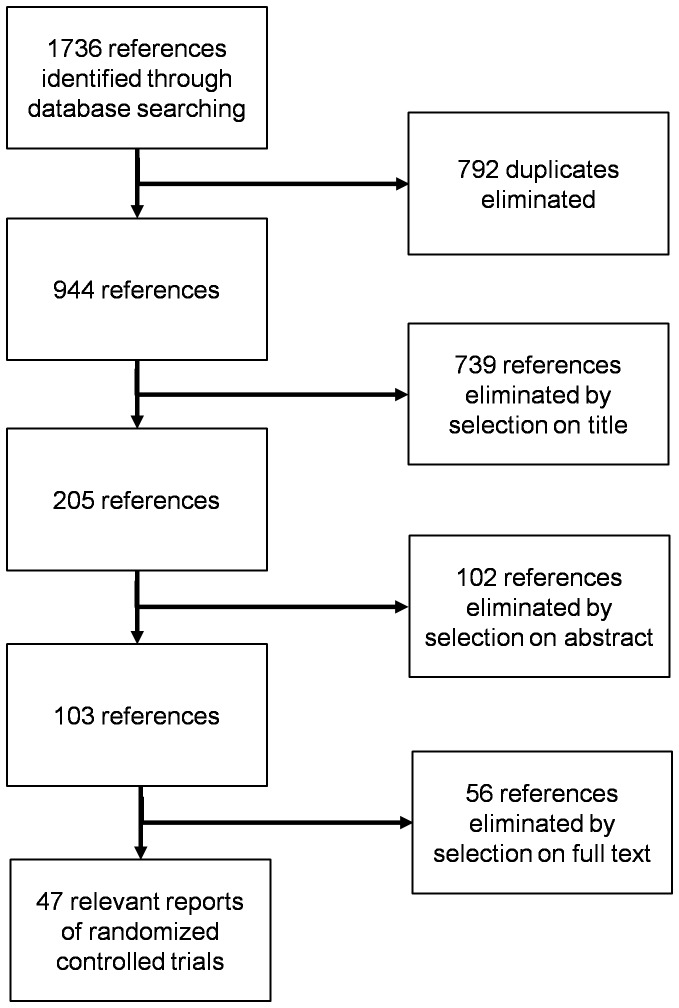
Flow chart of articles in the systematic review.

The search yielded 1,736 potentially eligible articles. We included 47 reports of RCTs in the review. The flow chart of article selection is in Figure 1. Included and excluded reports of studies are in Supporting Information, Text S2. We did not find the protocol for any included trial at ClinicalTrials.gov. We identified 1 ongoing trial, but the outcomes definitions were not detailed [19].

The characteristics of included studies are in Table 1. Details for each included RCT are in Supplementary Information, Table S2. The year of publication was between 2005 and 2012 for 72% of reports, with 3 in 2011 and 1 in 2012. In total, 70% of reports described 2-arm studies. The 47 reports described 44 different interventions combining a treatment (pulpotomy, pulpectomy or direct pulp capping) and a biomaterial (among 40 different biomaterials), for 61 different comparisons between interventions. In total, 70% of reports described the comparison of different biomaterials for pulpotomy. The duration of follow-up was fixed in 81% of reports, at 6 and 12 months in more than 55%. The median number of enrolled patients per RCT was 42 [quartile 1–3 [Q1–Q3] 27–71, min–max 15–152]. The median number of treated teeth per RCT was 68 [Q1–Q3 50–100, min–max 30–291]. The overall risk of bias was low for only 1 (2%) RCT. For 27 reports (57%), the risk of bias was unclear, frequently due to lack of information about allocation concealment and blinding of participants and staff. In total, 18 reports (38%) described attrition bias, due to amount, nature or handling of incomplete outcome data. Clinical outcomes' assessment was not blinded in 5 reports (11%), and the sequence generation was inadequate in 1. Consequently, the overall risk of bias was high in 19 reports (40%).

**Table 1:** Characteristics of included studies.

Characteristics of RCTs	Number of RCTs (%)n = 47
Publication year	
<2000	5 (11%)
2000–2004	8 (17%)
2005–2009	23 (49%)
2010–2012	11 (23%)
Risk of bias	
Low risk	1 (2%)
High risk	19 (40%)
Unclear	27 (57%)
Number of randomly allocated teeth	
Median [25–75% percentile], [min–max]	68 [50–100], [30–291]
Number of arms	
2	33 (70%)
3	8 (17%)
4	5 (11%)
5	1 (2%)
Treatments compared	
Pulpotomy vs pulpectomy*	2 (4%)
Pulpotomy vs pulpotomy	33 (70%)
Pulpectomy vs pulpectomy	8 (17%)
Direct vs indirect pulp capping	4 (9%)
Follow-up time	
Fixed duration of follow-up†	38 (81%)
3 months	15 (32%)
6 months	33 (70%)
9 months	6 (13%)
12 months	26 (55%)
18 months	11 (23%)
24 months	14 (30%)
36 months	1 (2%)
Variable duration of follow-up	9 (19%)

RCT, randomized controlled trial;

* biomaterials were always different;

† numbers do not add to 100% because several time points could be reported in each trial.

#### Description of outcomes

We found a total of 83 reported outcomes, with a median number of outcomes per RCT of 11 (Table 2). An adjacency matrix of outcomes illustrates the diversity of reported outcomes (Supplementary Information, Figure S1). In all, 78 of the outcomes pertained to primary teeth, for 39 clinical and 39 radiological outcomes. The remaining 5 concerned permanent teeth (4 clinical outcomes and 1 radiological outcome) and were reported for 9 trials. The most frequently reported clinical outcomes were pathological mobility (37 reports) and pain symptom (35 reports) and the most frequently reported radiological outcomes were furcal/bifurcation radiolucency (36 reports) and internal root resorption (35 reports) (Supplementary Information, Table S3). Twenty-eight outcomes were defined in only 1 report each.

**Table 2:** Reporting of outcomes in selected RCTs.

	Total number	Number of RCTs	Number per RCT*	Min–max
All outcomes	83	47	11 [9–13]	1–22
Clinical outcomes concerning primary teeth	39	46	6 [5–7]	0–11
Radiological outcomes concerning primary teeth	39	47	5 [4–7]	1–10

* median [25–75% percentile].

No report defined the primary outcome (no outcome was explicitly described as such or used in a power calculation). The clinical, radiological or overall success or failure of a pulp treatment was defined in 40 reports (85%) and overall success or failure was defined in 24 (51%). Among 40 reports, 39 (98%) defined both clinical and radiological success or failure, of which 16 (41%) did not define overall success or failure. The median number of component outcomes defining success or failure per RCT was 9 [Q1–Q3 5–10, min–max 1–20]. Of note, the definition of success differed from that of failure for 2 RCTs (9 and 13 component outcomes differed).

There were important differences between outcomes defined in the Methods and those reported in the “Results sections. In all 47 reports, at least 1 outcome defined in the Methods section was not reported in the Results section or vice versa (Table 3 and Supplementary Information, Table S4).

**Table 3:** Discrepancies in the reporting of outcomes between Methods and Results sections of reports of RCTs.

	Number of RCTs (n = 47)	Number per RCT*	Min–max
Outcomes defined in Methods and reported in Results	44 (94%)	4 [2–6]	0–11
Outcomes defined in Methods but not reported in Results	41 (87%)	5 [3–9]	0–14
Outcomes not defined in Methods but reported in Results	36 (74%)	1 [1–2]	0–10
Component outcomes defined in Methods and reported in Results	36 (74%)	3 [1–5]	0–11
Component outcomes defined in Methods but not reported in Results	35 (74%)	4 [0–6]	0–14

* median [25–75% percentile].

### Assessment of the similarity of outcomes

The inventory of outcomes presented to the group of experts included 80 outcomes. From the 83 unique reported outcomes, we added 3 outcomes retained in the Cochrane review by Nadin et al. [10] but not assessed in the included RCTs. We dropped 2 clinical and 4 radiographic composite scores (each reported in a single RCT) that combined various component or individual outcomes already identified in the list of outcomes. We did not consider primary tooth survival, reported in 2 articles, because it cannot be considered a component outcome of a treatment's success or failure, although it is a patient-important outcome.

The experts reduced the 80 outcomes to 24 outcome categories (Table 4): 11 clinical and 8 radiological categories pertained to primary teeth and 5 to succedaneous permanent teeth. Fourteen outcomes were not grouped. For the 10 other outcome domains, the median number of outcomes per outcome domain was 5 [min–max 2–19]. Among the 3 outcomes retained in the Cochrane review by Nadin et al. [10] but not assessed in the included RCTs, 2 were not grouped with other outcomes and formed 2 distinct outcome domains, and 1 was grouped with another one.

**Table 4:** The 24 overarching outcome categories reduced from 83 outcomes assessed for similarity by small-group consensus.

Overarching outcome categories	Number of similar outcomes	Similar outcomes
*Clinical outcomes concerning primary teeth*		
1. Adjacent tissues inflammation	6	Severe gingival inflammation/Changes in the mucous membrane in the surrounding area – appearance of surrounding tissue/Redness around the tooth-crown/Bleeding around the tooth or crown/Erythema/Inflammation in the adjacent tissues
2. Defective restoration (clinically)	4	Partially or completely lost fillings/Perforated or lost final restoration/Defective restoration/Restoration intact
3. Pain	10	Pain symptom/Spontaneous pain/Thermal sensitivity/Pain initiated by stimuli/Sensitivity to pressure/Tenderness to percussion/Chewing sensitivity/Sensitivity to sour/Sensitivity to sweet/Pain on palpation – palpation sensitivity
4. Secondary caries at the margin (clinically)	2	Marginal integrity/Secondary caries at the margin
5. Soft tissue pathology	10	Swelling/Edema/Soft tissue pathology-swelling/Extraoral swelling/Intraoral swelling/Infection in the adjacent tissues/Fistulation – fistula/Parulis/Abscess/Sinus tract
6. Periodontal pocket formation	1	Periodontal pocket formation (exudate or no exudate)
7. Dental anxiety/phobia*	1	
8. Pathologic mobility	1	
9. Premature tooth loss	1	
10. Signs of exfoliation	1	
11. Smell	1	
*Radiologic outcomes concerning primary teeth*		
12. Pathologic radiolucency	19	Pathologic radiolucency/Periapical radiolucency/Lateral radiolucency/Apical radiolucency/Involvement of the apical area/Radicular radiolucency/Periradicular radiolucency/Furcal –bifurcation radiolucency/Intra-radicular radiolucency/Bone radiolucency/Furcation involvement/Periapical bone destruction/Interradicular bone destruction/Abnormal inter-radicular trabeculation – variation in radiodensity/Periodontal ligament space widening/Integrity of lamina dura/Loss of trabecular bone/Abnormalities in the structure of trabecular bone/Bone regeneration
13. Pathologic root resorption	8	Pathologic root resorption/Root resorption in relation to contralateral tooth/Internal root resorption/Internal root resorption-perforated form/Internal dentine resorption/External root resorption/Replacement resorption/Root resorption in relation to contralateral tooth with criteria established by Wright
14. Pulp canal obliteration	3	Pulp canal obliteration – intracanal calcifications – canal calcifications/Calcific metamorphosis/Calcific degeneration of the pulp
15. Defective restoration (radiographically)	1	
16. Dentine bridge formation	1	
17. Filling material anomaly	1	Excess filling material and its resorption
18. Physiological resorption	1	
19. Secondary caries (radiographically)	1	Recurrent caries
*Outcomes concerning succedaneous permanent teeth*		
20. Succedaneous tooth structural anomaly	2	Dental anomalies (e.g. hypoplasia of premolars)*/Succedaneous tooth structural anomaly (e.g. hypoplasia)
21. Unerupted succedaneous tooth anomaly (radiographically)	2	Damage to succedaneous follicle/Deviated eruption of succedaneous teeth – position and eruption pathway of the permanent successor tooth
22. Erupting succedaneous tooth mobility	1	
23. Signs/symptoms of erupting succedaneous tooth	1	
24. Succedaneous tooth position anomaly*	1	Problems in the developing occlusion (e.g. loss of space, mesial drift of first permanent molars, etc.)

“…”**–**“…”: identical outcomes; “…” **/** ”…”: different but close outcomes.

* outcomes retained in the Cochrane review by Nadin et al. [Nadin G, Goel BR, Yeung CA, Glenny AM (2003) Pulp treatment for extensive decay in primary teeth. Cochrane Database Syst Rev: CD003220.].

**Figure 2:**
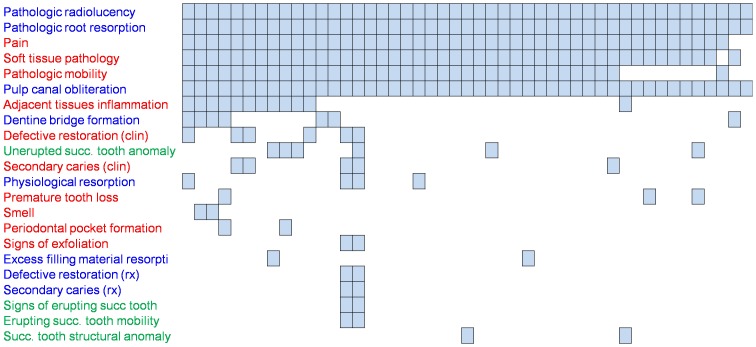
Adjacency matrix of 24 outcome domains for 47 articles of randomized controlled trials (RCTs). Two outcome domains are not represented because they were retained from the Cochrane review by Nadin et al. [10] but not assessed in the included RCTs.

We re-assessed how these 24 outcome domains were reported in RCTs. The adjacency matrix of outcomes revealed substantial diversity among reported outcomes, although 6 outcome domains were frequently reported (Figure 2). The network of outcomes showed that these outcomes were most frequently reported together (Supplementary Information, Figure S2).

### Assessment of the most relevant component outcomes to be part of a composite outcome defining the failure of a pulp treatment

**Figure 3:**
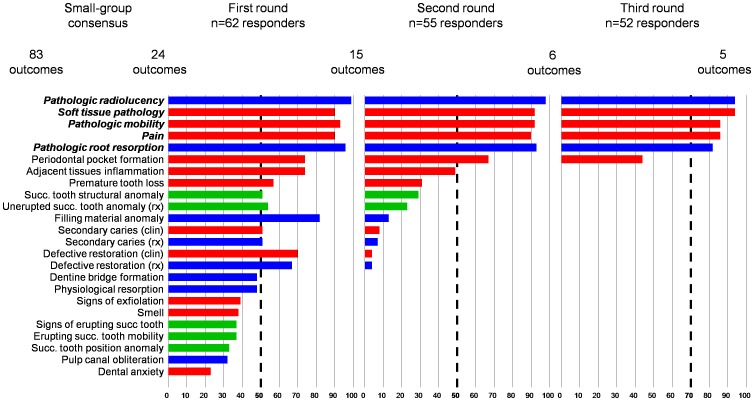
Outcomes flow chart and results of the Delphi process. Succ, succedaneous. Clinical outcomes are in red, radiological outcomes in blue, outcomes pertaining to permanent teeth in green.

We invited 210 people, including 135 international authors and 75 French dentists, to participate in the Delphi process. Figure 3 shows the percentage of component outcomes the respondents considered important, the outcomes selected in each round and the resulting core set of component outcome measures.

In total, 62 respondents (30%) completed round 1. At least 50% of the participants rated 15 of the 24 outcomes categories as very or extremely important. Moreover, 42% of participants considered 24 months as the minimum follow-up duration (29% chose 12 months).

In total, 55 participants (26%) completed round 2. In all, 96% considered that the failure of a pulp treatment should be defined by outcomes with both clinical and radiological component outcomes. At least 50% of participants considered that 6 of the 15 component outcomes retained from round 1 should be part of the composite outcome defining failure of a pulp treatment. In all, 28% of participants considered 24 months as the optimal follow-up duration (27% of participants chose time to primary tooth exfoliation and 19% chose 36 months).

In total, 52 participants (25%) completed round 3. At least 70% of the participants considered that 5 of the 6 component outcomes retained from round 2 should be part of the composite outcome defining failure of a pulp treatment. The final core set of component outcome measures assessing the failure of pulp treatments in primary teeth included 3 clinical outcomes (soft tissue pathology, pain, pathologic mobility) and 2 radiological outcomes (pathologic radiolucency and pathologic root resorption). Moreover, 45% of participants considered 24 months as the optimal follow-up duration (31% of participants chose time to primary tooth exfoliation).

When assessing the reporting of the core set of outcomes for the RCTs, pathologic radiolucency and pathologic root resorption were described in all reports, pain and soft tissue pathology in 45 (96%), and pathologic mobility in 37 (75%). The definition of the composite success or failure outcome included the 5 outcomes of the core set in 20 reports (43%), 4 outcomes in 14 (30%), 3 outcomes in 4 (9%), 2 outcomes in 1 (2%) and 1 outcome in 1 report (2%). In 7 reports (15%), the definition of the composite success or failure outcome included none of the 5 outcomes of the core set (Supplementary Information, Figure S3).

## Discussion

Our systematic review of 47 reports of RCTs assessing the effectiveness of pulp treatments in primary teeth revealed great diversity in outcomes, specifically the component outcomes of a composite outcome defining success or failure of a pulp treatment. However, the small-group consensus process allowed for identifying several groups of similar outcomes, and the inventory of 83 outcomes was reduced to 24 outcome domains. Finally, the Delphi process allowed for identifying the 5 following component outcomes that should be part of a composite outcome defining failure of pulp treatments in primary teeth: pathologic radiolucency, soft tissue pathology, pain, pathologic mobility and pathologic root resorption. The minimum follow-up duration for outcomes was considered 12 or 24 months and the optimal follow-up duration was 24 months or time to natural exfoliation.

Several reasons may explain the diversity in reported outcomes and definition of success in included RCTs. First, after treatment, the state of primary teeth is monitored both clinically and radiologically. Clinical outcomes reflect symptoms experienced by patients. They may be dentist-assessed or patient-reported. However, carious primary teeth may be asymptomatic and radiological assessment may reveal clinically undetected failure of a pulp treatment. Assessing the 2 dimensions is consequently required and explained per se the multiplicity. Second, identical outcomes were sometimes referred to by different terms. The non-uniform terminology contributed artificially to the multiplicity issue. Moreover, RCTs used numerous different but close outcomes to assess the treatment effect on a similar outcome domain. Finally, the main reason for diversity was clearly the lack of a consensual definition of success or failure of a pulp treatment.

After reducing of overarching outcome domains by the small-group consensus and constructing a core set of 5 component outcomes by the Delphi process, the diverse outcomes were reduced. The 5 component outcomes were reported for almost all trials, except pathologic mobility, which was reported for 75% of trials. Moreover, about 75% of reports defined the composite success or failure outcome as including 4 or 5 outcomes of the core set. These component outcomes were considered the most relevant to define success or failure of a treatment. In fact, participants independently selected both clinical and radiological endpoints. These criteria allow for assessing the state of the tooth, perialveolar bone and periodontium, as well as patient-important outcomes, such as pain. Moreover, these 5 components are of similar clinical importance. Apart from the definition of success or failure, the most important outcome may be primary tooth survival because it may have the ability to reveal the benefits of treatment for patients. We found this outcome seldom reported in articles of RCTs (only 4%). This outcome ought to be investigated more frequently. However, a limitation of this outcome is that natural exfoliation or secondary treatment after failure (e.g., pulpectomy after failed pulpotomy) may not be taken into account when assessing whether the treated tooth was lost at 12 or 24 months after the initial treatment.

Because of heterogeneous selection and measurement of outcomes across trials, performing meta-analyses may be difficult if not impossible [20], [21]. Many meta-analyses frequently exclude a large number of trials because relevant outcomes are not reported [22]. The OMERACT group initiated the harmonization of outcomes, especially by the definition of core sets of outcomes to be measured in all trials of a specific condition [23] and is continuing on a broader scale with the COMET Initiative [24], [25]. In the field of dentistry, studies similar to ours revealed the diversity of outcomes and addressed the definition of adequate outcome measures in the field of dental implants [26], [27], [28], [29].

Our study has some limitations. First, we considered RCTs only in our review. However, a number of non-randomized trials, cohort studies or case series have assessed the effect of pulp therapies and consequently reported outcomes. Outcomes such as primary teeth survival may be more frequently reported in observational trials than in RCTs, but the inclusion of these studies may not have revealed additional efficacy outcomes. Our systematic review found a very large number of outcomes, and the inventory of outcomes also included outcomes that were retained in the Cochrane review but were not assessed in the included RCTs. The second limitation is the use of consensus processes to group similar outcomes and define a core set of component outcomes. These methods remain subjective, and methods to best develop core outcome sets still require development [15], [30], [31]. We defined consensus by percentage agreement (ie, >70%), but different methods exist and we lack guidelines. In addition, one-quarter of invited contacts completed round 3, which suggests possible attrition bias and that the degree of consensus reached in the final round may have been overestimated [32]. We were unable to determine the extent of such bias because we did not have any means to identify non-responders. However, the percentage of responders was stable during the 3 rounds. Moreover, we independently invited the 2 groups of experts (French dentists and international authors) and when analyzing Delphi results for these 2 groups, found that they selected the same 5 component outcomes. Third, we abstracted reported rather than pre-specified or measured outcomes. Selective outcome reporting bias in published RCT reports is common, and the frequency with which some outcomes were actually measured may be underestimated [33]. We could not assess trial protocols to correct this. However, this selective outcome reporting unlikely missed an outcome across all trials. Lastly, our consensus procedure involved clinicians only and didn't involve patients (children or parents). We acknowledge that informed clinical decisions could only be based on trials that have measured both clinician- and patient-important outcomes [15]. In previous studies, involvement of patients in the Delphi process identified outcomes as being of particular importance whereas they had not been measured in any of the included trials [24]. However, involvement of patients may have not revealed additional outcomes since we considered a variety of patient-important outcomes, such as pain which is part of the core set.

Our results could have implications for the design of primary research trials and the conduct of secondary research reviews. When designing future trials, investigators should be encouraged to define treatment success or failure using the 5 component outcomes retained in the core set of a composite outcome and, possibly, individually, and to assess and report primary tooth survival rates (eg, at 12 and 24 months). Given the limitations of composite outcomes, trialists should only analyze the pre-specified composite and report results for all the 5 components as recommended [34], [35]. For the synthesis of results across trials, our findings will ease the identification of similar outcomes across included trials that can be grouped in a meta-analysis. When assessing the effect of a pulp treatment on a given outcome domain, combining results from trials reporting data for different outcomes gathered under the corresponding domain would be meaningful. If a trial reported data for 2 or more outcomes of a given category, one could choose the outcome as the most frequent one in the meta-analysis. When assessing the effect of a pulp treatment on the composite outcome “failure”, our core set of component outcomes may ease the exploration of heterogeneity across trials. For instance, one could compare the treatment effect in the subgroup of RCT reports in which the definition of the composite success or failure outcome included 4 or 5 outcomes of the core set with the treatment effect in other reports.

## Conclusions

We found substantial diversity in outcomes reported in RCT articles of pulp treatment for primary teeth. Our consensus process revealed a core set of component outcomes to define failure of a pulp treatment in primary teeth. This core set of component outcomes defining the success/failure of pulp treatments in primary teeth may help in the design of future trials. The variability in selecting and measuring outcomes may be reduced. This core set of component outcomes would be useful for comparing results of trials and for performing systematic reviews, because similar (component) outcomes can be compared and pooled in the same meta-analysis if such outcomes have been assessed in several trials.

## Supporting Information

Figure S1 **Adjacency matrix of 83 outcomes in 47 reports of RCTs.**
(PPT)

Figure S2 **Network of 24 outcome domains in 47 reports of RCTs.** A: Pathologic radiolucency, B: Pathologic root resorption, C: Pain, D: Soft tissue pathology, E: Pathologic mobility, F: Pulp canal obliteration, G: Adjacent tissues inflammation, H: Dentine bridge formation, I: Defective restoration (clinically), J: Unerupted succedaneous tooth anomaly (radiographically), K: Secondary caries (clinically), L: Physiological resorption, M: Premature tooth loss, N: Periodontal pocket formation, O: Smell, P: Signs of exfoliation, Q: Filling material anomaly, R: Defective restoration (radiographically), S: Secondary caries (radiographically), T: Signs/symptoms of erupting succedaneous tooth, U: Erupting succedaneous tooth mobility, V: Succedaneous tooth structural anomaly. Each node in the figure represents each possible outcome domain (from the reduced inventory resulting from the small-group consensus process). The size of nodes was proportional to the number of trials that assessed the corresponding outcome. A vertex linked 2 nodes when the 2 respective outcome domains had been assessed together in the same trial. The width of vertices was proportional to the number of trials that assessed the 2 corresponding outcome domains. Two outcome domains are not represented because they were retained from the Cochrane review by Nadin et al. [10] but not assessed in the included RCTs.
(PPT)

Figure S3 **Network of 24 outcome domains defined as component outcomes of the success or failure composite outcome in the** **Methods** **section of 47 reports of RCTs.** A: Soft tissue pathology, B: Defective restoration (clinically), C: Unerupted succedaneous tooth anomaly (radiographically), D: Pain, E: Premature tooth loss, F: Smell, G: Signs of exfoliation, H: Pathologic radiolucency, I: Physiological resorption, J: Defective restoration (radiographically), K: secondary caries (radiographically), L: Pathologic mobility, M: Signs/symptoms of erupting succedaneous tooth, N: Erupting succedaneous tooth mobility, O: Secondary caries (clinically), P: Pathologic root resorption, Q: Periodontal pocket formation, R: Pulp canal obliteration, S: Dentine bridge formation. Five outcome domains are not represented, 2 were retained from the Cochrane review by Nadin et al. [10] but not assessed in the included RCTs and 3 were never defined in the Methods sections.
(PPT)

Table S1 **Methodological features of the Delphi process.**
(DOC)

Table S2 **Characteristics of each included randomized controlled trial (RCT).**
(DOC)

Table S3 **Reporting of the 83 outcomes identified in the 47 reports of RCTs.**
(DOC)

Table S4 **Details of the reporting of outcomes for each included RCT.**
(DOC)

Text S1 **Electronic search strategy.**
(DOC)

Text S2 **Included and excluded studies, and reasons for exclusion.**
(DOC)
