# Supplementary figures and images for: Development of a Core Set of Outcomes for Randomized Controlled Trials with Multiple Outcomes – Example of Pulp Treatments of Primary Teeth for Extensive Decay in Children
Source: PLoS One. 2013 Jan 3;8(1):e51908. doi: 10.1371/journal.pone.0051908 (PMC3536772; doi:10.1371/journal.pone.0051908)

## Slide 1
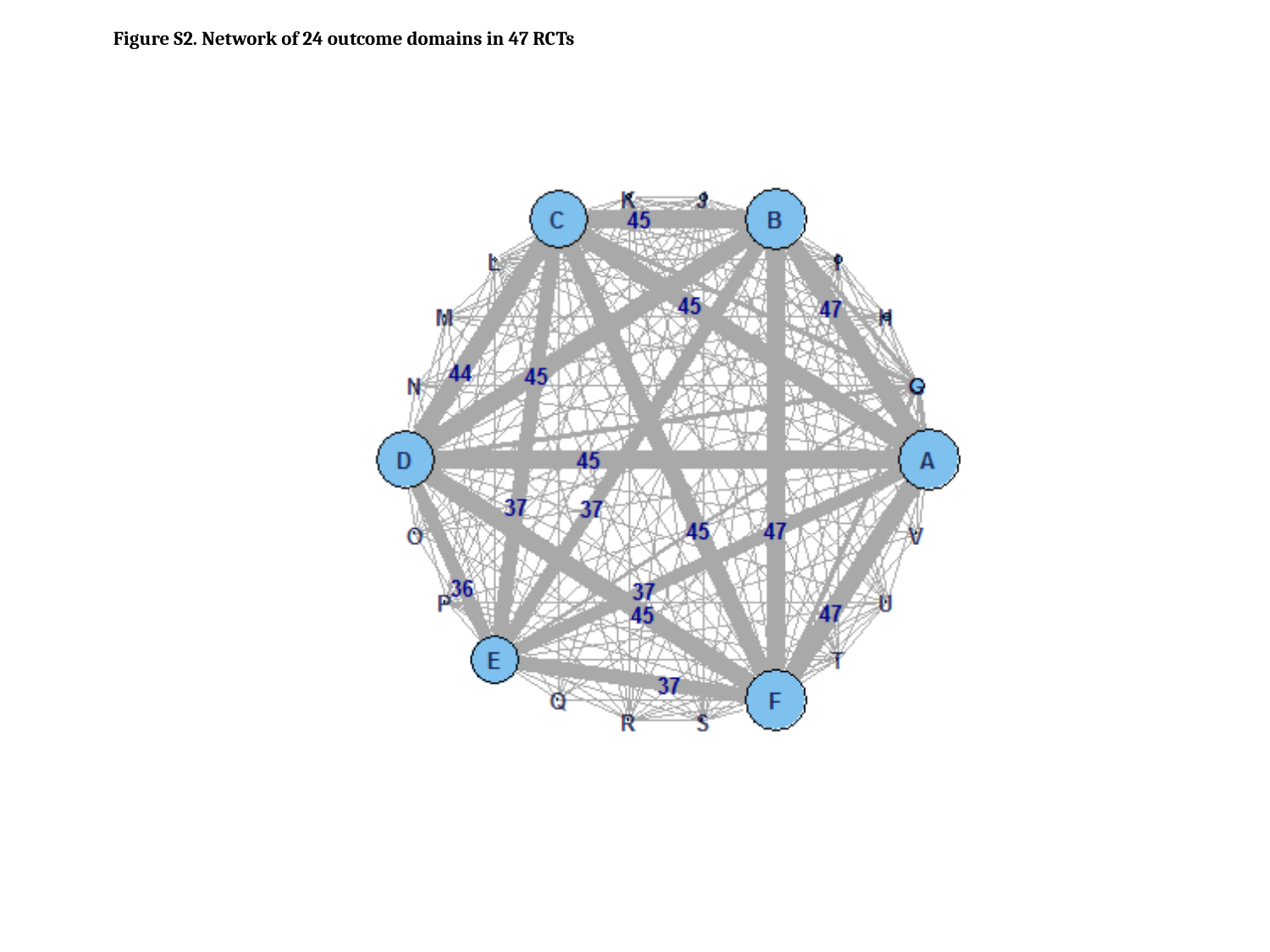

Figure S2. Network of 24 outcome domains in 47 RCTs

Supplement: Figure S2 — Network of 24 outcome domains in 47 reports of RCTs. A: Pathologic radiolucency, B: Pathologic root resorption, C: Pain, D: Soft tissue pathology, E: Pathologic mobility, F: Pulp canal obliteration, G: Adjacent tissues inflammation, H: Dentine bridge formation, I: Defective restoration (clinically), J: Unerupted succedaneous tooth anomaly (radiographically), K: Secondary caries (clinically), L: Physiological resorption, M: Premature tooth loss, N: Periodontal pocket formation, O: Smell, P: Signs of exfoliation, Q: Filling material anomaly, R: Defective restoration (radiographically), S: Secondary caries (radiographically), T: Signs/symptoms of erupting succedaneous tooth, U: Erupting succedaneous tooth mobility, V: Succedaneous tooth structural anomaly. Each node in the figure represents each possible outcome domain (from the reduced inventory resulting from the small-group consensus process). The size of nodes was proportional to the number of trials that assessed the corresponding outcome. A vertex linked 2 nodes when the 2 respective outcome domains had been assessed together in the same trial. The width of vertices was proportional to the number of trials that assessed the 2 corresponding outcome domains. Two outcome domains are not represented because they were retained from the Cochrane review by Nadin et al. [10] but not assessed in the included RCTs. (PPT) [file pone.0051908.s002.ppt]
