# Supplementary material for: Development of a Core Set of Outcomes for Randomized Controlled Trials with Multiple Outcomes – Example of Pulp Treatments of Primary Teeth for Extensive Decay in Children
Source: PLoS One. 2013 Jan 3;8(1):e51908. doi: 10.1371/journal.pone.0051908 (PMC3536772; doi:10.1371/journal.pone.0051908)
Supplement: Figure S3 — Network of 24 outcome domains defined as component outcomes of the success or failure composite outcome in the Methods section of 47 reports of RCTs. A: Soft tissue pathology, B: Defective restoration (clinically), C: Unerupted succedaneous tooth anomaly (radiographically), D: Pain, E: Premature tooth loss, F: Smell, G: Signs of exfoliation, H: Pathologic radiolucency, I: Physiological resorption, J: Defective restoration (radiographically), K: secondary caries (radiographically), L: Pathologic mobility, M: Signs/symptoms of erupting succedaneous tooth, N: Erupting succedaneous tooth mobility, O: Secondary caries (clinically), P: Pathologic root resorption, Q: Periodontal pocket formation, R: Pulp canal obliteration, S: Dentine bridge formation. Five outcome domains are not represented, 2 were retained from the Cochrane review by Nadin et al. [10] but not assessed in the included RCTs and 3 were never defined in the Methods sections. (PPT) [file pone.0051908.s003.ppt]

## Slide 1
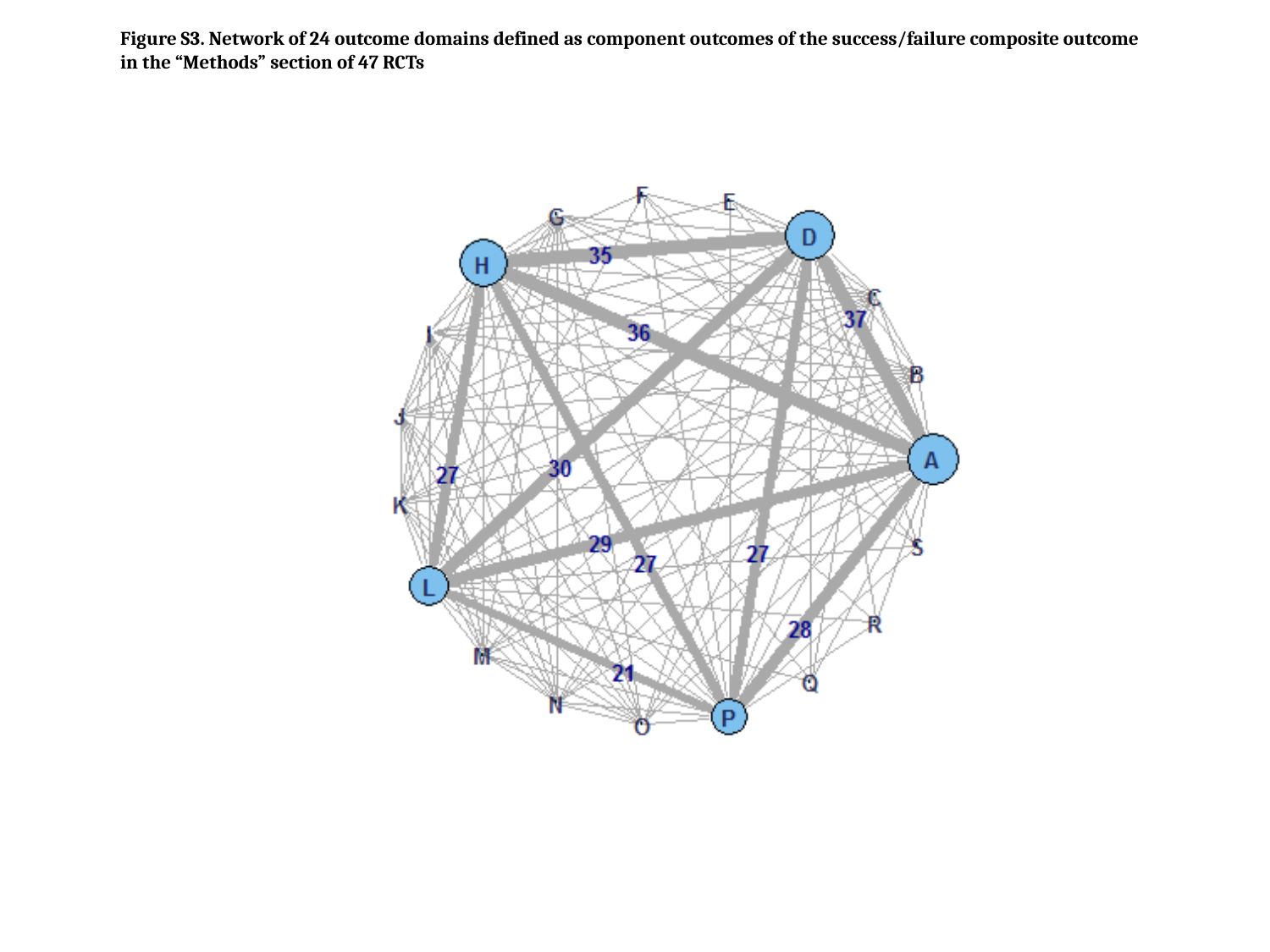

Figure S3. Network of 24 outcome domains defined as component outcomes of the success/failure composite outcome
in the “Methods” section of 47 RCTs
