# Supplementary material for: Development of a Core Set of Outcomes for Randomized Controlled Trials with Multiple Outcomes – Example of Pulp Treatments of Primary Teeth for Extensive Decay in Children
Source: PLoS One. 2013 Jan 3;8(1):e51908. doi: 10.1371/journal.pone.0051908 (PMC3536772; doi:10.1371/journal.pone.0051908)
Supplement: Table S1 — Methodological features of the Delphi process. (DOC) [file pone.0051908.s004.doc]

Table S1. Methodological features of the Delphi process

Sinha et al. included 15 studies in their systematic review and identified variations between these studies in terms of composition of the group, anonymity, structure of the Delphi process, how consensus was reached about which outcomes to measure, and attrition of participants [Sinha IP, Smyth RL, Williamson PR (2011) Using the Delphi technique to determine which outcomes to measure in clinical trials: recommendations for the future based on a systematic review of existing studies. PLoS Med 8: e1000393]. The bolded methodological features are those we chose for our study. The methods chosen are the most frequently used, except for the identification of potential outcomes, but we identified outcomes by a systematic review, which is the most rigorous method.

| **Methodological features** | **Frequency among the 15 studies selected by Sinha et al. [references to respective studies]** |
| --- | --- |
| *Composition of the group* |  |
| Studies were conducted by clinical trials networks | 8 [1-8] |
| **Clinicians who had published research in the relevant field were included** | **4 [2, 9-11]** |
| **Clinicians and researchers were included** | **3 [12-14]** |
| *Method by which the Delphi study was conducted* |  |
| **Email, post or internet** | **13 [1-8, 10-13, 15]** |
| Face-to-face meetings | 2 [9, 14] |
| *Anonymity* |  |
| **Completely anonymously** | **7 [2, 5, 7, 10-12, 15]** |
| Complete anonymity is presumed | 6 [1, 3, 4, 6, 8, 13] |
| *Identification of potential outcomes* |  |
| Outcomes were suggested by participants | 4 [5, 11, 13, 15] |
| **Outcomes were suggested to participants** | **3 [2, 7, 8]** |
| Outcomes were identified at international meetings | 3 [4, 7, 9] |
| *Determining the importance of potential outcomes* |  |
| **Participants scored the importance of each outcome (round 1)** | **6 [1, 7, 10, 12, 13, 15]** |
| **Percentage of people voting for outcome inclusion in the core set (rounds 2 and 3)** | **5 [2, 4, 8, 9, 14]** |
| Participants distributed a set number of points among outcomes, according to importance | 2 [3, 8] |
| Participants ranked outcomes in order of importance | 2 [5, 11] |
| *Feedback of the results to participants (second round)* |  |
| **Fed back the average score for each outcome (round 2)** | **7 [3, 6, 7, 10, 12, 13, 15]** |
| **Percentage of people voting for outcome inclusion in the core set (round 3)** | **1** |
| Facilitators analyzed data and presented a new list of outcomes, without presenting a measure of group opinion to participants | 4 [1, 2, 5, 11] |
| *How consensus was reached about which outcomes to measure* | |
| Outcomes received a pre-determined score | 2 [1, 7] |
| **A pre-determined proportion of participants considered that the outcome should be included in a core set** | **4 [2, 8, 9, 14]** |
| Delphi process not used to reach consensus: results used to inform people participating in subsequent round | 6 [3-6, 11, 13] |
| *Attrition of participants* |  |
| **Each participant was invited to participate in every round, even if they did not complete previous questionnaires** | **5 [2, 3, 5, 8, 10]** |
| People who did not respond to any given round were excluded from the remainder of the study | 3 [1, 7, 12] |
| Additional participants were invited as the Delphi progressed | 2 [4, 13] |

***Studies***

1. Khanna, D., et al., Development of a provisional core set of response measures for clinical trials of systemic sclerosis. Ann Rheum Dis, 2008. 67(5): p. 703-9.

2. Lux, A.L. and J.P. Osborne, A proposal for case definitions and outcome measures in studies of infantile spasms and West syndrome: consensus statement of the West Delphi group. Epilepsia, 2004. 45(11): p. 1416-28.

3. Mease, P.J., et al., Identifying the clinical domains of fibromyalgia: contributions from clinician and patient Delphi exercises. Arthritis Rheum, 2008. 59(7): p. 952-60.

4. Miller, F.W., et al., Proposed preliminary core set measures for disease outcome assessment in adult and juvenile idiopathic inflammatory myopathies. Rheumatology (Oxford), 2001. 40(11): p. 1262-73.

5. Ruperto, N., et al., Preliminary core sets of measures for disease activity and damage assessment in juvenile systemic lupus erythematosus and juvenile dermatomyositis. Rheumatology (Oxford), 2003. 42(12): p. 1452-9.

6. Taylor, W.J., Preliminary identification of core domains for outcome studies in psoriatic arthritis using Delphi methods. Ann Rheum Dis, 2005. 64 Suppl 2: p. ii110-2.

7. Taylor, W.J., et al., A modified Delphi exercise to determine the extent of consensus with OMERACT outcome domains for studies of acute and chronic gout. Ann Rheum Dis, 2008. 67(6): p. 888-91.

8. Zochling, J., et al., Development of a core set of domains for data collection in cohorts of patients with ankylosing spondylitis receiving anti-tumor necrosis factor-alpha therapy. J Rheumatol, 2008. 35(6): p. 1079-82.

9. Dent, J., et al., Clinical trial design in adult reflux disease: a methodological workshop. Aliment Pharmacol Ther, 2008. 28(1): p. 107-26.

10. Distler, O., et al., Defining appropriate outcome measures in pulmonary arterial hypertension related to systemic sclerosis: a Delphi consensus study with cluster analysis. Arthritis Rheum, 2008. 59(6): p. 867-75.

11. McGrath, P.J., et al., Core outcome domains and measures for pediatric acute and chronic/recurrent pain clinical trials: PedIMMPACT recommendations. J Pain, 2008. 9(9): p. 771-83.

12. Devane, D., et al., Evaluating maternity care: a core set of outcome measures. Birth, 2007. 34(2): p. 164-72.

13. Douglas, R.S., et al., Development of criteria for evaluating clinical response in thyroid eye disease using a modified Delphi technique. Arch Ophthalmol, 2009. 127(9): p. 1155-60.

14. Khan, F. and J.F. Pallant, Use of the International Classification of Functioning, Disability and Health (ICF) to identify preliminary comprehensive and brief core sets for multiple sclerosis. Disabil Rehabil, 2007. 29(3): p. 205-13.

15. Serrano-Aguilar, P., et al., Patient involvement in health research: a contribution to a systematic review on the effectiveness of treatments for degenerative ataxias. Soc Sci Med, 2009. 69(6): p. 920-5.
