# Supplementary material for: Development of a Core Set of Outcomes for Randomized Controlled Trials with Multiple Outcomes – Example of Pulp Treatments of Primary Teeth for Extensive Decay in Children
Source: PLoS One. 2013 Jan 3;8(1):e51908. doi: 10.1371/journal.pone.0051908 (PMC3536772; doi:10.1371/journal.pone.0051908)
Supplement: Table S2 — Characteristics of each included randomized controlled trial (RCT). (DOC) [file pone.0051908.s005.doc]

Table S2. Characteristics of each randomized controlled trial (RCT)

| Study | Overall risk of bias | Number of randomly allocated teeth | Number of arms | Interventions compared* | Duration of follow-up (months) |
| --- | --- | --- | --- | --- | --- |
| Aeinehchi, 2007 | High | 126 | 2 | 1-1 vs. 1-7 | Fixed: 3, 6 |
| Agamy, 2004 | High | 72 | 3 | 1-1 vs. 1-2 vs. 1-3 | Fixed: 3, 6, 12 |
| Alaçam, 1989 | Unclear | 100 | 3 | 1-1 vs. 1-4 vs. 1-5 | Fixed: 3, 6, 9, 12 |
| Alaçam, 2009 | Unclear | 69 | 3 | 1-1 vs. 1-13 vs. 1-14 | Fixed: 3 |
| Aminabadi, 2010 | Unclear | 120 | 2 | 3-1 vs. 3-4 | Fixed: 6, 12, 18, 24 |
| Ansari, 2010 | High | 40 | 2 | 1-1 vs. 1-7 | Fixed: 6, 12, 24 |
| Bahrololoomi, 2008 | Unclear | 70 | 2 | 1-1 vs. 1-6 | Fixed: 3, 6, 9 |
| Casas, 2004 | High | 291 | 2 | 1-9 vs. 2-24 | Fixed: 24, 36 |
| Coser, 2008 | Unclear | 51 | 2 | 1-1 vs. 2-4 | Fixed: 12 |
| Dean, 2002 | Unclear | 50 | 2 | 1-1 vs. 1-6 | Variable**: 11.2 [5.5-28] |
| Demir, 2007 | Unclear | 100 | 5 | 3-4 vs. 3-15 vs. 3-16 vs. 3-17 vs. 3-18 | Fixed: 3, 6, 9, 12, 18, 24 |
| Doyle, 2010 | High | 266 | 4 | 1-7 vs. 1-9 vs. 1-28 vs. 1-29 | Variable: 22 [6-38] |
| Eidelman, 2001 | High | NA | 2 | 1-1 vs. 1-7 | Variable: 13 [6-31] |
| Erdem, 2011 | Unclear | 100 | 4 | 1-1 vs. 1-7 vs. 1-9 vs. 1-21 | Fixed: 6, 12, 24 |
| Farsi, 2005 | High | 120 | 2 | 1-1 vs. 1-7 | Fixed: 6, 12, 18, 24 |
| Fei, 1991 | High | NA | 2 | 1-1 vs. 1-9 | Fixed: 3, 6, 12 |
| Fishman, 1996 | Unclear | 47 | 2 | 1-31 vs. 1-32 | Fixed: 3, 6 |
| Fuks, 1997 | Unclear | 96 | 2 | 1-1 vs. 1-9 | Variable: 12 [6-35] |
| Garrocho-Rangel, 2009 | Low | 90 | 2 | 3-4 vs. 3-8 | Fixed: 6, 12 |
| Holan, 2005 | Unclear | 64 | 2 | 1-1 vs. 1-7 | Variable: 36 [4-74] |
| Huth, 2010 | Unclear | 191 | 4 | 1-1 vs. 1-4 vs. 1-9 vs. 1-19 | Fixed: 6, 12, 18, 24 |
| Ibricevic, 2003 | High | 164 | 2 | 1-1 vs. 1-9 | Variable: [3-48] |
| Malekafzali, 2011 | High | 80 | 2 | 1-7 vs. 1-40 | Fixed: 6, 12, 24 |
| Markovic, 2005 | Unclear | 104 | 3 | 1-1 vs. 1-4 vs. 1-9 | Fixed: 18 |
| Moretti, 2008 | Unclear | 45 | 3 | 1-1 vs. 1-4 vs. 1-7 | Fixed: 3, 6, 12, 18, 24 |
| Mortazavi, 2004 | High | 58 | 2 | 2-21 vs. 2-22 | Variable: 12 [10-16] |
| Nadkarni, 2000 | Unclear | 70 | 2 | 2-4 vs. 2-21 | Fixed: 3, 6, 9 |
| Naik, 2005 | Unclear | 50 | 2 | 1-1 vs. 1-7 | Fixed: 3, 6 |
| Nakornchai, 2010 | Unclear | 50 | 2 | 2-22 vs. 2-30 | Fixed: 6, 12 |
| Noorollahian, 2008 | High | 60 | 2 | 1-1 vs. 1-3 | Fixed: 6, 12, 24 |
| Ozalp, 2005 | Unclear | 80 | 4 | 2-21 vs. 2-22 vs. 2-26 vs. 2-27 | Fixed: 6, 12, 18 |
| Pinky, 2012 | Unclear | 40 | 2 | 2-35 vs. 2-36 | Fixed: 3, 6, 12 |
| Prabhakar, 2008 | Unclear | 60 | 2 | 1-10 vs. 1-11 | Fixed: 6, 12 |
| Ramar, 2010 | Unclear | 96 | 3 | 2-5 vs. 2-33 vs. 2-34 | Fixed: 3, 6, 9 |
| Sabbarini, 2008 | Unclear | 30 | 2 | 1-1 vs. 1-8 | Fixed: 6 |
| Sakai, 2009 | High | 30 | 2 | 1-7 vs. 1-12 | Fixed: 6, 12, 18, 24 |
| Saltzman, 2005 | High | 52 | 2 | 1-1 vs. 1-20 | Variable: [2.3-15.7] |
| Shumayrikh, 1999 | Unclear | 61 | 2 | 1-13 vs. 1-14 | Fixed: 12 |
| Sonmez, 2008 | High | 56 | 4 | 1-1 vs. 1-4 vs. 1-7 vs. 1-9 | Fixed: 6, 12, 18, 24 |
| Subramaniam, 2009 | Unclear | 40 | 2 | 1-1 vs. 1-7 | Fixed: 6, 12, 24 |
| Subramaniam, 2011 | Unclear | 45 | 3 | 2-5 vs. 2-21 vs. 2-39 | Fixed: 3, 6, 12, 18 |
| Trairatvorakul, 2008 | Unclear | 54 | 2 | 2-21 vs. 2-22 | Fixed: 6, 12 |
| Tuna, 2008 | High | 50 | 2 | 3-4 vs. 3-7 | Fixed: 3, 6, 9, 12, 18, 24 |
| Vargas, 2006 | High | 60 | 2 | 1-9 vs. 1-23 | Fixed: 6, 12 |
| Waterhouse, 2002 | Unclear | 84 | 2 | 1-1 vs. 1-4 | Fixed: 6, 12 |
| Zealand, 2010 | High | 252 | 2 | 1-1 vs. 1-2 | Fixed: 6 |
| Zurn, 2008 | High | 68 | 2 | 1-1 vs. 1-25 | Variable: [6-24] |

** Interventions compared: - first number 1/2/3 vs. …: type of pup treatment: 1: pulpotomy; 2: pulpectomy; 3: direct pulp capping*

*- second number … vs. 1/[…]/40: biomaterial applied after pulp treatment: 1: formocresol; 2: GMTA (gray mineral trioxide aggregate); 3: WTMA (white mineral trioxide aggregate); 4: calcium hydroxide; 5: calcium hydroxide/iodoform (METAPEX); 6: electrosurgery; 7: MTA; 8: Enamel Matrix Derivative (EMD); 9: ferric sulfate; 10: only the necrotic coronal pulp removed + antibacterial mix (ciprofloxacin+metronidazole+minocycline); 11: both necrotic coronal as well as all accessible radicular pulp tissue extirpated + antibacterial mix (ciprofloxacin+metronidazole+minocycline);12: Portland cement; 13: glutaraldehyde with ZOE; 14: glutaraldehyde with calcium hydroxide; 15: acetone-based total-etch adhesive; 16: non-rinse conditioner and treatment 15; 17: total-etching with 36% phosphoric acid followed by treatment 15; 18: self-etch adhesive system; 19: Erbium: Yttrium-Aluminium Garnet (Er:YAG laser); 20: diode laser with MTA; 21: zinc oxide-eugenol (ZOE); 22: calcium hydroxide/iodoform paste (Vitapex); 23: 5% sodium hypochlorite (NaOCL); 24: Sedanol; 25: light-cured calcium hydroxide; 26: Sealapex; 27: Calcicur; 28: eugenol-free ferric sulfate; 29: FS:MTA; 30: 3Mix; 31: electrofulguration + ZOE; 32: electrofulguration + calcium hydroxide; 33: ZOE with iodoform (RC FILL); 34: ZOE and CH with iodoform; 35: ciprofloxacin+metronidazole+minocycline; 36: ciprofloxacin+ornidazole+minocycline; 37: formocresol+subbase of ZOE and formocresol; 38: formocresol+ZOE; 39: Endoflas; 40: calcium-enriched mixture (CEM)*

*** Duration of follow-up: variable [min-max]*
