# Supplementary material for: Development of a Core Set of Outcomes for Randomized Controlled Trials with Multiple Outcomes – Example of Pulp Treatments of Primary Teeth for Extensive Decay in Children
Source: PLoS One. 2013 Jan 3;8(1):e51908. doi: 10.1371/journal.pone.0051908 (PMC3536772; doi:10.1371/journal.pone.0051908)
Supplement: Table S3 — Reporting of the 83 outcomes identified in the 47 reports of RCTs. (DOC) [file pone.0051908.s006.doc]

Table S3. Reporting of each the 83 outcomes in the 47 selected RCTs

| Outcome | Frequency | % |
| --- | --- | --- |
| *Clinical outcomes concerning primary teeth* |  |  |
| Pathologic mobility | 37 | 79 |
| Pain symptom | 35 | 74 |
| Swelling | 31 | 66 |
| Tenderness to percussion | 30 | 64 |
| Fistula | 27 | 57 |
| Sinus tract | 21 | 45 |
| Spontaneous pain | 19 | 40 |
| Abscess | 16 | 34 |
| Soft tissue pathology/swelling | 7 | 15 |
| Inflammation in the adjacent tissues | 5 | 11 |
| Secondary caries at the margin | 4 | 9 |
| Redness around the tooth/crown | 4 | 9 |
| Parulis | 4 | 9 |
| Thermal sensitivity | 3 | 6 |
| Pain on palpation | 3 | 6 |
| Premature tooth loss | 3 | 6 |
| Perforated or lost final restoration | 3 | 6 |
| Pain initiated by stimuli | 2 | 4 |
| Smell | 2 | 4 |
| Defective restoration | 2 | 4 |
| Severe gingival inflammation | 2 | 4 |
| Restoration intact | 2 | 4 |
| Erythema | 2 | 4 |
| Periodontal pocket formation (exudate or no exudate) | 2 | 4 |
| Signs of exfoliation | 2 | 4 |
| Sensitivity to pressure | 1 | 2 |
| Edema | 1 | 2 |
| Marginal integrity | 1 | 2 |
| Partially or completely lost fillings | 1 | 2 |
| Changes in the appearance of surrounding tissue | 1 | 2 |
| Bleeding around the tooth/crown | 1 | 2 |
| Chewing sensitivity | 1 | 2 |
| Extraoral swelling | 1 | 2 |
| Sensitivity to sour | 1 | 2 |
| Intraoral swelling | 1 | 2 |
| Sensitivity to sweet | 1 | 2 |
| Infection in the adjacent tissues | 1 | 2 |
| Score 1 | 1 | 2 |
| Score 2 | 1 | 2 |
| *Radiological outcomes concerning primary teeth* |  |  |
| Furcal/bifurcation radiolucency | 36 | 77 |
| Internal root resorption | 35 | 74 |
| External root resorption | 26 | 55 |
| Periapical radiolucency | 24 | 51 |
| Pulp canal obliteration | 23 | 49 |
| Periodontal ligament space widening | 18 | 38 |
| Periapical bone destruction | 10 | 21 |
| Pathologic root resorption | 8 | 17 |
| Dentine bridge formation | 7 | 15 |
| Interradicular bone destruction | 6 | 13 |
| Periradicular radiolucency | 5 | 11 |
| Integrity of lamina dura | 5 | 11 |
| Calcific metamorphosis | 5 | 11 |
| Furcation involvement | 4 | 9 |
| Root resorption in relation to contralateral tooth | 4 | 9 |
| Physiological resorption | 4 | 9 |
| Radicular radiolucency | 3 | 6 |
| Pathologic radiolucency | 3 | 6 |
| Intra-radicular radiolucency | 2 | 4 |
| Internal root resorption – perforated form | 2 | 4 |
| Replacement resorption | 2 | 4 |
| Excess filling material and its resorption | 2 | 4 |
| Bone radiolucency | 2 | 4 |
| Defective restoration | 2 | 4 |
| Recurrent caries | 2 | 4 |
| Lateral radiolucency | 1 | 2 |
| Apical radiolucency | 1 | 2 |
| Involvement of the apical area | 1 | 2 |
| Root resorption in relation to contralateral tooth with criteria established by Wright | 1 | 2 |
| Abnormal inter-radicular trabeculation/variation in radiodensity | 1 | 2 |
| Calcific degeneration of the pulp | 1 | 2 |
| Loss of trabecular bone | 1 | 2 |
| Internal dentine resorption | 1 | 2 |
| Bone regeneration | 1 | 2 |
| Abnormalities in the structure of trabecular bone | 1 | 2 |
| Score 1 | 1 | 2 |
| Score 2 | 1 | 2 |
| Score 3 | 1 | 2 |
| Score 4 | 1 | 2 |
| *Outcomes concerning permanent succedaneous teeth* |  |  |
| Position and eruption pathway of the permanent successor tooth | 4 | 9 |
| Signs/symptoms of the successor tooth erupting | 2 | 4 |
| Mobility of the successor tooth erupting | 2 | 4 |
| Damage to succedaneous follicle | 2 | 4 |
| Succedaneous tooth structural anomaly | 2 | 4 |
