# Supplementary material for: Development of a Core Set of Outcomes for Randomized Controlled Trials with Multiple Outcomes – Example of Pulp Treatments of Primary Teeth for Extensive Decay in Children
Source: PLoS One. 2013 Jan 3;8(1):e51908. doi: 10.1371/journal.pone.0051908 (PMC3536772; doi:10.1371/journal.pone.0051908)
Supplement: Table S4 — Details of the reporting of outcomes for each included RCT. (DOC) [file pone.0051908.s007.doc]

Table S4. Details of reporting of outcomes for each selected RCT

|  | All outcomes | Clinical outcomes | Radiological outcomes | Other outcomes | Outcomes defined in Methods and reported in Results | Outcomes defined in Methods but not reported in Results | Outcomes not defined in Methods but reported in Results | Component outcomes | Component outcomes defined in Methods and reported in Results | Component outcomes defined in Methods but not reported in Results |
| --- | --- | --- | --- | --- | --- | --- | --- | --- | --- | --- |
| Aeinehchi, 2007 | 10 | 5 | 5 | 0 | 4 | 6 | 0 | 10 | 4 | 6 |
| Agamy, 2004 | 11 | 6 | 5 | 0 | 2 | 8 | 1 | 9 | 1 | 8 |
| Alaçam, 1989 | 12 | 7 | 5 | 0 | 5 | 3 | 4 | 8 | 5 | 3 |
| Alaçam, 2009 | 14 | 8 | 5 | 1 | 2 | 8 | 4 | 0 | 0 | 0 |
| Aminabadi, 2010 | 22 | 11 | 9 | 2 | 5 | 14 | 3 | 19 | 5 | 14 |
| Ansari, 2010 | 11 | 5 | 6 | 0 | 5 | 0 | 6 | 5 | 5 | 0 |
| Bahrololoomi, 2008 | 11 | 4 | 7 | 0 | 5 | 4 | 2 | 9 | 5 | 4 |
| Casas, 2004 | 17 | 10 | 7 | 0 | 11 | 4 | 2 | 0 | 0 | 0 |
| Coser, 2008 | 1 | 0 | 1 | 0 | 1 | 0 | 0 | 0 | 0 | 0 |
| Dean, 2002 | 8 | 4 | 4 | 0 | 0 | 8 | 0 | 8 | 0 | 8 |
| Demir, 2007 | 12 | 8 | 4 | 0 | 7 | 4 | 1 | 10 | 6 | 4 |
| Doyle, 2010 | 20 | 10 | 10 | 0 | 8 | 9 | 3 | 0 | 0 | 0 |
| Eidelman, 2001 | 7 | 3 | 4 | 0 | 2 | 5 | 0 | 6 | 1 | 5 |
| Erdem, 2011 | 9 | 4 | 5 | 0 | 3 | 5 | 1 | 7 | 3 | 4 |
| Farsi, 2005 | 7 | 3 | 4 | 0 | 7 | 0 | 0 | 6 | 6 | 0 |
| Fei, 1991 | 12 | 5 | 7 | 0 | 1 | 10 | 1 | 11 | 1 | 10 |
| Fishman, 1996 | 13 | 6 | 7 | 0 | 5 | 5 | 3 | 10 | 5 | 5 |
| Fuks, 1997 | 9 | 3 | 6 | 0 | 4 | 4 | 1 | 6 | 2 | 4 |
| Garrocho-Rangel, 2009 | 8 | 5 | 3 | 0 | 3 | 3 | 2 | 6 | 3 | 3 |
| Holan, 2005 | 10 | 2 | 8 | 0 | 4 | 3 | 3 | 5 | 2 | 3 |
| Huth, 2010 | 15 | 10 | 5 | 0 | 9 | 5 | 1 | 10 | 9 | 1 |
| Ibricevic, 2003 | 16 | 7 | 8 | 1 | 2 | 13 | 1 | 14 | 2 | 12 |
| Malekafzali, 2011 | 11 | 5 | 6 | 0 | 1 | 10 | 0 | 9 | 1 | 8 |
| Markovic, 2005 | 13 | 5 | 8 | 0 | 9 | 2 | 2 | 10 | 8 | 2 |
| Moretti, 2008 | 10 | 6 | 4 | 0 | 5 | 3 | 2 | 8 | 5 | 3 |
| Mortazavi, 2004 | 7 | 5 | 1 | 1 | 6 | 1 | 0 | 6 | 6 | 0 |
| Nadkarni, 2000 | 4 | 3 | 1 | 0 | 4 | 0 | 0 | 4 | 4 | 0 |
| Naik, 2005 | 10 | 4 | 6 | 0 | 0 | 0 | 10 | 0 | 0 | 0 |
| Nakornchai, 2010 | 13 | 8 | 5 | 0 | 8 | 1 | 4 | 8 | 7 | 1 |
| Noorollahian, 2008 | 14 | 6 | 8 | 0 | 1 | 12 | 1 | 12 | 0 | 12 |
| Ozalp, 2005 | 11 | 6 | 5 | 0 | 5 | 5 | 1 | 10 | 5 | 5 |
| Pinky, 2012 | 6 | 5 | 1 | 0 | 4 | 1 | 1 | 5 | 4 | 1 |
| Prabhakar, 2008 | 8 | 6 | 2 | 0 | 3 | 3 | 2 | 6 | 3 | 3 |
| Ramar, 2010 | 16 | 7 | 8 | 1 | 3 | 11 | 2 | 0 | 0 | 0 |
| Sabbarini, 2008 | 11 | 6 | 5 | 0 | 4 | 6 | 1 | 10 | 4 | 6 |
| Sakai, 2009 | 11 | 7 | 4 | 0 | 6 | 3 | 2 | 8 | 5 | 3 |
| Saltzman, 2005 | 8 | 3 | 4 | 1 | 2 | 4 | 2 | 6 | 2 | 4 |
| Shumayrikh, 1999 | 10 | 5 | 5 | 0 | 10 | 0 | 0 | 10 | 10 | 0 |
| Sonmez, 2008 | 12 | 6 | 6 | 0 | 4 | 7 | 1 | 10 | 3 | 7 |
| Subramaniam, 2009 | 12 | 6 | 6 | 0 | 3 | 9 | 0 | 10 | 1 | 9 |
| Subramaniam, 2011 | 12 | 8 | 3 | 1 | 6 | 5 | 1 | 11 | 6 | 5 |
| Trairatvorak ul, 2008 | 12 | 6 | 5 | 1 | 3 | 6 | 3 | 9 | 3 | 6 |
| Tuna, 2008 | 10 | 6 | 4 | 0 | 0 | 9 | 1 | 9 | 0 | 9 |
| Vargas, 2006 | 13 | 9 | 4 | 0 | 6 | 5 | 2 | 11 | 6 | 5 |
| Waterhouse, 2002 | 22 | 10 | 9 | 3 | 11 | 9 | 2 | 20 | 11 | 9 |
| Zealand, 2010 | 20 | 11 | 9 | 0 | 8 | 12 | 0 | 0 | 0 | 0 |
| Zurn, 2008 | 17 | 7 | 10 | 0 | 6 | 9 | 2 | 1 | 0 | 1 |
