# Supplementary material for: Development of a Core Set of Outcomes for Randomized Controlled Trials with Multiple Outcomes – Example of Pulp Treatments of Primary Teeth for Extensive Decay in Children
Source: PLoS One. 2013 Jan 3;8(1):e51908. doi: 10.1371/journal.pone.0051908 (PMC3536772; doi:10.1371/journal.pone.0051908)
Supplement: Text S1 — Electronic search strategy. (DOC) [file pone.0051908.s008.doc]

**Text S1. Electronic search strategy**

**COCHRANE ORAL HEALTH GROUP TRIALS** **REGISTER SEARCH STRATEGY**

((DENTAL-PULP-CAPPING OR "pulp cap*" OR PULPECTOMY OR PULPOTOMY OR pulpectom* OR pulpotom* OR "ROOT-CANAL-THERAPY" OR ("root canal" AND (therapy or treatment)) OR ENDODONTICS OR endodontic*) AND (primary or deciduous OR milk OR baby OR temporary OR natal OR child* OR infant* OR child-preschool))

**CENTRAL SEARCH STRATEGY**

#1 DENTAL PULP CAPPING single term (MeSH)

#2 PULPECTOMY single term (MeSH)

#3 PULPOTOMY single term (MeSH)

#4 ROOT CANAL THERAPY explode all trees (MeSH)

#5 ENDODONTICS single term (MeSH)

#6 (#1 or #2 or #3 or #4 or #5)

#7 ((root next canal) and (therap* or treat*))

#8 pulpectom*

#9 pulpotom*

#10 (pulp near cap*)

#11 (#7 or #8 or #9 or #10)

#12 (primary or deciduous or milk or baby or temporary or natal)

#13 CHILD explode (MeSH)

#14 child* or infant*

#15 #12 or #13 or #14

#16 #6 or #11

#17 (#15 AND #16)

**MEDLINE via OVID SEARCH STRATEGY**

1. Dental Pulp Capping/
2. PULPECTOMY/
3. PULPOTOMY/
4. exp "Root Canal Therapy"/
5. ENDODONTICS/
6. or/1-5
7. (root canal and (therap$ or treat$)).mp. [mp=title, original title, abstract, name of substance, mesh subject heading]
8. (pulpectom$ or pulpotom$).mp. [mp=title, original title, abstract, name of substance, mesh subject heading]
9. (pulp adj6 cap$).mp. [mp=title, original title, abstract, name of substance, mesh subject heading]
10. or/7-9
11. (primary or deciduous or milk or baby or temporary or natal).mp. [mp=title, original title, abstract, name of substance, mesh subject heading]
12. exp Child/
13. Infant/
14. (child$ or infant$).mp. [mp=title, original title, abstract, name of substance, mesh subject heading]
15. or/11-14
16. 6 or 10
17. 15 and 16

# Cochrane Search filter for MEDLINE via OVID

*Cochrane Highly Sensitive Search Strategy (CHSSS) for identifying randomized trials in MEDLINE: sensitivity maximising version (2008 revision) as referenced in Chapter 6.4.11.1 and detailed in box 6.4.c of The Cochrane Handbook for Systematic Reviews of Interventions Version 5.1.0 [updated March 2011].*

1. randomized controlled trial.pt.

2. controlled clinical trial.pt.

3. randomized.ab.

4. placebo.ab.

5. drug therapy.fs.

6. randomly.ab.

7. trial.ab.

8. groups.ab.

9. or/1-8

10. exp animals/ not humans.sh.

11. 9 not 10

**EMBASE via OVID SEARCH STRATEGY**

1. endodontics/
2. (pulp adj6 cap$).mp. [mp=title, abstract, subject headings, drug trade name, original title, device manufacturer, drug manufacturer name]
3. (pulpectom$ or pulpotom$).mp. [mp=title, abstract, subject headings, drug trade name, original title, device manufacturer, drug manufacturer name]
4. ((root adj canal) and (therap$ or treat$)).mp. [mp=title, abstract, subject headings, drug trade name, original title, device manufacturer, drug manufacturer name]
5. or/1-4
6. (primary or deciduous or milk or baby or temporary or natal).mp. [mp=title, abstract, subject headings, drug trade name, original title, device manufacturer, drug manufacturer name]
7. Child/
8. Infant/
9. (child$ or infant$).mp. [mp=title, abstract, subject headings, drug trade name, original title, device manufacturer, drug manufacturer name]
10. or/6-9
11. 5 and 10

# The above subject search was linked to the following filter for EMBASE via OVID:

1. random$.ti,ab.
2. factorial$.ti,ab.
3. (crossover$ or cross over$ or cross-over$).ti,ab.
4. placebo$.ti,ab.
5. (doubl$ adj blind$).ti,ab.
6. (singl$ adj blind$).ti,ab.
7. assign$.ti,ab.
8. allocat$.ti,ab.
9. volunteer$.ti,ab.
10. CROSSOVER PROCEDURE.sh.
11. DOUBLE-BLIND PROCEDURE.sh.
12. RANDOMIZED CONTROLLED TRIAL.sh.
13. SINGLE BLIND PROCEDURE.sh.
14. or/1-13
15. ANIMAL/ or NONHUMAN/ or ANIMAL EXPERIMENT/
16. HUMAN/
17. 16 and 15
18. 15 not 17
19. 14 not 18

**ISI Web of Science and ISI Conference Proceedings Search Strategy**

TS=((pulp cap* OR pulpectom* OR pulpotom* OR endodontic* OR root canal therap* OR root canal treat*) AND (child* Or infant* OR primary OR deciduous OR milk Or baby OR temporary OR natal))

AND

TS=(random* or trial* or placebo* or group*)

**Open Grey Search Strategy**

A series of keyword searches was performed:

pulp and cap* and dental

pulp and cap* and teeth

pulp and cap* and tooth

pulpectom*

pulpotom*

endodontic* and child*

endodontic* and primary

root canal and child*

root canal and primary
