# Supplementary material for: Development of a Core Set of Outcomes for Randomized Controlled Trials with Multiple Outcomes – Example of Pulp Treatments of Primary Teeth for Extensive Decay in Children
Source: PLoS One. 2013 Jan 3;8(1):e51908. doi: 10.1371/journal.pone.0051908 (PMC3536772; doi:10.1371/journal.pone.0051908)
Supplement: Text S2 — Included and excluded studies, and reasons for exclusion. (DOC) [file pone.0051908.s009.doc]

**Text S2. List of included and excluded studies, and reasons for exclusion**

1. List of included studies
2. Aeinehchi M, Dadvand S, Fayazi S, Bayat-Movahed S. Randomized controlled trial of mineral trioxide aggregate and formocresol for pulpotomy in primary molar teeth. International Endodontic Journal. 2007;40(4):261-7.
3. Agamy HA, Bakry NS, Mounir MM, Avery DR. Comparison of mineral trioxide aggregate and formocresol as pulp-capping agents in pulpotomized primary teeth. Pediatric Dentistry. 2004;26(4):302-9.
4. Alacam A. Long term effects of primary teeth pulpotomies with formocresol, glutaraldehyde-calcium hydroxide and glutaraldehyde-zinc oxide eugenol on succudaneous teeth. Journal of Pedodontics. 1989;13(4):307-13. AND Alacam A. Pulpal tissue changes following pulpotomies with formocresol, glutaraldehyde-calcium hydroxide, glutaraldehyde-zinc oxide eugenol pastes in primary teeth. Journal of Pedodontics. 1989;13(2):123-32.
5. Alacam A, Odabas ME, Tuzuner T, Sillelioglu H, Baygin O. Clinical and radiographic outcomes of calcium hydroxide and formocresol pulpotomies performed by dental students. Oral Surgery Oral Medicine Oral Pathology Oral Radiology & Endodontics. 2009;108(5):e127-33.
6. Aminabadi NA, Farahani RM, Oskouei SG. Formocresol versus calcium hydroxide direct pulp capping of human primary molars: two year follow-up. Journal of Clinical Pediatric Dentistry. 2010;34(4):317-21.
7. Ansari G, Ranjpour M. Mineral trioxide aggregate and formocresol pulpotomy of primary teeth: a 2-year follow-up. International Endodontic Journal. 2010;43(5):413-8.
8. Bahrololoomi Z, Moeintaghavi A, Emtiazi M, Hosseini G. Clinical and radiographic comparison of primary molars after formocresol and electrosurgical pulpotomy: a randomized clinical trial. Indian Journal of Dental Research. 2008;19(3):219-23.
9. Casas MJ, Kenny DJ, Johnston DH, Judd PL. Long-term outcomes of primary molar ferric sulfate pulpotomy and root canal therapy. Pediatric Dentistry. 2004;26(1):44-8. AND Casas MJ, Layug MA, Kenny DJ, Johnston DH, Judd PL. Two-year outcomes of primary molar ferric sulfate pulpotomy and root canal therapy. Pediatric Dentistry. 2003;25(2):97-102.
10. Coser RM, Gondim JO, Aparecida Giro EM. Evaluation of 2 endodontic techniques used to treat human primary molars with furcation radiolucency area: A 48-month radiographic study. Quintessence International. 2008;39(7):549-57.
11. Dean JA, Mack RB, Fulkerson BT, Sanders BJ. Comparison of electrosurgical and formocresol pulpotomy procedures in children. International Journal of Paediatric Dentistry. 2002;12(3):177-82.
12. Demir T, Cehreli ZC. Clinical and radiographic evaluation of adhesive pulp capping in primary molars following hemostasis with 1.25% sodium hypochlorite: 2-year results. American Journal of Dentistry. 2007;20(3):182-8.
13. Doyle TL, Casas MJ, Kenny DJ, Judd PL. Mineral trioxide aggregate produces superior outcomes in vital primary molar pulpotomy. Pediatric Dentistry. 2010;32(1):41-7.
14. Eidelman E, Holan G, Fuks AB. Mineral trioxide aggregate vs. formocresol in pulpotomized primary molars: a preliminary report. Pediatric Dentistry. 2001;23(1):15-8.
15. Erdem AP, Guven Y, Balli B, Ilhan B, Sepet E, Ulukapi I, et al. Success rates of mineral trioxide aggregate, ferric sulfate, and formocresol pulpotomies: a 24-month study. Pediatric Dentistry. 2011;33(2):165-70.
16. Farsi N, Alamoudi N, Balto K, Mushayt A. Success of mineral trioxide aggregate in pulpotomized primary molars. Journal of Clinical Pediatric Dentistry. 2005;29(4):307-11.
17. Fei AL, Udin RD, Johnson R. A clinical study of ferric sulfate as a pulpotomy agent in primary teeth. Pediatric Dentistry. 1991;13(6):327-32.
18. Fishman SA, Udin RD, Good DL, Rodef F. Success of electrofulguration pulpotomies covered by zinc oxide and eugenol or calcium hydroxide: a clinical study. Pediatric Dentistry. 1996;18(5):385-90.
19. Fuks AB, Holan G, Davis JM, Eidelman E. Ferric sulfate versus dilute formocresol in pulpotomized primary molars: long-term follow up. Pediatric Dentistry. 1997;19(5):327-30.
20. Garrocho-Rangel A, Flores H, Silva-Herzog D, Hernandez-Sierra F, Mandeville P, Pozos-Guillen AJ. Efficacy of EMD versus calcium hydroxide in direct pulp capping of primary molars: a randomized controlled clinical trial. Oral Surgery Oral Medicine Oral Pathology Oral Radiology & Endodontics. 2009;107(5):733-8.
21. Holan G, Eidelman E, Fuks AB. Long-term evaluation of pulpotomy in primary molars using mineral trioxide aggregate or formocresol. Pediatric Dentistry. 2005;27(2):129-36.
22. Huth KC, Paschos E, Hajek-Al-Khatar N, Hollweck R, Crispin A, Hickel R, et al. Effectiveness of 4 pulpotomy techniques--randomized controlled trial. Journal of Dental Research. 2005;84(12):1144-8.
23. Ibricevic H, al-Jame Q. Ferric sulfate as pulpotomy agent in primary teeth: twenty month clinical follow-up. Journal of Clinical Pediatric Dentistry. 2000;24(4):269-72. Ferric sulphate and formocresol in pulpotomy of primary molars: long term follow-up study. European Journal of Paediatric Dentistry. 2003;4(1):28-32
24. Malekafzali B, Shekarchi F, Asgary S. Treatment outcomes of pulpotomy in primary molars using two endodontic biomaterials. A 2-year randomised clinical trial. European Journal of Paediatric Dentistry. 2011;12(3):189-93.
25. Markovic D, Zivojinovic V, Vucetic M. Evaluation of three pulpotomy medicaments in primary teeth. European Journal of Paediatric Dentistry. 2005;6(3):133-8.
26. Moretti AB, Sakai VT, Oliveira TM, Fornetti AP, Santos CF, Machado MA, et al. The effectiveness of mineral trioxide aggregate, calcium hydroxide and formocresol for pulpotomies in primary teeth. International Endodontic Journal. 2008;41(7):547-55.
27. Mortazavi M, Mesbahi M. Comparison of zinc oxide and eugenol, and Vitapex for root canal treatment of necrotic primary teeth. International Journal of Paediatric Dentistry. 2004;14(6):417-24.
28. Nadkarni U, Damle SG. Comparative evaluation of calcium hydroxide and zinc oxide eugenol as root canal filling materials for primary molars: a clinical and radiographic study. Journal of the Indian Society of Pedodontics & Preventive Dentistry. 2000;18(1):1-10.
29. Naik S, Hegde AH. Mineral trioxide aggregate as a pulpotomy agent in primary molars: an in vivo study. Journal of the Indian Society of Pedodontics & Preventive Dentistry. 2005;23(1):13-6.
30. Nakornchai S, Banditsing P, Visetratana N. Clinical evaluation of 3Mix and Vitapex as treatment options for pulpally involved primary molars. International Journal of Paediatric Dentistry. 2010;20(3):214-21.
31. Noorollahian H. Comparison of mineral trioxide aggregate and formocresol as pulp medicaments for pulpotomies in primary molars. British Dental Journal. 2008;204(11):E20.
32. Ozalp N, Saroglu I, Sonmez H. Evaluation of various root canal filling materials in primary molar pulpectomies: an in vivo study. American Journal of Dentistry. 2005;18(6):347-50.
33. Pinky C, Shashibhushan KK, Subbareddy VV. Endodontic treatment of necrosed primary teeth using two different combinations of antibacterial drugs: an in vivo study. Journal of the Indian Society of Pedodontics & Preventive Dentistry. 2011;29(2):121-7.
34. Prabhakar AR, Sridevi E, Raju OS, Satish V. Endodontic treatment of primary teeth using combination of antibacterial drugs: an in vivo study. Journal of Indian Society of Pedodontics and Preventive Dentistry. 2008;26(5):5-10.
35. Ramar K, Mungara J. Clinical and radiographic evaluation of pulpectomies using three root canal filling materials: an in-vivo study. Journal of the Indian Society of Pedodontics & Preventive Dentistry. 2010;28(1):25-9.
36. Sabbarini J, Mohamed A, Wahba N, El-Meligy O, Dean J. Comparison of enamel matrix derivative versus formocresol as pulpotomy agents in the primary dentition. Journal of Endodontics. 2008;34(3):284-7.
37. Sakai VT, Moretti AB, Oliveira TM, Fornetti AP, Santos CF, Machado MA, et al. Pulpotomy of human primary molars with MTA and Portland cement: a randomised controlled trial. British Dental Journal. 2009;207(3):E5; discussion 128-9.
38. Saltzman B, Sigal M, Clokie C, Rukavina J, Titley K, Kulkarni GV. Assessment of a novel alternative to conventional formocresol-zinc oxide eugenol pulpotomy for the treatment of pulpally involved human primary teeth: diode laser-mineral trioxide aggregate pulpotomy. International Journal of Paediatric Dentistry. 2005;15(6):437-47.
39. Shumayrikh NM, Adenubi JO. Clinical evaluation of glutaraldehyde with calcium hydroxide and glutaraldehyde with zinc oxide eugenol in pulpotomy of primary molars. Endodontics & Dental Traumatology. 1999;15(6):259-64.
40. Sonmez D, Sari S, Cetinbas T. A Comparison of four pulpotomy techniques in primary molars: a long-term follow-up. Journal of Endodontics. 2008;34(8):950-5.
41. Subramaniam P, Gilhotra K. Endoflas, zinc oxide eugenol and metapex as root canal filling materials in primary molars--a comparative clinical study. Journal of Clinical Pediatric Dentistry. 2011;35(4):365-9.
42. Subramaniam P, Konde S, Mathew S, Sugnani S. Mineral trioxide aggregate as pulp capping agent for primary teeth pulpotomy: 2 year follow up study. Journal of Clinical Pediatric Dentistry. 2009;33(4):311-4.
43. Trairatvorakul C, Chunlasikaiwan S. Success of pulpectomy with zinc oxide-eugenol vs calcium hydroxide/iodoform paste in primary molars: a clinical study. Pediatric Dentistry. 2008;30(4):303-8.
44. Tuna D, Olmez A. Clinical long-term evaluation of MTA as a direct pulp capping material in primary teeth. International Endodontic Journal. 2008;41(4):273-8.
45. Vargas KG, Packham B, Lowman D. Preliminary evaluation of sodium hypochlorite for pulpotomies in primary molars. Pediatric Dentistry. 2006;28(6):511-7.
46. Waterhouse PJ, Nunn JH, Whitworth JM. An investigation of the relative efficacy of Buckley's Formocresol and calcium hydroxide in primary molar vital pulp therapy. British Dental Journal. 2000;188(1):32-6. AND Waterhouse PJ, Nunn JH, Whitworth JM. Prostaglandin E2 and treatment outcome in pulp therapy of primary molars with carious exposures. International Journal of Paediatric Dentistry. 2002;12(2):116-23.
47. Zealand CM, Briskie DM, Botero TM, Boynton JR, Hu JC. Comparing gray mineral trioxide aggregate and diluted formocresol in pulpotomized human primary molars. Pediatric Dentistry. 2010;32(5):393-9.
48. Zurn D, Seale NS. Light-cured calcium hydroxide vs formocresol in human primary molar pulpotomies: a randomized controlled trial. Pediatric Dentistry. 2008;30(1):34-41.
49. Excluded studies and reasons for exclusion

| Excluded studies | Reason for exclusion |
| --- | --- |
| Evidence-Based Review of Clinical Studies on Surgery. Journal of Endodontics. 2009;35(8):1094-110. | Review |
| Evidence-Based Review of Clinical Studies on Pulpotomy. Journal of Endodontics. 2009;35(8):1116-20. | Review |
| Evidence-Based Review of Clinical Studies on Pulpectomy. Journal of Endodontics. 2009;35(8):1121-2. | Review |
| Evidence-Based Review of Clinical Studies on Pharmacology (non-anesthetic studies). Journal of Endodontics. 2009;35(8):1123-9. | Review |
| Evidenced-Based Review of Clinical Studies on Non-Surgical Endodontic Treatment. Journal of Endodontics. 2009;35(8):1139-44. | Review |
| Evidenced-Based Review of Clinical Studies on Endodontic Microflora. Journal of Endodontics. 2009;35(8):1145-6. | Review |
| Evidenced-Based Review of Clinical Studies on Indirect Pulp Capping. Journal of Endodontics. 2009;35(8):1147-51. | Review |
| Evidenced-Based Review of Clinical Studies on Direct Pulp Capping. Journal of Endodontics. 2009;35(8):1152-4. | Review |
| Evidenced-Based Review of Clinical Studies on Endodontic Diagnosis. Journal of Endodontics. 2009;35(8):1155-7. | Review |
| Aartman IH, de Jongh A, Makkes PC, Hoogstraten J. Treatment modalities in a dental fear clinic and the relation with general psychopathology and oral health variables. British Dental Journal. 1999;186(9):467-71. | Psychology |
| Abdel-Aziz A, Abdella A. Clinical and histological evaluation of three antiseptics used in abscessed primary molars [abstract]. Pediatric Dentistry. 1999;21(5):115. | No RCT |
| Abo-Hamar SE, Federlin M, Hiller KA, Friedl KH, Schmalz G. Effect of temporary cements on the bond strength of ceramic luted to dentin. Dental Materials. 2005;21(9):794-803. | Permanent teeth |
| Addo ME, Parekh S, Moles DR, Roberts GJ. Knowledge of dental trauma first aid (DTFA): The example of avulsed incisors in casualty departments and schools in London. British Dental Journal. 2007;202(10):E27. | Surgery |
| Adib V, Spratt D, Ng Y, Gulabivala K. Cultivable microbial flora associated with persistent periapical disease and coronal leakage after root canal treatment: a preliminary study. International Endodontic Journal. 2004;37(8):542-51. | In vitro study |
| Akdeniz BG, Koparal E, Sen BH, Ates M, Denizci AA. Prevalence of Candida albicans in oral cavities and root canals of children. Journal of Dentistry for Children. 2002;69(3):289-92. | Prevalence study |
| Akgun OM, Altun C, Guven G. Use of triple antibiotic paste as a disinfectant for a traumatized immature tooth with a periapical lesion: a case report. Oral Surgery Oral Medicine Oral Pathology Oral Radiology & Endodontics. 2009;108(2):e62-5. | Permanent teeth |
| Akkocaoglu M, Uysal S, Tekdemir I, Akca K, Cehreli MC. Implant design and intraosseous stability of immediately placed implants: a human cadaver study. Clinical Oral Implants Research. 2005;16(2):202-9. | Implantology |
| Aktoren O, GenÁay K. A two year clinical-radiographic follow-up of the pulpotomies in primary molars [abstract]. Journal of Dental Research. 2000;79(Spec Iss [Abstracts]):543, Abstract no: 3193. | Abstract only |
| Al Amoudi N, Feda M, Sharaf A, Hanno A, Farsi N. Assessment of the anesthetic effectiveness of anterior and middle superior alveolar injection using a computerized device versus traditional technique in children. Journal of Clinical Pediatric Dentistry. 2008;33(2):97-102. | Anesthesia |
| Al-Asfour A, Andersson L. The effect of a leaflet given to parents for first aid measures after tooth avulsion. Dental Traumatology. 2008;24(5):515-21. | Surgery |
| Al-Awadhi S, Spears R, Gutmann JL, Opperman LA. Cultured primary osteoblast viability and apoptosis in the presence of root canal sealers. Journal of Endodontics. 2004;30(7):527-33. | In vitro study |
| Al-Hezaimi K, Al-Tayar BA, Bajuaifer YS, Salameh Z, Al-Fouzan K, Tay FR. A Hybrid Approach to Direct Pulp Capping by Using Emdogain with a Capping Material. Journal of Endodontics. 2011;37(5):667-72. | Animal study |
| Al-Kahtani AM, Al-Fawaz H, Al-Sarhan M, Al-Ali K. Fracture resistance of teeth obturated with RealSeal using two different chelating agents: an in vitro study. Journal of Contemporary Dental Practice [Electronic Resource]. 2010;11(1):E025-32. | In vitro study |
| Al-Madi EM. Prevalence of pulpally involved permanent teeth in Saudi schoolchildren. International Dental Journal. 2004;54(4):206-10. | Permanent teeth |
| Alacam A. The effect of various irrigants on the adaptation of paste filling in primary teeth. Journal of Clinical Pediatric Dentistry. 1992;16(4):243-6. | In vitro study |
| Alacam T, Tinaz AC. Interappointment emergencies in teeth with necrotic pulps. Journal of Endodontics. 2002;28(5):375-7. | Permanent teeth |
| Alani A, Knowles JC, Chrzanowski W, Ng YL, Gulabivala K. Ion release characteristics, precipitate formation and sealing ability of a phosphate glass-polycaprolactone-based composite for use as a root canal obturation material. Dental Materials. 2009;25(3):400-10. | In vitro study |
| Ali Kalhoro F, Mirza AJ. A study of flare-ups following single-visit root canal treatment in endodontic patients. Journal of the College of Physicians and Surgeons Pakistan. 2009;19(7):410-2. | Permanent teeth |
| Alio Sanz JJ, Barberia Leache E, Moreno Gonzalez PJ. [Epidemiologic study of the prevalence of dental caries in patients with Down syndrome]. [Spanish]. Revista de Sanidad e Higiene Publica. 1989;63(1-2):63-70. | Epidemiologic study |
| Allen EP, Bayne SC, Cronin RJ, Donovan TE, Kois JC, Summitt JB. Annual review of selected dental literature: Report of the Committee on Scientific Investigation of the American Academy of Restorative Dentistry. Journal of Prosthetic Dentistry. 2004;92(1):39-71. | Review |
| Altonen M, Mattila K. Follow-up study of apicoectomized molars. International Journal of Oral Surgery. 1976;5(1):33-40. | Surgery |
| Alves dos Santos MP, Luiz RR, Maia LC. Randomised trial of resin-based restorations in Class I and Class II beveled preparations in primary molars: 48-month results. Journal of dentistry. 2010;38(6):451-9. | Restorative dentistry |
| Alves Dos Santos MP, Passos M, Luiz RR, Maia LC. A randomized trial of resin-based restorations in Class I and Class II beveled preparations in primary molars: 24-month results. Journal of the American Dental Association. 2009;140(2):156-66. | Restorative dentistry |
| Alves FB, Vieira Rde S. Effects of eugenol and non-eugenol endodontic fillers on short post retention, in primary anterior teeth: an in vitro study. Journal of Clinical Pediatric Dentistry. 2005;29(3):211-4. | In vitro study |
| Ambrosino DM, Bolon D, Collard H, Vanetten R, Kanchana MV, Finberg RW. EFFECT OF HAEMOPHILUS-INFLUENZAE POLYSACCHARIDE OUTER-MEMBRANE PROTEIN COMPLEX CONJUGATE VACCINE ON MACROPHAGES. Journal of Immunology. 1992;149(12):3978-83. | Immunology |
| Aminabadi NA, Farahani RM, Gajan EB. A clinical study of formocresol pulpotomy versus root canal therapy of vital primary incisors. Journal of Clinical Pediatric Dentistry. 2008;32(3):211-4. | Primary incisors |
| Andersen MG, Beck-Nielsen SS, Haubek D, Hintze H, Gjorup H, Poulsen S. Periapical and endodontic status of permanent teeth in patients with hypophosphatemic rickets. Journal of Oral Rehabilitation. 2012;39(2):144-50. | Permanent teeth |
| Anderson RW, Powell BJ, Pashley DH. Microleakage of IRM used to restore endodontic access preparations. Endodontics & Dental Traumatology. 1990;6(4):137-41. | In vitro study |
| Andersson-Wenckert IE, van Dijken JW, Horstedt P. Modified Class II open sandwich restorations: evaluation of interfacial adaptation and influence of different restorative techniques. European journal of oral sciences. 2002;110(3):270-5. | Restorative dentistry |
| Andrade AKM, Duarte RM, Silva F, Batista AUD, Lima KC, Pontual MLA, et al. Efficacy of composites filled with nanoparticles in permanent molars: Six-month results. General Dentistry. 2010;58(5):e190-e5. | Permanent teeth |
| Angwaravong O, Panitvisai P. Accuracy of an electronic apex locator in primary teeth with root resorption. International Endodontic Journal. 2009;42(2):115-21. | Apex locator study |
| Ansari G, Vahid Golpaygani M, Chitsazan I, Fekrazad R. Clinical and radiographic evaluation of Diode laser pulpotomy on human primary teeth: A preliminary study. Lasers in Medical Science Conference: World Federation for Laser Dentistry (WFLD) Congress Hong Kong Hong Kong Conference Start: 20080728 Conference End: 20080730. 2009;24(3):470-1. | Abstract only |
| Anusavice KJ. Does ART have a place in preservative dentistry? Community Dentistry and Oral Epidemiology. 1999;27(6):442-8. | Epidemiologic study |
| Aquilino SA, Caplan DJ. Relationship between crown placement and the survival of endodontically treated teeth. Journal of Prosthetic Dentistry. 2002;87(3):256-63. | Permanent teeth |
| Arrastia AM, Wilder-Smith P, Berns MW. Thermal effects of CO2 laser on the pulpal chamber and enamel of human primary teeth: an in vitro investigation. Lasers in Surgery & Medicine. 1995;16(4):343-50. | In vitro study |
| Arweiler NB, Auschill TM, Reich E. Does pretreatment of cavities effectively promote good marginal adaptation of glass-ionomer cements? The journal of adhesive dentistry. 2000;2(4):289-95. | Permanent teeth |
| Asgary S, Eghbal MJ. A clinical trial of pulpotomy vs. root canal therapy of mature molars.[Retraction in Giannobile WV. J Dent Res. 2011 Sep;90(9):1145; PMID: 21844531]. Journal of Dental Research. 2010;89(10):1080-5. | Permanent teeth |
| Asgary S, Eghbal MJ. The effect of pulpotomy using a calcium-enriched mixture cement versus one-visit root canal therapy on postoperative pain relief in irreversible pulpitis: a randomized clinical trial. Odontology/The Society of the Nippon Dental University. 2010;98(2):126-33. | Permanent teeth |
| Ashkenazi M, Blumer S, Eli I. Post-operative pain and use of analgesic agents in children following intrasulcular anaesthesia and various operative procedures. British Dental Journal. 2007;202(5):E13; discussion 276-7. | Anesthesia |
| Ashwin R, Arathi R. Comparative evaluation for microleakage between Fuji-VII glass ionomer cement and light-cured unfilled resin: A combined in vivo in vitro study. Journal of Indian Society of Pedodontics and Preventive Dentistry. 2007;25(2):86-7. | In vitro study |
| Asselin ME, Fortin D, Sitbon Y, Rompre PH. Marginal microleakage of a sealant applied to permanent enamel: evaluation of 3 application protocols. Pediatric dentistry. 2008;30(1):29-33. | In vitro study |
| Atash R, Bottenberg P, Petein M, Vanden Abbeele A. In vitro evaluation of the marginal seal of four restoration materials on deciduous molars. Bulletin du Groupement international pour la recherche scientifique en stomatologie & odontologie. 2003;45(1):34-41. | In vitro study |
| Atieh M. Stainless steel crown versus modified open-sandwich restorations for primary molars: a 2-year randomized clinical trial. International Journal of Paediatric Dentistry. 2008;18(5):325-32. | Restorative dentistry |
| Attin T, Opatowski A, Meyer C, Zingg-Meyer B, Buchalla W, Monting JS. Three-year follow up assessment of Class II restorations in primary molars with a polyacid-modified composite resin and a hybrid composite. American Journal of Dentistry. 2001;14(3):148-52. | Restorative dentistry |
| Ayhan H, Alacam A, Olmez A. Apical microleakage of primary teeth root canal filling materials by clearing technique. Journal of Clinical Pediatric Dentistry. 1996;20(2):113-7. | In vitro study |
| Ayrton Ode T, Benfatti SV, Andrioni JN. [Clinical and morphological study of a therapeutic agent with formaldehyde base for the preservation of the deciduous teeth]. [Spanish]. Cooperador Dental. 1969;35(1):16-21. | No RCT |
| Azar MR, Mokhtare M. Rotary Mtwo system versus manual K-file instruments: efficacy in preparing primary and permanent molar root canals. Indian Journal of Dental Research. 2011;22(2):363. | Biomaterials not compared |
| Backstrom M, Kolar MC, Htun M. Characterisation of fines from unbleached kraft pulps and their impact on sheet properties. Holzforschung. 2008;62(5):546-52. | Permanent teeth |
| Badzian-Kobos K, Walczak A. [Clinical evaluation of the results of treatment of live dental pulp with Ledermix and Calxyl in some pulp diseases in children]. [Polish]. Czasopismo Stomatologiczne. 1967;20(11):1139-43. | No RCT |
| Badzian-Kobos K, Walczak A. [Assessment of treatment of pulpitis in deciduous molars of children aged 3-7 with Rezoform paste]. [Polish]. Czasopismo Stomatologiczne. 1970;23(8):925-7. | Translation problem |
| Badzian-Kobos K, Walczak A, Wochna-Sobanska M. [Evaluation of results of treatment of infected root canals in permanent teeth in children with Rezoform paste and N 2 paste]. [Polish]. Czasopismo Stomatologiczne. 1973;26(3):237-41. | Permanent teeth |
| Badzian-Kobos K, Wochna-Sobanska M, Szosland E. [Evaluation of treatment of pulpitis in decidous teeth formocresol and N2 paste]. [Polish]. Czasopismo Stomatologiczne. 1976;29(1):15-8. | No possibility of translation |
| Baghdadi ZD. Evaluation of electronic dental anesthesia in children. Oral Surgery, Oral Medicine, Oral Pathology, Oral Radiology, and Endodontics. 1999;88(4):418-23. | Anesthesia |
| Bagis B, Atilla P, Cakar N, Hasanreisoglu U. An immunohistochemical evaluation of cell adhesion molecules in human dental pulp after tooth preparation and application of temporary luting cements. Oral Surgery Oral Medicine Oral Pathology Oral Radiology & Endodontics. 2009;107(1):137-44. | In vitro study |
| Balto H. An assessment of microbial coronal leakage of temporary filling materials in endodontically treated teeth. Journal of Endodontics. 2002;28(11):762-4. | In vitro study |
| Balto H, Al-Nazhan S, Al-Mansour K, Al-Otaibi M, Siddiqu Y. Microbial leakage of Cavit, IRM, and Temp Bond in post-prepared root canals using two methods of gutta-percha removal: an in vitro study. Journal of Contemporary Dental Practice [Electronic Resource]. 2005;6(3):53-61. | In vitro study |
| Bansal S, Tewari S. Ex vivo evaluation of dye penetration associated with various dentine bonding agents in conjunction with different irrigation solutions used within the pulp chamber. International endodontic journal. 2008;41(11):950-7. | Ex vivo study |
| Barberia E, Arenas M, Gomez B, Saavedra-Ontiveros D. An audit of paediatric dental treatments carried out under general anaesthesia in a sample of Spanish patients. Community Dental Health. 2007;24(1):55-8. | Epidemiologic study |
| Barcelos R, Santos MPA, Primo LG, Luiz RR, Maia LC. ZOE Paste Pulpectomies Outcome in Primary Teeth: A Systematic Review. Journal of Clinical Pediatric Dentistry. 2011;35(3):241-8. | Review |
| Barker BC, Payne M, Warby HA. Two case reports: successful treatment of carious exposures with Ledermix. Australian Dental Journal. 1972;17(2):144-6. | Permanent teeth |
| Barrieshi-Nusair KM, Al-Omari MA, Al-Hiyasat AS. Radiographic technical quality of root canal treatment performed by dental students at the Dental Teaching Center in Jordan. Journal of Dentistry. 2004;32(4):301-7. | Permanent teeth |
| Barrieshi-Nusair KM, Qudeimat MA. A prospective clinical study of mineral trioxide aggregate for partial pulpotomy in cariously exposed permanent teeth. Journal of Endodontics. 2006;32(8):731-5. | Permanent teeth |
| Barthel CR, Strobach A, Briedigkeit H, Gobel UB, Roulet JF. Leakage in roots coronally sealed with different temporary fillings. Journal of Endodontics. 1999;25(11):731-4. | In vitro study |
| Barthel CR, Zimmer S, West G, Roulet JF. Bacterial leakage in obturated root canals following the use of different intracanal medicaments. Endodontics & Dental Traumatology. 2000;16(6):282-6. | In vitro study |
| Barthel CR, Zimmer S, Wussogk R, Roulet JF. Long-Term bacterial leakage along obturated roots restored with temporary and adhesive fillings. Journal of Endodontics. 2001;27(9):559-62. | In vitro study |
| Barthelemy Y, Colombel M, Gasman D, Patard JJ, Chopin D, Abbou CC. [Antiandrogen withdrawal syndrome in the hormonal treatment of metastatic prostatic cancer in hormonal escape]. [French]. Progres en Urologie. 1996;6(1):93-7. | Irrelevant |
| Baseggio W, Naufel FS, Davidoff DC, Nahsan FP, Flury S, Rodrigues JA. Caries-preventive efficacy and retention of a resin-modified glass ionomer cement and a resin-based fissure sealant: a 3-year split-mouth randomised clinical trial. Oral Health and Preventive Dentistry. 2010;8(3):261-8. | Prevention |
| Basrani B, Santos JM, Tjaderhane L, Grad H, Gorduysus O, Huang J, et al. Substantive antimicrobial activity in chlorhexidine-treated human root dentin. Oral Surgery, Oral Medicine, Oral Pathology, Oral Radiology, and Endodontics. 2002;94(2):240-5. | In vitro study |
| Baumgartner JC, Reid DE, Pickett AB. Human pulpal reaction to the modified McInnes bleaching technique. Journal of Endodontics. 1983;9(12):527-9. | Bleaching |
| Bawazir OA, Salama FS. Clinical evaluation of root canal obturation methods in primary teeth. Pediatric Dentistry. 2006;28(1):39-47. | Biomaterials not compared |
| Bawazir OA, Salama FS. Apical microleakage of primary teeth root canal filling materials. Journal of Dentistry for Children (Chicago, Ill. 2007). 74(1):46-51. | In vitro study |
| Beach CW, Calhoun JC, Bramwell JD, Hutter JW, Miller GA. Clinical evaluation of bacterial leakage of endodontic temporary filling materials. Journal of Endodontics. 1996;22(9):459-62. | In vitro study |
| Beaver HA, Kopel HM, Sabes WR. The effect of zinc oxide-eugenol cement on a formocresolized pulp. Journal of Dentistry-Child. 1966;33(6):381-96. | No RCT |
| Beck-Nielsen SS, Brusgaard K, Rasmussen LM, Brixen K, Brock-Jacobsen B, Poulsen MR, et al. Phenotype Presentation of Hypophosphatemic Rickets in Adults. Calcified Tissue International. 2010;87(2):108-19. | Irrelevant |
| Beltrame AP, Triches TC, Sartori N, Bolan M. Electronic determination of root canal working length in primary molar teeth: an in vivo and ex vivo study. International Endodontic Journal. 2011;44(5):402-6. | Apex locator study |
| Bergoli AD, Primosch RE, de Araujo FB, Ardenghi TM, Casagrande L. Pulp Therapy in Primary Teeth - Profile of teaching in Brazilian Dental Schools. Journal of Clinical Pediatric Dentistry. 2010;35(2):191-5. | Epidemiologic study |
| Berkman MD, Cucolo FA, Levin MP, Brunelle LJ. Pulpal response to isobutyl cyanoacrylate in human teeth. Journal of the American Dental Association. 1971;83(1):140-5. | In vitro study |
| Berrebi J, Heysselaer D, Nyssen-Behets C, Rocca JP, Mahler P, Limme M, et al. Clinical treatment of exposed pulp by Direct Capping/Pulpotomy on primary and permanent immature teeth. Lasers in Medical Science Conference: World Federation for Laser Dentistry (WFLD) Congress Hong Kong Hong Kong Conference Start: 20080728 Conference End: 20080730. 2009;24(3):472. | Abstract only |
| Beslot-Neveu A, Bonte E, Baune B, Serreau R, Aissat F, Quinquis L, et al. Mineral trioxyde aggregate versus calcium hydroxide in apexification of non vital immature teeth: study protocol for a randomized controlled trial. Trials. 2011;12:Article no: 174. | Permanent teeth |
| Biggs JE, Yates JM, Loescher AR, Clayton NM, Boissonade FM, Robinson PP. Changes in vanilloid receptor 1 (TRPV1) expression following lingual nerve injury. European Journal of Pain. 2007;11(2):192-201. | Irrelevant |
| Birch S, Gafni A, Markham B, Marriott M, Lewis D, Main P. Health years equivalents as a measurement of preferences for dental interventions. Community dental health. 1998;15(4):233-42. | Epidemiologic study |
| Bizhang M, Chun YH, Heisrath D, Purucker P, Singh P, Kersten T, et al. Microbiota of exposed root surfaces after fluoride, chlorhexidine, and periodontal maintenance therapy: a 3-year evaluation. Journal of Periodontology. 2007;78(8):1580-9. | In vitro study |
| Bizhang M, Seemann R, Rˆmhild G, Chun YH, Umland N, Lang H, et al. Effect of a 40% chlorhexidine varnish on demineralization of dentin surfaces in situ. American Journal of Dentistry. 2007;20(3):193-7. | In vitro study |
| Bjorndal L, Larsen T, Thylstrup A. A clinical and microbiological study of deep carious lesions during stepwise excavation using long treatment intervals. Caries research. 1997;31(6):411-7. | In vitro study |
| Bjorndal L, Reit C. The annual frequency of root fillings, tooth extractions and pulp-related procedures in Danish adults during 1977-2003. International Endodontic Journal. 2004;37(11):782-8. | Epidemiologic study |
| Bjorndal L, Reit C, Bruun G, Markvart M, Kjaeldgaard M, Nasman P, et al. Treatment of deep caries lesions in adults: randomized clinical trials comparing stepwise vs. direct complete excavation, and direct pulp capping vs. partial pulpotomy. European Journal of Oral Sciences. 2010;118(3):290-7. | Adults |
| Blome B, Braun A, Sobarzo V, Jepsen S. Molecular identification and quantification of bacteria from endodontic infections using real-time polymerase chain reaction. Oral Microbiology & Immunology. 2008;23(5):384-90. | In vitro study |
| Blum JY, Peli JF, Abadie MJM. Effects of the Nd : YAP laser on coronal restorative materials: Implications for endodontic retreatment. Journal of Endodontics. 2000;26(10):588-92. | Permanent teeth |
| Boer FAC, Percinoto C, Ferelle A, Cunha RF. Immediate reimplantation of primary teeth: a histological study in dogs. Dental Traumatology. 2008;24(3):337-42. | Animal study |
| Boeve CM, Dermaut LR. [Radiographic follow-up of primary molars following sclerosing amputation]. [Dutch]. Revue Belge de Medecine Dentaire. 1982;37(1):9-15. | Biomaterials not compared |
| Boggs DC. Simple technique for treating non-vital deciduous teeth--a study. Northwest Dentistry. 1969;48(2):102-4. | No RCT |
| Bolin AK, Bolin A, Alfredsson L. Children's dental health in Europe. Caries treatment need in 5- and 12-year-old children from eight EU countries. Acta Odontologica Scandinavica. 1996;54(6):355-61. | Epidemiologic study |
| Bolla M, Muller-Bolla M, Borg C, Lupi-Pegurier L, Laplanche O, Leforestier E. Root canal posts for the restoration of root filled teeth. Cochrane database of systematic reviews (Online). 2007(1):CD004623. | Review |
| Bonanato K, Paiva SM, Pordeus IA, Ramos-Jorge ML, Barbabela D, Allison PJ. Relationship between mothers' sense of coherence and oral health status of preschool children. Caries Research. 2009;43(2):103-9. | Psychological study |
| Bonecker M, Mantesso A, de Araujo NS, Araujo VC. Expression of proteins in the extracellular matrix of pulp tissue in human primary teeth during physiologic root resorption. Quintessence International. 2009;40(7):553-8. | In vitro study |
| Bonifacio CC, van Amerongen WE, Meschini TG, Raggio DP, Bonecker M. Flowable glass ionomer cement as a liner: improving marginal adaptation of atraumatic restorative treatment restorations. Journal of dentistry for children (Chicago, Ill. 2010). 77(1):12-6. | Restorative dentistry |
| Borba de Araujo F, Eduardo Nor J, Thomazi TH. [Dilute formocresol. An alternative in pulp therapy for deciduous teeth]. [Review] [33 refs] [Portuguese]. Rgo. 1988;36(3):181-4. | Review |
| Borges AF, Bittar RA, Pascon FM, Sobrinho LC, Martin AA, Puppin Rontani RM. NaOCl effects on primary and permanent pulp chamber dentin. Journal of Dentistry. 2008;36(9):745-53. | In vitro study |
| Borges AFS, Correr GM, Sinhoreti MAC, Consani S, Sobrinho LC, Rontani RMP. Compressive strength recovery by composite onlays in primary teeth. Substrate treatment and luting agent effects. Journal of Dentistry. 2006;34(7):478-84. | Restorative dentistry |
| Borsatto MC, Corona SA, Chinelatti MA, Ramos RP, de Sa Rocha RA, Pecora JD, et al. Comparison of marginal microleakage of flowable composite restorations in primary molars prepared by high-speed carbide bur, Er:YAG laser, and air abrasion. Journal of dentistry for children (Chicago, Ill. 2006). 73(2):122-6. | In vitro study |
| Brannstrom M, Nyborg H, Stromberg T. Experiments with pulp capping. Oral Surgery, Oral Medicine, Oral Pathology. 1979;48(4):347-52. | Human and dog permanent teeth, in vitro |
| Brennan D, Spencer AJ, Szuster F. Service provision trends between 1983-84 and 1993-94 in Australian private general practice. Australian Dental Journal. 1998;43(5):331-6. | Epidemiologic study |
| Brennan DS, Spencer AJ, Szuster FS. Insurance status and provision of dental services in Australian private general practice. Community dentistry and oral epidemiology. 1997;25(6):423-8. | Epidemiologic study |
| Brennan MT, Kent ML, Fox PC, Norton HJ, Lockhart PB. The impact of oral disease and nonsurgical treatment on bacteremia in children. Journal of the American Dental Association. 2007;138(1):80-5. | Immunology |
| Bretz WA, Djahjah CA, Almeida RS, Villar do Valle E, Fonseca C, Valente I, et al. Effect of a chlorhexidine varnish on caries lesions. Oral Health. 1995;85(9):29-30. | In vitro study |
| Brukiene V, Aleksejuniene J, Balciuniene I. Dental treatment needs in Lithuanian adolescents. Stomatologija. 2005;7(1):11-5. | Epidemiology study |
| Brunton PA, Abidia R, Macfarlane TV, Wilson NH. An evaluation of powder-free gloves in general dental practice. Primary Dental Care. 2000;7(3):125-8. | Powder-free gloves |
| Buhrley LJ, Barrows MJ, BeGole EA, Wenckus CS. Effect of magnification on locating the MB2 canal in maxillary molars. Journal of Endodontics. 2002;28(4):324-7. | Permanent teeth |
| Buttke TM, Trope M. Effect of catalase supplementation in storage media for avulsed teeth. Dental Traumatology. 2003;19(2):103-8. | Surgery |
| Buyukgural B, Cehreli ZC. Effect of different adhesive protocols vs calcium hydroxide on primary tooth pulp with different remaining dentin thicknesses:24-month results. Clinical Oral Investigations. 2008;12(1):91-6. | Indirect pulp capping only |
| Calderon A. Prevention of apical periodontal ligament pain: a preliminary report of 100 vital pulp cases. J Endod. 1993;19(5):247-9. | Permanent teeth |
| Caliskan MK, Turkun M, Gokay N. Delayed replantation of avulsed mature teeth with calcium hydroxide treatment. Journal of Endodontics. 2000;26(8):472-6. | Surgery |
| Camargo CH, Siviero M, Camargo SE, de Oliveira SH, Carvalho CA, Valera MC. Topographical, diametral, and quantitative analysis of dentin tubules in the root canals of human and bovine teeth. Journal of Endodontics. 2007;33(4):422-6. | Anatomic study |
| Camp JH. Diagnosis dilemmas in vital pulp therapy: Treatment for the toothache is changing, especially in young, immature teeth. Journal of Endodontics. 2008;34(7):S6-S12. | Permanent teeth |
| Camp JH. Diagnosis dilemmas in vital pulp therapy: treatment for the toothache is changing, especially in young, immature teeth. [Review] [58 refs]. Pediatric Dentistry. 2008;30(3):197-205. | Permanent teeth |
| Cannon M, Cernigliaro J, Vieira A, Percinoto C, Jurado R. Effects Of Antibacterial Agents On Dental Pulps Of Monkeys Mechanically Exposed And Contaminated. Journal of Clinical Pediatric Dentistry. 2008;33(1):21-8. | Animal study |
| Cannon M, Wagner C, Thobaben JZ, Jurado R, Solt D. Early Response of Mechanically Exposed Dental Pulps of Swine to Antibacterial-Hemostatic Agents or Diode Laser Irradiation. Journal of Clinical Pediatric Dentistry. 2011;35(3):271-6. | Animal study |
| Cannon ML. A clinical study of the "open sandwich" technique in pediatric dental practice. Journal of dentistry for children (Chicago, Ill. 2003). 70(1):65-70. | Restorative dentistry |
| Cannon ML, Tylka JA, Sandrik J. A clinical study of adhesive amalgam in pediatric dental practice. Compendium of continuing education in dentistry (Jamesburg, N. 1999;J. : 1995). 20(4):331-4, 6, 8 passim; quiz 44. | Restorative dentistry |
| Carcao MD, Seary ME, Casas M, Winter L, Stain AM, Judd P. Dental disease in type 3 Von Willebrand disease: a neglected problem. Haemophilia. 2010;16(6):943-8. | General medical problem |
| Carrasco LD, Guerisoli DMZ, Pecora JD, Froner IC. Evaluation of dentin permeability after light activated internal dental bleaching. Dental Traumatology. 2007;23(1):30-4. | Bleaching |
| Carrasco LD, Pecora JD, Froner IC. In vitro assessment of dentinal permeability after the use of ultrasonic-activated irrigants in the pulp chamber before internal dental bleaching. Dental Traumatology. 2004;20(3):164-8. | In vitro study |
| Carrotte PV, Waterhouse PJ. A clinical guide to endodontics - update part 2. British Dental Journal. 2009;206(3):133-9. | Review |
| Carvalho TS, Sampaio FC, Diniz A, Bonecker M, Van Amerongen WE. Two years survival rate of Class II ART restorations in primary molars using two ways to avoid saliva contamination. International journal of paediatric dentistry / the British Paedodontic Society [and] the International Association of Dentistry for Children. 2010;20(6):419-25. | Restorative dentistry |
| Casagrande L, Bento LW, Dalpian DM, Garcia-Godoy F, de Araujo FB. Indirect pulp treatment in primary teeth: 4-year results. American Journal of Dentistry. 2010;23(1):34-8. | Indirect pulp capping only |
| Casagrande L, Bento LW, Rerin SO, Lucas Ede R, Dalpian DM, de Araujo FB. In vivo outcomes of indirect pulp treatment using a self-etching primer versus calcium hydroxide over the demineralized dentin in primary molars. Journal of Clinical Pediatric Dentistry. 2008;33(2):131-5. | Indirect pulp capping only |
| Casagrande L, Falster CA, Di Hipolito V, De Goes MF, Straffon LH, Nor JE, et al. Effect of adhesive restorations over incomplete dentin caries removal: 5-year follow-up study in primary teeth. Journal of Dentistry for Children (Chicago, Ill. 2009). 76(2):117-22. | Restorative dentistry |
| Casas M, Kenny D, Johnston D, Judd P, Layug M. Three-year prospective outcome study of ferric sulphate pulpotomies and root canal treatment in vital primary molars [abstract]. International journal of paediatric dentistry. 2003;13(Suppl 1):9. | Duplicate |
| Casas M, Kenny DJ, Layug MA. Two-year prospective outcome study of ferric sulfate pulpotomies and root canal treatment in vital primary molars [abstract]. Proceedings of the 6th Congress of the European Academy of Paediatric Dentistry; 2002; Dublin, Ireland. 2002. | Duplicate |
| Casas MJ, Kenny DJ, Johnston DH, Judd PL, Layug MA. Outcomes of vital primary incisor ferric sulfate pulpotomy and root canal therapy. Journal (Canadian Dental Association). 2004;70(1):34-8. | Duplicate |
| Cehreli SB, Yalcinkaya Z, Guven-Polat G, Cehreli ZC. Effect of ozone pretreatment on the microleakage of pit and fissure sealants. Journal of Clinical Pediatric Dentistry. 2010;35(2):187-90. | In vitro study |
| Cehreli ZC, Turgut M, Olmez S, Dagdeviren A, Atilla P. Short term human primary pulpal response after direct pulp capping with fourth-generation dentin adhesives. Journal of Clinical Pediatric Dentistry. 2000;25(1):65-71. | In vitro study |
| Chahverdiani B, Thadj-Bakhche A. [Ozone treatment in root canal therapy. Introduction and general discussion]. [French]. Acta Medica Iranica. 1976;19(3):192-200. | Permanent teeth |
| Chan AW, Wong TK, Cheung GS. Lay knowledge of physical education teachers about the emergency management of dental trauma in Hong Kong. Dental traumatology : official publication of International Association for Dental Traumatology. 2001;17(2):77-85. | Surgery |
| Chandra S, Chawla TN. Combination of triamcinolone acetonide and demethylchlortetracycline hydrochloride in the management of pulp hyperemia and acute serous pulpitis. Journal of the Indian Dental Association. 1974;46(7):263-70. | Permanent teeth |
| Chang YC, Lai CC, Yang SF, Chan Y, Hsieh YS. Stimulation of matrix metalloproteinases by black-pigmented Bacteroides in human pulp and periodontal ligament cell cultures. Journal of Endodontics. 2002;28(2):90-3. | In vitro study |
| Chaushu S, Shapira J, Heling I, Becker A. Emergency orthodontic treatment after the traumatic intrusive luxation of maxillary incisors. American Journal of Orthodontics and Dentofacial Orthopedics. 2004;126(2):162-72. | Orthodontics |
| Chawla HS. Apical closure in a nonvital permanent tooth using one Ca(OH)2 dressing. Journal of Dentistry for Children. 1986;53(1):44-7. | Permanent teeth |
| Chazel JC, Valcarcel J, Tramini P, Pelissier B, Mafart B. Coronal and apical lesions, environmental factors: study in a modern and an archeological population. Clinical Oral Investigations. 2005;9(3):197-202. | Epidemiologic study |
| Chedid JC, Pilipili C. [A 24 month evaluation of zinc oxide pulpotomy on primary canines]. [French]. Revue Belge de Medecine Dentaire. 2008;63(2):69-76. | Canines |
| Cheung GS, Chan TK. Long-term survival of primary root canal treatment carried out in a dental teaching hospital. International Endodontic Journal. 2003;36(2):117-28. | No RCT |
| Cheung GS, Liu CS. A retrospective study of endodontic treatment outcome between nickel-titanium rotary and stainless steel hand filing techniques. Journal of Endodontics. 2009;35(7):938-43. | Permanent teeth |
| Chien MM, Setzer S, Cleaton-Jones P. How does zinc oxide-eugenol compare to ferric sulphate as a pulpotomy material? SADJ. 2001;56(3):130-5. | No RCT |
| Childers M, Reader A, Meyers W, Nist R, Beck M. The combination IAN/PDL for mandibular first molar anesthsia [abstract]. Journal of Endodontics. 1994;20(4):191, Abstract no: RS13. | Anesthesia |
| Childers M, Reader A, Nist R, Beck M, Meyers WJ. Anesthetic efficacy of the periodontal ligament injection after an inferior alveolar nerve block. Journal of Endodontics. 1996;22(6):317-20. | Anesthesia |
| Chilton NW. Clinical evaluation of prilocaine hydrochloride 4 percent solution with and without epinephrine. Journal of the American Dental Association. 1971;83(1):149-54. | Anesthesia |
| Chohayeb AA, Adrian JC, Salamat K. PULPAL RESPONSE TO TRICALCIUM PHOSPHATE AS A CAPPING AGENT. Oral Surgery Oral Medicine Oral Pathology Oral Radiology and Endodontics. 1991;71(3):343-5. | Animal study |
| Chosack A, Sela J, CleatonJones P. A histological and quantitative histomorphometric study of apexification of nonvital permanent incisors of vervet monkeys after repeated root filling with a calcium hydroxide paste. Endodontics & Dental Traumatology. 1997;13(5):211-7. | Permanent teeth |
| Chu CH, Lo EC, Cheung GS. Outcome of root canal treatment using Thermafil and cold lateral condensation filling techniques. International Endodontic Journal. 2005;38(3):179-85. | Permanent teeth |
| Chu FCS, Tsang CSP, Chow TW, Samaranayake LP. Identification of cultivable microorganisms from primary endodontic infections with exposed and unexposed pulp space. Journal of Endodontics. 2005;31(6):424-9. | In vitro study |
| Chuansumrit A, Suwannuraks M, Sri-Udomporn N, Pongtanakul B, Worapongpaiboon S. Recombinant activated factor VII combined with local measures in preventing bleeding from invasive dental procedures in patients with Glanzmann thrombasthenia. Blood Coagulation & Fibrinolysis. 2003;14(2):187-90. | General medical problem |
| Chueh LH, Huang GT. Immature teeth with periradicular periodontitis or abscess undergoing apexogenesis: a paradigm shift. Journal of Endodontics. 2006;32(12):1205-13. | Permanent teeth |
| Ciftci A, Vardarli DA, Sonmez IS. Coronal microleakage of four endodontic temporary restorative materials: an in vitro study. Oral Surgery Oral Medicine Oral Pathology Oral Radiology & Endodontics. 2009;108(4):e67-70. | In vitro study |
| Cipollini ML, Stiles EW. FRUIT ROT, ANTIFUNGAL DEFENSE, AND PALATABILITY OF FLESHY FRUITS FOR FRUGIVOROUS BIRDS. Ecology. 1993;74(3):751-62. | Irrelevant |
| Citron CI. The clinical and histological evaluation of cresatin with calcium hydroxide on the human dental pulp. ASDC Journal of Dentistry for Children. 1977;44(4):294. | Review |
| Clarkson RM, Podlich HM, Savage NW, Moule AJ. A survey of sodium hypochlorite use by general dental practitioners and endodontists in Australia. Australian Dental Journal. 2003;48(1):20-6. | Epidemiologic study |
| Cleaton-Jones P, Duggal M, Parak M, William S, Setze S. Ferric sulphate and formocresol pulpotomies in baboon primary molars: histological responses. European journal of paediatric dentistry. 2002;3(3):121-5. | Animal study |
| Cleaton-Jones P, Duggal M, Parak R, Williams S, Setzer S. Pulpitis induction in baboon primary teeth using carious dentine or Streptococcus mutans. SADJ. 2004;59(3):119-22. | Animal study |
| Coggins R, Reader A, Nist R, Beck M, Meyers WJ. Anesthetic efficacy of the intraosseous injection in maxillary and mandibular teeth. Oral Surgery, Oral Medicine, Oral Pathology, Oral Radiology, and Endodontics. 1996;81(6):634-41. | Anesthesia |
| Cogulu D, Oncag O, Kutukculer N, Uzel A, Eronat C. The correlation between serum immunoglobulin A and immunoglobulin G levels and the presence of Treponema denticola in human periapical lesions. Journal of Endodontics. 2007;33(12):1413-6. | Immunology |
| Collaert B, Attstrˆm R, Edwardsson S, Hase J, et al. Effect of delmopinol on plaque and gingivitis [abstract]. Journal of Dental Research. 1990;69(Spec Iss):277, Abstract no: 1349. | Periodontology |
| Conner DA, Caplan DJ, Teixeira FB, Trope M. Clinical outcome of teeth treated endodontically with a nonstandardized protocol and root filled with resilon. Journal of Endodontics. 2007;33(11):1290-2. | Permanent teeth |
| Conti TR, Sakai VT, Fornetti AP, Moretti AB, Oliveira TM, Lourenco Neto N, et al. Pulpotomies with Portland cement in human primary molars. Journal of Applied Oral Science. 2009;17(1):66-9. | Case report |
| Cordaro L, Torsello F, Miuccio MT, di Torresanto VM, Eliopoulos D. Mandibular bone harvesting for alveolar reconstruction and implant placement: subjective and objective cross-sectional evaluation of donor and recipient site up to 4 years. Clinical Oral Implants Research. 2011;22(11):1320-6. | Implantology |
| Cornelini R, Cangini F, Covani U, Wilson TG. Immediate restoration of implants placed into fresh extraction sockets for single-tooth replacement: A prospective clinical study. International Journal of Periodontics & Restorative Dentistry. 2005;25(5):439-47. | Implantology |
| Cortinas-Saenz M, Martinez-Gomez L, Roncero-Goig M, Saez-Cuesta U, Ibarra-Martin M. Results of a major ambulatory oral surgery program using general inhalational anesthesia on disabled patients. Medicina Oral Patologia Oral Y Cirugia Bucal. 2009;14(11):E605-E11. | Anesthesia |
| Couteau D, Mathaly P. Purification of ferulic acid by adsorption after enzymic release from a sugar-beet pulp extract. Industrial Crops and Products. 1997;6(3-4):237-52. | In vitro study |
| Cowan A. Treatment of exposed vital pulps with a corticosteroid antibiotic agent. British Dental Journal. 1966;120(11):521-32. | Biomaterials not compared |
| Cozzi G, Gottardo F, Mattiello S, Canali E, Scanziani E, Verga M, et al. The provision of solid feeds to veal calves: I. Growth performance, forestomach development, and carcass and meat quality. Journal of Animal Science. 2002;80(2):357-66. | Irrelevant |
| Crespo S, Cortes O, Garcia C, Perez L. Comparison between rotary and manual instrumentation in primary teeth. Journal of Clinical Pediatric Dentistry. 2008;32(4):295-8. | Biomaterials not compared |
| Crooks WG, Anderson RW, Powell BJ, Kimbrough WF. Longitudinal evaluation of the seal of IRM root end fillings. Journal of Endodontics. 1994;20(5):250-2. | Permanent teeth |
| Cruz EV, Shigetani Y, Ishikawa K, Kota K, Iwaku M, Goodis HE. A laboratory study of coronal microleakage using four temporary restorative materials. International Endodontic Journal. 2002;35(4):315-20. | In vitro study |
| Cuisia ZE, Musselman R, Schneider P, Dummett C. A study of mineral trioxide aggregate pulpotomies in primary molars [abstract]. Pediatric Dentistry. 2001;23(2):168. | Abstract only |
| Cunha RF, Pugliesi DMC, Percinoto C. Treatment of traumatized primary teeth: A conservative approach. Dental Traumatology. 2007;23(6):360-3. | Traumatology |
| Cvek M. Treatment of non-vital permanent incisors with calcium hydroxide. IV. Periodontal healing and closure of the root canal in the coronal fragment of teeth with intra-alveolar fracture and vital apical fragment. A follow-up. Odontologisk Revy. 1974;25(3):239-46. | Permanent teeth |
| Cvek M, Hollender L, Nord CE. Treatment of non-vital permanent incisors with calcium hydroxide. VI. A clinical, microbiological and radiological evaluation of treatment in one sitting of teeth with mature or immature root. Odontologisk Revy. 1976;27(2):93-108. | Permanent teeth |
| Cvek M, Mejare I, Andreasen JO. Conservative endodontic treatment of teeth fractured in the middle or apical part of the root. Dental Traumatology. 2004;20(5):261-9. | Permanent teeth |
| Cvetkovic T. [Results of pulp disease therapy with dentinogenic paste "Antipulpit I"]. [Croatian]. Stomatoloski Vjesnik - Stomatological Review. 1968;2(1-6):189-203. | Permanent teeth |
| Czerninsky R, Benoliel R, Sharav Y. Odontalgia in vascular orofacial pain. Journal of Orofacial Pain. 1999;13(3):196-200. | General medical problem |
| d'Aquino R, De Rosa A, Laino G, Caruso F, Guida L, Rullo R, et al. Human Dental Pulp Stem Cells: From Biology to Clinical Applications. Journal of Experimental Zoology Part B-Molecular and Developmental Evolution. 2009;312B(5):408-15. | Biological study |
| d'Aquino R, Papaccio G, Laino G, Graziano A. Dental pulp stem cells: A promising tool for bone regeneration. Stem Cell Reviews. 2008;4(1):21-6. | Biological study |
| D'Cruz L. An investigation of the relative efficacy of Buckley's Formocresol and calcium hydroxide in primary molar vital pulp therapy [letter]. British Dental Journal. 2000;188(8):417. | Letter |
| Da Costa CC, Oshima HMS, Costa Filho LC. Evaluation of shear bond strength and interfacial micromorphology of direct restorations in primary and permanent teeth - An in vitro study. General Dentistry. 2008;56(1):85-93. | Restorative dentistry |
| da Silva GN, Braz MG, de Camargo EA, Salvadori DMF, Ribeiro DA. Genotoxicity in primary human peripheral lymphocytes after exposure to regular and white mineral trioxide aggregate. Oral Surgery, Oral Medicine, Oral Pathology, Oral Radiology, and Endodontics. 2006;102(5):e50-e4. | In vitro study |
| Damle SG, Nadkarni U. A comparative study of two root-canal resorbable filling materials in primary molars [abstract]. International Journal of Paediatric Dentistry. 1999;9(Suppl 1):86, Abstract no: 35.13. | No RCT |
| Dammaschke T, Witt M, Ott K, Schafer E. Scanning electron microscopic investigation of incidence, location, and size of accessory foramina in primary and permanent molars. Quintessence International. 2004;35(9):699-705. | In vitro study |
| Dang J, Wilder-Smith P, Peavy GM. Clinical preconditions and treatment modality: Effects on pulp surgery outcome. Lasers in Surgery and Medicine. 1998;22(1):25-9. | Permanent teeth |
| Daou M, Tavernier B, Meyer JM. Two-Year clinical evaluation of three restorative materials in primary molars. Journal of Clinical Pediatric Dentistry. 2009;34(1):53-8. | Restorative dentistry |
| Daou MH, Attin T, Gohring TN. Clinical success of compomer and amalgam restorations in primary molars. Follow up in 36 months. Schweizer Monatsschrift fur Zahnmedizin. 2009;119(11):1082-8. | Restorative dentistry |
| Daou MH, Tavernier B, Meyer JM. Clinical evaluation of four different dental restorative materials: one-year results. Schweizer Monatsschrift fur Zahnmedizin = Revue mensuelle suisse d'odonto-stomatologie = Rivista mensile svizzera di odontologia e stomatologia / SSO. 2008;118(4):290-5. | Restorative dentistry |
| Day PF, Gregg TA, Ashley P, Welbury RR, Cole BO, High AS, et al. Periodontal healing following avulsion and replantation of teeth: a multi-centre randomized controlled trial to compare two root canal medicaments. Dental Traumatology. 2012;28(1):55-64. | Traumatology |
| De Andrade AKM, Duarte RM, Guedes Lima SJ, Passos TA, Lima KC, Montes M. Nanohybrid versus nanofill composite in class I cavities: margin analysis after 12 months. Microscopy Research and Technique. 2011;74(1):23-7. | Restorative dentistry |
| De Andrade AKM, Duarte RM, Medeiros ESF, Batista AUD, Lima KC, Pontual M, et al. 30-Month randomised clinical trial to evaluate the clinical performance of a nanofill and a nanohybrid composite. Journal of Dentistry. 2011;39(1):8-15. | Restorative dentistry |
| de Brito LC, da Rosa MA, Lopes VS, e Ferreira EF, Vieira LQ, Sobrinho AP. Brazilian HIV-infected population: assessment of the needs of endodontic treatment in the post-highly active antiretroviral therapy era. Journal of Endodontics. 2009;35(9):1178-81. | General medical problem |
| De Carvalho Cardoso L, Poi WR, Panzarini SR, Sonoda CK, Da Silveira Rodrigues T, Manfrin TM. Knowledge of firefighters with special paramedic training of the emergency management of avulsed teeth. Dental Traumatology. 2009;25(1):58-63. | Surgery |
| de Chevigny C, Dao TT, Basrani BR, Marquis V, Farzaneh M, Abitbol S, et al. Treatment outcome in endodontics: the Toronto study--phases 3 and 4: orthograde retreatment. Journal of Endodontics. 2008;34(2):131-7. | Permanent teeth |
| de Sousa DL, de Sousa R, Pinto DN, Neto J, de Carvalho CBM, de Almeida PC. Antibacterial Effects of Chemomechanical Instrumentation and Calcium Hydroxide in Primary Teeth With Pulp Necrosis. Pediatric Dentistry. 2011;33(4):307-11. | Incisors |
| De-Deus G, Canabarro A, Alves G, Linhares A, Senne MI, Granjeiro JM. Optimal cytocompatibility of a bioceramic nanoparticulate cement in primary human mesenchymal cells. Journal of Endodontics. 2009;35(10):1387-90. | In vitro study |
| Dechaume M, Peri G, Nacht M. [Clinical studies on the effect of niflumic acid in stomatology]. [French]. Revue de Stomatologie et de Chirurgie Maxillo-Faciale. 1968;69(4):315-7. | Stomatology |
| Deliperi S, Bardwell DN. Two-year clinical evaluation of nonvital tooth whitening and resin composite restorations. Journal of Esthetic & Restorative Dentistry: Official Publication of the American Academy of Esthetic Dentistry. 2005;17(6):369-78; discussion 79. | Restorative dentistry |
| Demarchi MG, Sato EF. Leakage of interim post and cores used during laboratory fabrication of custom posts. Journal of Endodontics. 2002;28(4):328-9. | In vitro study |
| Deveaux E, Hildelbert P, Neut C, Romond C. Bacterial microleakage of Cavit, IRM, TERM, and Fermit: A 21-day in vitro study. Journal of Endodontics. 1999;25(10):653-9. | In vitro study |
| DiAngelis AJ, Andreasen JO, Ebeleseder KA, Kenny DJ, Trope M, Sigurdsson A, et al. International Association of Dental Traumatology guidelines for the management of traumatic dental injuries: 1. Fractures and luxations of permanent teeth. Dental Traumatology. 2012;28(1):2-12. | Traumatology |
| Dietschi D, Ardu S, Rossier-Gerber A, Krejci I. Adaptation of adhesive post and cores to dentin after in vitro occlusal loading: Evaluation of post material influence. Journal of Adhesive Dentistry. 2006;8(6):409-19. | In vitro study |
| Dodson TB. Reconstruction of alveolar bone defects after extraction of mandibular third molars: a pilot study. Oral Surgery, Oral Medicine, Oral Pathology, Oral Radiology, and Endodontics. 1996;82(3):241-7. | Surgery |
| Domingues-Falqueiro LM, Ferreira J, Lopes FM, Tymoszczenko A, Gioso MA. The effect of timing temporary cements to treat induced pulp necrosis in the teeth of dogs. Pesquisa Veterinaria Brasileira. 2007;27(2):85-8. | Animal study |
| Domingues-Falqueiro LM, Gioso MA. Comparison of the application of temporary endodontic dressing in teeth of dogs: histopathological and microbiological aspects. Pesquisa Veterinaria Brasileira. 2007;27(5):191-3. | Animal study |
| dos Santos CLV, Sonoda CK, Poi WR, Panzarini SR, Sundefeld M, Negri MR. Delayed replantation of rat teeth after use of reconstituted powdered milk as a storage medium. Dental Traumatology. 2009;25(1):51-7. | Animal study |
| Dourado AT, Caldas Junior A, Alves DF, Falc„o CA. Bacteriemeia during endodontic treatment in relation to the technique of biomechanical preparation: randomized clinical trial. Journal of Applied Oral Science. 2005;13(4):334-9. | Permanent teeth |
| Droter JA. Pulp therapy in primary teeth. Journal of Dentistry for Children. 1967;34(6):507-10. | Review |
| Drummond BK, Davidson LE, Williams SM, Moffat SM, Ayers KM. Outcomes two, three and four years after comprehensive care under general anaesthesia. New Zealand Dental Journal. 2004;100(2):32-7. | Anesthesia |
| Duggal M. Summary of: Pulpotomy of human primary molars with MTA and Portland cement: a randomised controlled trial COMMENT. British Dental Journal. 2009;207(3):129-. | Comment |
| Duggal MS, Toumba KJ, Sharma NK. Clinical performance of a compomer and amalgam for the interproximal restoration of primary molars: a 24-month evaluation. British dental journal. 2002;193(6):339-42. | Restorative dentistry |
| Duque C, Negrini Tde C, Sacono NT, Spolidorio DM, de Souza Costa CA, Hebling J. Clinical and microbiological performance of resin-modified glass-ionomer liners after incomplete dentine caries removal. Clinical Oral Investigations. 2009;13(4):465-71. | Restorative dentistry |
| Dutta BN, Gauba K, Tewari A, Chawla HS. Silver amalgam versus resin modified GIC class-II restorations in primary molars: twelve month clinical evaluation. Journal of the Indian Society of Pedodontics and Preventive Dentistry. 2001;19(3):118-22. | Restorative dentistry |
| Ehrenreich DW. A comparison of the effects of zinc-oxide eugenol and calcium hydroxide on carious dentin in human primary molars. ASDC J Dent Child. 1968;35(6):451-6. | Restorative dentistry |
| Eifinger FF. [Contribution to the glucocorticoid therapy of pulpitis]. [German]. Deutsche Zahnarztliche Zeitschrift. 1965;20(11):1206-19. | Permanent teeth |
| Einwag J. [Endodontics in primary dentition]. [German]. Zahnarztliche Mitteilungen. 1991;81(9):878-84. | No RCT |
| Eklund SA, Pittman JL, Smith RC. Trends in dental care among insured Americans: 1980 to 1995. Journal of the American Dental Association. 1997;128(2):171-8. | Epidemiologic study |
| Ekstrand KR, Bakhshandeh A, Martignon S. Treatment of proximal superficial caries lesions on primary molar teeth with resin infiltration and fluoride varnish versus fluoride varnish only: efficacy after 1 year. Caries Research. 2010;44(1):41-6. | Restorative dentistry |
| El Kalla IH, Garcia-Godoy F. Clinical evaluation of adhesively restored pulpotomized primary molars [abstract]. Journal of Dental Research. 2003;82(Spec Iss B):B-125, Abstract no: 0903. | Restorative dentistry |
| El-Housseiny AA, Farsi N. Sealing ability of a single bond adhesive in primary teeth. An in vivo study. International journal of paediatric dentistry / the British Paedodontic Society [and] the International Association of Dentistry for Children. 2002;12(4):265-70. | Restorative dentistry |
| el-Kalla IH, Garcia-Godoy F. Fracture strength of adhesively restored pulpotomized primary molars. Journal of Dentistry for Children. 1999;66(4):238-42. | Restorative dentistry |
| El-Meligy OA, Avery DR. Comparison of mineral trioxide aggregate and calcium hydroxide as pulpotomy agents in young permanent teeth (apexogenesis). Pediatric Dentistry. 2006;28(5):399-404. | Permanent teeth |
| El-Meligy OA, Avery DR. Comparison of apexification with mineral trioxide aggregate and calcium hydroxide. Pediatric Dentistry. 2006;28(3):248-53. | Permanent teeth |
| Eldeniz AU, Mustafa K, Orstavik D, Dahl JE. Cytotoxicity of new resin-, calcium hydroxide- and silicone-based root canal sealers on fibroblasts derived from human gingiva and L929 cell lines. International Endodontic Journal. 2007;40(5):329-37. | In vitro study |
| Elliott RD, Burkes EJ, Phillips CL, Roberts MW. CO2 laser and formoeresol pulpotomy effects on human primary pulp [abstract]. Journal of Dental Research. 1999;78(Spec Iss):386, Abstract no: 2242. | Duplicate |
| Elliott RD, Roberts MW, Burkes J, Phillips C. Evaluation of the carbon dioxide laser on vital human primary pulp tissue. Pediatric Dentistry. 1999;21(6):327-31. | No tooth decay |
| Elomaa M, Rantanen L, Nystrom M. Use of chemotherapeutic-corticoid combination in vital pulpotomy. Proceedings of the Finnish Dental Society. 1974;70(1):1-6. | No RCT |
| Elsharrawy EA, Elbaghdady YM. A double-blind comparison of a supplemental interligamentary injection of fentanyl and mepivacaine with 1:200,000 epinephrine for irreversible pulpitis. Journal of Pain & Symptom Management. 2007;33(2):203-7. | Anesthesia |
| Ercan E, Ozekinci T, Atakul F, Gul K. Antibacterial activity of 2% chlorhexidine gluconate and 5.25% sodium hypochlorite in infected root canal: in vivo study. Journal of Endodontics. 2004;30(2):84-7. | In vitro study |
| Erdemir A, Eldeniz AU, Ari H, Belli S, Esener T. The influence of irrigating solutions on the accuracy of the electronic apex locator facility in the Tri Auto ZX handpiece. International Endodontic Journal. 2007;40(5):391-7. | Apex locator study |
| Erdemir A, Eldeniz AU, Belli S. Effect of temporary filling materials on repair bond strengths of composite resins. Journal of Biomedical Materials Research Part B-Applied Biomaterials. 2008;86B(2):303-9. | Restorative dentistry |
| Eronat N, Eronat C. [Follow-up investigation of the effect of Ca(OH)2 on the teeth with necrotic pulps and immature root development and surrounding periodontal tissues]. [Turkish]. Ankara Universitesi Dis Hekimligi Fakultesi Dergisi. 1989;16(3):471-9. | Permanent teeth |
| Ersin NK, Aykut A, Candan U, Oncag O, Eronat C, Kose T. The effect of a chlorhexidine containing cavity disinfectant on the clinical performance of high-viscosity glass-ionomer cement following ART: 24-month results. American Journal of Dentistry. 2008;21(1):39-43. | Restorative dentistry |
| Espina AI, Castellanos AV, Fereira JL. Age-related changes in blood capillary endothelium of human dental pulp: an ultrastructural study. International Endodontic Journal. 2003;36(6):395-403. | Structural study |
| Esposito P, Varvara G, Caputi S, Perinetti G. Catalase activity in human healthy and inflamed dental pulps. International Endodontic Journal. 2003;36(9):599-603. | In vitro study |
| Facer SR, Walton RE. Intracanal distribution patterns of sealers after lateral condensation. Journal of Endodontics. 2003;29(12):832-4. | Permanent teeth |
| Fadavi S, Anderson AW. A comparison of the pulpal response to freeze-dried bone, calcium hydroxide, and zinc oxide-eugenol in primary teeth in two cynomolgus monkeys.[Erratum appears in Pediatr Dent. 2004 Jul-Aug;26(4):301]. Pediatric Dentistry. 1996;18(1):52-6. | Animal study |
| Fagundes TC, Barata TJ, Carvalho CA, Franco EB, van Dijken JW, Navarro MF. Clinical evaluation of two packable posterior composites: a five-year follow-up. Journal of the American Dental Association. 2009;140(4):447-54. | Restorative dentistry |
| Fagundes TC, Barata TJE, Bresciani E, Cefaly DFG, Jorge MFF, Navarro MFL. Clinical evaluation of two packable posterior composites: 2-year follow-up. Clinical Oral Investigations. 2006;10(3):197-203. | Restorative dentistry |
| Fallacara DM, Halbrook RS, French JB. TOXIC EFFECTS OF DIETARY METHYLMERCURY ON IMMUNE SYSTEM DEVELOPMENT IN NESTLING AMERICAN KESTRELS (FALCO SPARVERIUS). Environmental Toxicology and Chemistry. 2011;30(6):1328-37. | Irrelevant |
| Falster CA, Araujo FB, Nˆr JE, Straffon LH. A clinical and radiographic evaluation of indirect pulp capping in deciduous molars [abstract]. Journal of Dental Research. 2001;80(4):1075, Abstract no: 608. | Indirect pulp capping only |
| Falster CA, Araujo FB, Straffon LH, Nor JE. Indirect pulp treatment: in vivo outcomes of an adhesive resin system vs calcium hydroxide for protection of the dentin-pulp complex. Pediatric Dentistry. 2002;24(3):241-8. | Indirect pulp capping only |
| Fantoni R. [Histological study of the effect of dimethylsulfoxide (DMSO) on the human dental pulp]. [German]. SSO: Schweizerische Monatsschrift fur Zahnheilkunde. 1970;80(7):816-25. | Histological study |
| Fava LR. One-appointment root canal treatment: incidence of postoperative pain using a modified double-flared technique. International Endodontic Journal. 1991;24(5):258-62. | Permanent teeth |
| Feda M, Amoudi NA, Sharaf A, Hanno A, Farsi N, Masoud I, et al. A comparative study of children's pain reactions and perceptions to AMSA injection using CCLAD versus traditional injections. Journal of Clinical Pediatric Dentistry. 2010;34(3):217-22. | Anesthesia |
| Fehrenbacher JC, Sun XLX, Locke EE, Henry MA, Hargreaves KM. Capsaicin-evoked iCGRP release from human dental pulp: A model system for the study of peripheral neuropeptide secretion in normal healthy tissue. Pain. 2009;144(3):253-61. | In vitro study |
| Ferrari M, Vichi A, Mannocci F, Mason PN. Retrospective study of the clinical performance of fiber posts. American Journal of Dentistry. 2000;13(Spec No):9B-13B. | Permanent teeth |
| Ferreira DC, Brito DG, Cavalcanti BN. Cytokine production from human primary teeth pulp fibroblasts stimulated by different pulpotomy agents. Journal of Dentistry for Children (Chicago, Ill. 2009). 76(3):194-8. | In vitro study |
| Ferreira FBD, Rabang HRC, Pinheiro ET, Gade-Nein CR, Zaia AA, Ferraz CCR, et al. Root canal microbiota of dogs' teeth with periapical lesions induced by two different methods. Oral Surgery Oral Medicine Oral Pathology Oral Radiology and Endodontics. 2006;102(4):564-70. | Animal study |
| Ferreira MB, Myiagi S, Nogales CG, Campos MS, Lage-Marques JL. Time- and concentration-dependent cytotoxicity of antibiotics used in endodontic therapy. Journal of Applied Oral Science. 2010;18(3):259-63. | Antibiotics |
| Figdor D, Sundqvist G. A big role for the very small--understanding the endodontic microbial flora. Australian Dental Journal. 2007;52(1 Suppl):S38-51. | In vitro study |
| Filippi A, Pohl Y, von Arx T. Treatment of replacement resorption by intentional replantation, resection of the ankylosed sites, and Emdogain--results of a 6-year survey. Dental Traumatology. 2006;22(6):307-11. | Surgery |
| Fjerdingen H, Houen PJ, Tech Assoc P, Paper IND. On the effect of recycling of kraft paper on selected fines properties. 1997 Recycling Symposium. 1997:299-311. | Irrelevant |
| Fleming CH, Litaken MS, Alley LW, Eleazer PD. Comparison of Classic Endodontic Techniques versus Contemporary Techniques on Endodontic Treatment Success. Journal of Endodontics. 2010;36(3):414-8. | Permanent teeth |
| Foley J. A comparison of knowledge of local analgesia, pulp therapy and restoration of primary molar teeth amongst dental students, dentists and dental therapists within a dental hospital setting. European Archives of Paediatric Dentistry: Official Journal of the European Academy of Paediatric Dentistry. 2007;8(2):113-7. | Epidemiologic study |
| Folwaczny M, Hickel R. [Aspects of dental care of immunosuppressed patients. I]. [Review] [148 refs] [French, German]. Schweizer Monatsschrift fur Zahnmedizin. 2001;111(10):1201-24. | General medical problem |
| Folwaczny M, Loher C, Mehl A, Kunzelmann KH, Hickel R. Class V lesions restored with four different tooth-colored materials--3-year results. Clinical oral investigations. 2001;5(1):31-9. | Restorative dentistry |
| Folwaczny M, Loher C, Mehl A, Kunzelmann KH, Hinkel R. Tooth-colored filling materials for the restoration of cervical lesions: a 24-month follow-up study. Operative dentistry. 2000;25(4):251-8. | Restorative dentistry |
| Forte SG, Hauser MJ, Hahn C, Hartwell GR. Microleakage of super-EBA with and without finishing as determined by the fluid filtration method. Journal of Endodontics. 1998;24(12):799-801. | In vitro study |
| Fox K, Gutteridge DL. An in vitro study of coronal microleakage in root-canal-treated teeth restored by the post and core technique. International Endodontic Journal. 1997;30(6):361-8. | In vitro study |
| Frankl SN. Pulp therapy in pedodontics. [Review] [131 refs]. Oral Surgery, Oral Medicine, Oral Pathology. 1972;34(2):293-309. | Review |
| Fransson H, Petersson K, Davies JR. Dentine sialoprotein and collagen I expression after experimental pulp capping in humans using emdogain gel. International Endodontic Journal. 2011;44(3):259-67. | In vitro study |
| Franzon R, Casagrande L, Pinto AS, Garcia-Godoy F, Maltz M, de Araujo FB. Clinical and radiographic evaluation of indirect pulp treatment in primary molars: 36 months follow-up. American Journal of Dentistry. 2007;20(3):189-92. | Indirect pulp capping only |
| Franzon R, Gomes M, Pitoni CM, Bergmann CP, Araujo FB. Dentin rehardening after indirect pulp treatment in primary teeth. Journal of Dentistry for Children (Chicago, Ill. 2009). 76(3):223-8. | Indirect pulp capping only |
| Fratkin RD, Kenny DJ, Johnston DH. Evaluation of a laser Doppler flowmeter to assess blood flow in human primary incisor teeth. Pediatric Dentistry. 1999;21(1):53-6. | Incisor |
| Freed JR, Bernstein AS. Utilization of emergency dental services by rural, nonreservation Indians. Journal of Public Health Dentistry. 1975;35(03):165-9. | Epidemiologic study |
| Fridstrom M, Schollin J, Crossner CG. Evaluating Emdogain and healing of replanted teeth using an intra-individual experimental-control study design. Dental Traumatology. 2008;24(3):299-304. | Surgery |
| Friedman S, Lost C, Zarrabian M, Trope M. Evaluation of success and failure after endodontic therapy using a glass ionomer cement sealer. Journal of Endodontics. 1995;21(7):384-90. | Restorative dentistry |
| Frujeri MDV, Costa ED. Effect of a single dental health education on the management of permanent avulsed teeth by different groups of professionals. Dental Traumatology. 2009;25(3):262-71. | Permanent teeth |
| Fuks A, Papagiannoulis L, Geki S, Koulatzidou M, Po-Lychronopoulou A. One year comparative stuidy in pulpotomized primary teeth using ferric sulphate and diluted formocresol [abstract]. European Journal of Paediatric Dentistry. 2000;1(3):128. | Abstract only |
| Fuks AB. Vital pulp therapy with new materials for primary teeth: new directions and Treatment perspectives. Pediatric dentistry. 2008;30(3):211-9. | Review |
| Fuks AB, Eidelman E, CleatonJones P, Michaeli Y. Pulp response to ferric sulfate, diluted formocresol and IRM in pulpotomized primary baboon teeth. Journal of Dentistry for Children. 1997;64(4):254-&. | Animal study |
| Fuks AB, Holan G, Davis J, Eidelman E. Ferric sulfate vs. diluted formocresol in pulpotomized primary molars: clinical and radiographic report [abstract]. Journal of Dental Research. 1996;75(5 [Divisional Abstracts]):1253, Abstract no: 55. | Duplicate |
| Full CA. Pulpotomy treatment of fistulated primary molars. Quintessence International. 1979;10(10):73-8. | Biomaterials not compared |
| Gagliani MM, Gorni FGM, Strohmenger L. Periapical resurgery versus periapical surgery: a 5-year longitudinal comparison. International Endodontic Journal. 2005;38(5):320-7. | Surgery |
| Gama TG, de Oliveira JC, Abad EC, Rocas IN, Siqueira JF, Jr. Postoperative pain following the use of two different intracanal medications. Clinical Oral Investigations. 2008;12(4):325-30. | Permanent teeth |
| Gani O, Visvisian C. Apical canal diameter in the first upper molar at various ages. Journal of Endodontics. 1999;25(10):689-91. | Anatomic study |
| Gehlhoff WS, Tappi. The benefits of using enzymes to improve flotation deinking of office paper furnishes. 1998 Tappi Recycling Symposium. 1998. | Irrelevant |
| Gesi A, Hakeberg M, Warfvinge J, Bergenholtz G. Incidence of periapical lesions and clinical symptoms after pulpectomy--a clinical and radiographic evaluation of 1- versus 2-session treatment. Oral Surgery Oral Medicine Oral Pathology Oral Radiology & Endodontics. 2006;101(3):379-88. | Permanent teeth |
| Ghoddusi J, Javidi M, Zarrabi MH, Bagheri H. Flare-ups incidence and severity after using calcium hydroxide as intracanal dressing. New York State Dental Journal. 2006;72(4):24-8. | Permanent teeth |
| Gibson R, Howlett P, Cole BO. Efficacy of spirally filled versus injected non-setting calcium hydroxide dressings. Dental Traumatology. 2008;24(3):356-9. | Permanent teeth |
| Gilthorpe MS, Maddick IH, Petrie A. Introduction to Bayesian modelling in dental research. Community Dental Health. 2000;17(4):218-21. | Statistical study |
| Gimbel M, Correa A, Lin LM. Calcium hydroxide as a temporary filling of the post space in root-filled teeth. Oral Surgery Oral Medicine Oral Pathology Oral Radiology & Endodontics. 2002;94(1):98-102. | Permanent teeth |
| Gjorgievska E, Nicholson JW, Iljovska S, Slipper IJ. Marginal adaptation and performance of bioactive dental restorative materials in deciduous and young permanent teeth. Journal of applied oral science : revista FOB. 2008;16(1):1-6. | Restorative dentistry |
| Gjorup H, Haubek D, Hintze H, Haukali G, Lovschall H, Hertz JM, et al. Hypocalcified type of amelogenesis imperfecta in a large family: clinical, radiographic, and histological findings, associated dento-facial anomalies, and resulting treatment load. Acta Odontologica Scandinavica. 2009;67(4):240-7. | Permanent teeth |
| Gleicher H, Fuks AB, Sela J. Adaptation of Class II Vitremer restorations with and without primer: a morphometric study. Pediatric dentistry. 1998;20(4):263-6. | Restorative dentistry |
| Glendor U, Halling A, Andersson L, Andreasen JO, Klitz I. Type of treatment and estimation of time spent on dental trauma--a longitudinal and retrospective study. Swedish Dental Journal. 1998;22(1-2):47-60. | Traumatology |
| Glendor U, Koucheki B, Halling A. Risk evaluation and type of treatment of multiple dental trauma episodes to permanent teeth. Endodontics & Dental Traumatology. 2000;16(5):205-10. | Permanent teeth |
| Goldstein BH, Sciubba JJ, Laskin DM. Actinomycosis of the maxilla: review of literature and report of case. [Review] [32 refs]. Journal of Oral Surgery. 1972;30(5):362-6. | Review |
| Gonnissen H, Politis C, Schepers S, Lambrichts I, Vrielinck L, Sun Y, et al. Long-term success and survival rates of autogenously transplanted canines. Oral Surgery, Oral Medicine, Oral Pathology, Oral Radiology and Endodontology. 2010;110(5):570-8. | Surgery |
| Gontijo IT, Navarro RS, Ciamponi AL, Zezell DM. Whitening techniques using the diode laser and halogen lamp in human devitalized primary teeth. Journal of Dentistry for Children (Chicago, Ill. 2008). 75(2):164-7. | Whitening |
| Gonzalez-Castillo S, Bailon-Sanchez ME, Gonzalez-Rodriguez MP, Poyatos-Martinez R, Ferrer-Luque CM. An in vitro evaluation of two dentine adhesive systems to seal the pulp chamber using a glucose penetration model. Medicina Oral Patologia Oral Y Cirugia Bucal. 2011;16(4):E556-E60. | In vitro study |
| Gordon TE, Jr. Calcium-tetracycline chelate as a pulp capping agent. Journal of Oral Therapeutics & Pharmacology. 1967;4(1):49-59. | Permanent teeth |
| Gorduysus MO, Gorduysus MG. Endodontic patient profile of Hacettepe University, Faculty of Dentistry in Ankara, Turkey. International Dental Journal. 2000;50(5):274-8. | Permanent teeth |
| Gotze Gda R, Cunha CB, Primo LS, Maia LC. Effect of the sodium hypochlorite and citric acid association on smear layer removal of primary molars. Pesquisa Odontologica Brasileira = Brazilian Oral Research. 2005;19(4):261-6. | Restorative dentistry |
| Goumans C, Veerkamp JS, Aartman IH. Dental anxiety and behavioural problems: what is their influence on the treatment plan? European Journal of Paediatric Dentistry. 2004;5(1):15-8. | Influence of dental anxiety |
| Gound TG, Sather JP, Kong TS, Makkawy HA, Marx DB. Graduating dental students' ability to produce quality root canal fillings using single- or multiple-cone obturation techniques. Journal of Dental Education. 2009;73(6):696-705. | Permanent teeth |
| Griffin KM, Pickering M, O'Herlihy C, O'Connell PR, Jones JFX. Sacral nerve stimulation increases activation of the primary somatosensory cortex by anal canal stimulation in an experimental model. British Journal of Surgery. 2011;98(8):1160-9. | Irrelevant |
| Griffin SO, Gooch BF, Beltran E, Sutherland JN, Barsley R. Dental services, costs, and factors associated with hospitalization for Medicaid-eligible children, Louisiana 1996-97. Journal of Public Health Dentistry. 2000;60(1):21-7. | Epidemiologic study |
| Grivu O, Mecher E, Stoica E. [Principles and present status of the therapy of pulp diseases in children]. [Romanian]. Stomatologia. 1966;13(3):233-44. | No RCT |
| Gross LC, Griffen AL, Casamassimo PS. Compomers as Class II restorations in primary molars. Pediatric dentistry. 2001;23(1):24-7. | Restorative dentistry |
| Gruythuysen RJ, Weerheijm KL, Hagen JW. Calcium hydroxide pulpotomy with light-cured cavity-sealing after two years [abstract]. International Dental Journal. 1995;45:313. | Abstract only |
| Gu Y, Lu Q, Wang P, Ni L. Root canal morphology of permanent three-rooted mandibular first molars: Part II--measurement of root canal curvatures. Journal of Endodontics. 2010;36(8):1341-6. | Permanent teeth |
| Guelmann M, Bookmyer KL, Villalta P, Garcia-Godoy F. Microleakage of restorative techniques for pulpotomized primary molars. Journal of Dentistry for Children (Chicago, Ill. 2004). 71(3):209-11. | In vitro study |
| Guglielmi CAB, Ramalho KM, Scaramucci T, da Silva S, Imparato JCP, Pinheiro SL. Evaluation of the furcation area permeability of deciduous molars treated by neodymium:yttrium-aluminum-garnet laser or adhesive. Lasers in Medical Science. 2010;25(6):873-80. | In vitro study |
| Hˆrsted-Bindslev P, Heyde-Petersen B, Simonsen P, Baelum V. Tunnel or saucer-shaped restorations: a survival analysis. Clinical Oral Investigations. 2005;9(4):233-8. | Restorative dentistry |
| Hagge MS, Wong RDM, Lindemuth JS. Retention of posts luted with phosphate monomer-based composite cement in canals obturated using a eugenol sealer. American Journal of Dentistry. 2002;15(6):378-82. | Permanent teeth |
| Hallett KB, Radford DJ, Seow WK. Oral health of children with congenital cardiac diseases: a controlled study. Pediatric Dentistry. 1992;14(4):224-30. | General medical problem |
| Hamilton JC, Dennison JB, Stoffers KW, Welch KB. A clinical evaluation of air-abrasion treatment of questionable carious lesions. A 12-month report. Journal of the American Dental Association. 2001;132(6):762-9. | Restorative dentistry |
| Hamre HJ, Mittag I, Glockmann A, Kiene H, Troger W. Pulpa dentis D30 for acute reversible pulpitis: A prospective cohort study in routine dental practice. Alternative Therapies in Health & Medicine. 2011;17(1):16-21. | No RCT |
| Hannah DR. Glutaraldehyde and calcium hydroxide. A pulp dressing material. British Dental Journal. 1972;132(6):227-31. | No RCT |
| Hannah DR. Glutaraldehyde for pulpotomy [letter]. British Dental Journal. 1972;132(12):438. | Letter |
| Hannan L, Reader A, Nist R, Beck M, Meyers WJ. The use of ultrasound for guiding needle placement for inferior alveolar nerve blocks. Oral Surgery, Oral Medicine, Oral Pathology, Oral Radiology, and Endodontics. 1999;87(6):658-65. | Anaesthesia |
| Hansen H. [Corticoids in endodontia. A clinical-histological study of 109 cases]. [Danish]. Tandlaegebladet. 1969;73(7):539-56. | Permanent teeth |
| Hansen HP, Ravn JJ, Ulrich D. Vital pulpotomy in primary molars. A clinical and histologic investigation of the effect of zinc oxide-eugenol cement and Ledermix. Scandinavian Journal of Dental Research. 1971;79(1):13-25. | Review |
| Hansen SR, Montgomery S. Effect of restoration thickness on the sealing ability of TERM. Journal of Endodontics. 1993;19(9):448-52. | Restorative dentistry |
| Harada K, Baba Y, Ohyama K, Omura K. Soft tissue profile changes of the midface in patients with cleft lip and palate following maxillary distraction osteogenesis: a preliminary study. Oral Surgery, Oral Medicine, Oral Pathology, Oral Radiology, and Endodontics. 2002;94(6):673-7. | General medical problem |
| Harada K, Baba Y, Ohyama K, Omura K. Soft tissue profile changes of the midface in patients with cleft lip and palate following maxillary distraction osteogenesis: a preliminary study. Oral Surgery, Oral Medicine, Oral Pathology, Oral Radiology, and Endodontics. 2002;94(6):673-7. | In vitro study |
| Harada K, Baba Y, Ohyama K, Omura K. Soft tissue profile changes of the midface in patients with cleft lip and palate following maxillary distraction osteogenesis: a preliminary study. Oral Surgery, Oral Medicine, Oral Pathology, Oral Radiology, and Endodontics. 2002;94(6):673-7.352. Harini Priya M, Bhat SS, Sundeep Hegde K. Comparative evaluation of bactericidal potential of four root canal filling materials against microflora of infected non-vital primary teeth. Journal of Clinical Pediatric Dentistry. 2010;35(1):23-9. | Ex vivo |
| Harris T, Crawford PJ. Case report: teeth and tonsils: the use of culture and sensitivity testing for antibiotic prescribing in dental infection. British Dental Journal. 2007;202(8):463-4. | Case report |
| Harrison MG, Roberts GJ. Comprehensive dental treatment of healthy and chronically sick children under intubation general anaesthesia during a 5-year period. British Dental Journal. 1998;184(10):503-6. | General medical problem |
| Hartmann F. Clinical application of corticoids. International Dental Journal. 1981;31(4):273-85. | Permanent teeth |
| Hartsook JT. Pulpal therapy in primaryand young permanent teeth. Dental Clinics of North America. 1966:377-89. |  |
| Hartsook JT. Pulpal therapy in primaryand young permanent teeth. Dental Clinics of North America. 1966:377-89. | In vitro study |
| Hassan KS. Autogenous bone graft combined with polylactic polyglycolic acid polymer for treatment of dehiscence around immediate dental implants. Oral Surgery, Oral Medicine, Oral Pathology, Oral Radiology, and Endodontics. 2009;108(5):e19-25. | Implant |
| Hayashi M, Fujitani M, Yamaki C, Momoi Y. Ways of enhancing pulp preservation by stepwise excavation--a systematic review. [Review]. Journal of Dentistry. 2011;39(2):95-107. | Review |
| Hebling J, Giro EM, Costa CA. Biocompatibility of an adhesive system applied to exposed human dental pulp. Journal of Endodontics. 1999;25(10):676-82. | In vitro study |
| Heilig J, Yates J, Siskin M, McKnight J, Turner J. Calcium hydroxide pulpotomy for primary teeth: a clinical study. Journal of the American Dental Association. 1984;108(5):775-8. | Biomaterials not compared |
| Heit JM, Stevens MR, Jeffords K. Comparison of ceftriaxone with penicillin for antibiotic prophylaxis for compound mandible fractures. Oral Surgery, Oral Medicine, Oral Pathology, Oral Radiology, and Endodontics. 1997;83(4):423-6. | Surgery |
| Heithersay GS. Periapical repair following conservative endodontic therapy. Australian Dental Journal. 1970;15(6):511-8. | Permanent teeth |
| Heithersay GS. Calcium hydroxide in the treatment of pulpless teeth with associated pathology. Journal of the British Endodontic Society. 1975;8(2):74-93. | No RCT |
| Herforth A, Strassburg M. [Therapy of the chronic apical periodontitis with traumatised damaged front teeth with incomplete root growth]. Translated copy available at editorial base. 1977;32:453-9. | Front teeth |
| Hietasalo P, Seppa L, Niinimaa A, Kallio J, Lahti S, Hausen H. Post-trial costs, clinical outcomes, and dental service utilization after a randomized clinical trial for caries control among Finnish adolescents. European Journal of Oral Sciences. 2010;118(3):265-9. | Permanent teeth |
| Hill MW. The survival of vital and non-vital deciduous molar teeth following pulpotomy. Australian Dental Journal. 2007;52(3):181-6. | No RCT |
| Hiura A. Neuroanatomical effects of capsaicin on the primary afferent neurons. Archives of Histology and Cytology. 2000;63(3):199-215. | Irrelevant |
| Hiura A, Ishizuka H. QUANTITATIVE ELECTRON-MICROSCOPIC ANALYSES OF PULPAL NERVE-FIBERS IN THE MOUSE LOWER INCISOR AFTER NEONATAL CAPSAICIN TREATMENT. Archives of Oral Biology. 1992;37(12):1085-90. | In vitro study |
| Hodosh M, Hodosh SH, Shklar G, Hodosh AJ. Potassium nitrate: an effective treatment for pulpitis. Oral Surgery, Oral Medicine, Oral Pathology. 1983;55(4):419-20. | Permanent teeth |
| Holan G. Development of clinical and radiographic signs associated with dark discolored primary incisors following traumatic injuries: a prospective controlled study. Dental Traumatology. 2004;20(5):276-87. | Incisors |
| Holan G. Long-term effect of different treatment modalities for traumatized primary incisors presenting dark coronal discoloration with no other signs of injury. Dental Traumatology. 2006;22(1):14-7. | Incisors |
| Holan G, Eidelman E, Fuks AB. Mineral trioxide aggregate vs. formocresol in pulpotomized primary molars: 8-36 months follow-up [abstract]. Journal of Dental Research. 2002;81(Spec Iss B [Divisional Abstracts]):B-308, Abstract no: 37. | Duplicate |
| Holan G, Fuks AB, Ketlz N. Success rate of formocresol pulpotomy in primary molars restored with stainless steel crown vs amalga. Pediatric Dentistry. 2002;24(3):212-6. | Restorative dentistry |
| Holan G, Topf J, Fuks AB. Effect of root canal infection and treatment of traumatized primary incisors on their permanent successors. Endodontics & Dental Traumatology. 1992;8(1):12-5. | Incisors |
| Holland R, Ferreira LB, de Souza V, Otoboni JA, Murata SS, Dezan E. Reaction of the lateral periodontium of dogs' teeth to contaminated and noncontaminated perforations filled with mineral trioxide aggregate. Journal of Endodontics. 2007;33(10):1192-7. | Animal study |
| Horiuchi H, Abe S, Maeda K, Watanabe M. [Anti-inflammatory, analgesic effect of lysozyme chloride following direct pulpectomy and root canal filling]. [Japanese]. Shikai Tenbo - Dental Outlook. 1981;58(5):1007-12. | Permanent teeth |
| Horsted-Bindslev P, Vilkinis V, Sidlauskas A. Direct capping of human pulps with a dentin bonding system or with calcium hydroxide cement. Oral Surgery Oral Medicine Oral Pathology Oral Radiology & Endodontics. 2003;96(5):591-600. | Premolars |
| Horwitz E, Pisanty S, Czerninski R, Helser M, Eliav E, Touitou E. A clinical evaluation of a novel liposomal carrier for acyclovir in the topical treatment of recurrent herpes labialis. Oral Surgery, Oral Medicine, Oral Pathology, Oral Radiology, and Endodontics. 1999;87(6):700-5. | General medical problem |
| Hosoya N, Cox CF, Arai T, Nakamura J. The walking bleach procedure: an in vitro study to measure microleakage of five temporary sealing agents. Journal of Endodontics. 2000;26(12):716-8. | In vitro study |
| Hovav S, Holan G, Lewinstein I, Fuks AB. Microleakage of class 2 Superbond-lined composite restorations with and without a cervical amalgam base. Operative dentistry. 1995;20(2):63-7. | In vitro study |
| Hse KM, Wei SH. Clinical evaluation of compomer in primary teeth: 1-year results. Journal of the American Dental Association (1939). 1997;128(8):1088-96. | Restorative dentistry |
| Huang CH, Tseng WY, Yao CC, Jeng JH, Young TH, Chen YJ. Glucosamine promotes osteogenic differentiation of dental pulp stem cells through modulating the level of the transforming growth factor-beta type I receptor. Journal of Cellular Physiology. 2010;225(1):140-51. | Biological study |
| Huang GTJ, Gronthos S, Shi S. Mesenchymal Stem Cells Derived from Dental Tissues vs. Those from Other Sources: Their Biology and Role in Regenerative Medicine. Journal of Dental Research. 2009;88(9):792-806. | Biological study |
| Huang TH, Hung CJ, Chen YJ, Chien HC, Kao CT. Cytologic effects of primary tooth endodontic filling materials. Journal of Dental Sciences. 2009;4(1):18-24. | In vitro study |
| Huang TH, Liao PH, Liu CH, Yen M, Kao CT. Effect of various pulpotomy dressing materials on the viability of L929 cells. Journal of Dental Sciences. 2008;3(1):22-9. | In vitro study |
| Huang TJ, Roan RT, Lin HT. [The management of sinus tracts of dental origin]. [Chinese]. Kao-Hsiung i Hsueh Ko Hsueh Tsa Chih [Kaohsiung Journal of Medical Sciences]. 1992;8(2):89-95. | Biomaterial not compared |
| Huang XW, Zhou Y, Luo H, Cheng XF. Clinical evaluation of filling materials on the proximal side of primary molars. Heilongjiang Medical Journal. 2002;26(7):487-8. | Restorative dentistry |
| Hubel S, Mejare I. Conventional versus resin-modified glass-ionomer cement for Class II restorations in primary molars. A 3-year clinical study. International journal of paediatric dentistry / the British Paedodontic Society [and] the International Association of Dentistry for Children. 2003;13(1):2-8. | Restorative dentistry |
| Huffaker SK, Safavi K, Spangberg LS, Kaufman B. Influence of a passive sonic irrigation system on the elimination of bacteria from root canal systems: a clinical study. Journal of Endodontics. 2010;36(8):1315-8. | Permanent teeth |
| Huffman BP, Mai S, Pinna L, Weller RN, Primus CM, Gutmann JL, et al. Dislocation resistance of ProRoot Endo Sealer, a calcium silicate-based root canal sealer, from radicular dentine. International Endodontic Journal. 2009;42(1):34-46. | Permanent teeth |
| Hugo Gutierrez J. [Treatment of dentin and pulp by means of disinfectants, antibiotics and corticosteroids. Bibliographic review]. [Review] [79 refs] [Spanish]. Anales Espanoles de Odontoestomatologia. 1969;28(6):470-92. | Review |
| Hugoson A, Koch G, Bergendal T, Hallonsten AL, Slotte C, Thorstensson B, et al. Oral health of individuals aged 3-80 years in Jonkoping, Sweden in 1973, 1983, and 1993. II. Review of clinical and radiographic findings. Swedish Dental Journal. 1995;19(6):243-60. | Review |
| Hugoson A, Koch G, Gothberg C, Helkimo AN, Lundin SA, Norderyd O, et al. Oral health of individuals aged 3-80 years in Jonkoping, Sweden during 30 years (1973-2003). II. Review of clinical and radiographic findings. [Review] [0 refs]. Swedish Dental Journal. 2005;29(4):139-55. | Review |
| Hupp JG, Mesaros SV, Aukhil I, Trope M. Periodontal ligament vitality and histologic healing of teeth stored for extended periods before transplantation. Endodontics & Dental Traumatology. 1998;14(2):79-83. | Surgery |
| Ibricevic H, Al-Jame G. Ferric sulfate as pulpotomy agent in primary teeth: clinical study [abstract]. Journal of Dental Research. 2001;80(4):1216, Abstract no: 123. | Abstract only |
| Ibricevic H, Al-Jame Q, Honkala S. Pediatric dental procedures under general anesthesia at the Amiri Hospital in Kuwait. Journal of Clinical Pediatric Dentistry. 2001;25(4):337-42. | Biomaterial not compared |
| Imura N, Otani SM, Campos MJ, Jardim Junior EG, Zuolo ML. Bacterial penetration through temporary restorative materials in root-canal-treated teeth in vitro. International Endodontic Journal. 1997;30(6):381-5. | In vitro study |
| Inoue M, Kitakoji H, Yano T, Ishizaki N, Itoi M, Katsumi Y. Acupuncture treatment for low back pain and lower limb symptoms - The relation between acupuncture or electroacupuncture stimulation and sciatic nerve blood flow. Evidence-Based Complementary and Alternative Medicine. 2008;5(2):133-43. | Irrelevant |
| Island G, White GE. Polyethylene ribbon fibers: a new alternative for restoring badly destroyed primary incisors. Journal of Clinical Pediatric Dentistry. 2005;29(2):151-6. | Incisors |
| Jacobson HL, Xia T, Baumgartner JC, Marshall JG, Beeler WJ. Microbial leakage evaluation of the continuous wave of condensation. Journal of Endodontics. 2002;28(4):269-71. | In vitro study |
| Jacquot BM, Panighi MM, Steinmetz P, G'Sell C. Evaluation of temporary restorations' microleakage by means of electrochemical impedance measurements. Journal of Endodontics. 1996;22(11):586-9. | In vitro study |
| Jacquot BM, Panighi MM, Steinmetz P, Gsell C. Microleakage of Cavit, CavitW, CavitG and IRM by impedance spectroscopy. International Endodontic Journal. 1996;29(4):256-61. | In vitro study |
| Jakse N, Bankaoglu V, Wimmer G, Eskici A, Pertl C. Primary wound healing after lower third molar surgery: evaluation of 2 different flap designs. Oral Surgery, Oral Medicine, Oral Pathology, Oral Radiology, and Endodontics. 2002;93(1):7-12. | Permanent teeth |
| Jarad FD, Albadri S, Gamble C, Burnside G, Fox K, Ashley JR, et al. Working length determination in general dental practice: a randomised controlled trial. British Dental Journal. 2011;211(12):595-8. | Permanent teeth |
| Jayanty S, Song J, Rubinstein NM, Chong A, Beaudry RM. Temporal relationship between ester biosynthesis and ripening events in bananas. Journal of the American Society for Horticultural Science. 2002;127(6):998-1005. | Irrelevant |
| Jensen J, Nusstein J, Drum M, Reader A, Beck M. Anesthetic efficacy of a repeated intraosseous injection following a primary intraosseous injection. Journal of Endodontics. 2008;34(2):126-30. | Anaesthesia |
| Jerrell RG, Courts FJ, Stanley HR. A comparison of two calcium hydroxide agents in direct pulp capping of primary teeth. ASDC J Dent Child. 1984;51(1):34-8. | Canines |
| Jia RZ, Zheng SG, Gao Y, Wang J. [The histological and immunohistochemical manifestation of the immature anterior teeth after pulpotomy]. [Chinese]. Chung-Hua Kou Chiang i Hsueh Tsa Chih Chinese Journal of Stomatology. 2007;42(7):412-6. | Permanent teeth |
| Jiang W, Jiang YT, Li CL, Liang JP. Investigation of Supragingival Plaque Microbiota in Different Caries Status of Chinese Preschool Children by Denaturing Gradient Gel Electrophoresis. Microbial Ecology. 2011;61(2):342-52. | Biomaterials not compared |
| Jiang Y, Sun M, Wu D. [Clinical studies on apexification with demineralized dentin matrix]. [Chinese]. Hua Xi Kou Qiang Yi Xue Za Zhi. 2003;21(6):460-2. | Permanent teeth |
| Jittapiromsak N, Sahawat D, Banlunara W, Sangvanich P, Thunyakitpisal P. Acemannan, an Extracted Product from Aloe Vera, Stimulates Dental Pulp Cell Proliferation, Differentiation, Mineralization, and Dentin Formation. Tissue Engineering Part A. 2010;16(6):1997-2006. | Biomaterials not compared |
| Johns BA, Brown LJ, Nash KD, Warren M. The endodontic workforce. Journal of Endodontics. 2006;32(9):838-46. | Permanent teeth |
| Johnson MS, Britto LR, Guelmann M. Impact of a biological barrier in pulpectomies of primary molars. Pediatric Dentistry. 2006;28(6):506-10. | In vitro study |
| Jung IY, Lee SJ, Hargreaves KM. Biologically based treatment of immature permanent teeth with pulpal necrosis: a case series. Journal of Endodontics. 2008;34(7):876-87. | Permanent teeth |
| Kalaskar R, Damle SG, Tiku A. Nonsurgical treatment of periapical lesions using intracanal calcium hydroxide medicament--a report of 2 cases. Quintessence International. 2007;38(5):e279-84. | Case report |
| Kalaskar RR, Damle SG. Comparative evaluation of lyophilized freeze dried platelet derived preparation with calcium hydroxide as pulpotomy agents in primary molars. Journal of the Indian Society of Pedodontics & Preventive Dentistry. 2004;22(1):24-9. | No RCT |
| Kalnins V. Healing of pulps under pressure dressing in permanent teeth. Oral Surgery, Oral Medicine, Oral Pathology. 1966;22(1):100-13. | Permanent teeth |
| Kamal AMM, Okiji T, Suda H. Response of class II molecule-expressing cells and macrophages to cavity preparation and restoration with 4-META /MMA-TBB resin. International Endodontic Journal. 2000;33(4):367-73. | Biological study |
| Kampfer J, Gohring TN, Attin T, Zehnder M. Leakage of food-borne Enterococcus faecalis through temporary fillings in a simulated oral environment. International Endodontic Journal. 2007;40(6):471-7. | In vitro study |
| Kawamura M, Sasaki T, Imai-Tanaka T, Yamasaki Y, Iwamoto Y. Service-mix in general dental practice in Japan: a survey in a suburban area. Australian Dental Journal. 1998;43(6):410-6. | Survey |
| Kemppainen P, Waltimo A, Waltimo T, Kononen M, Pertovaara A. Differential effects of noxious conditioning stimulation of the cheek by capsaicin on human sensory and inhibitory masseter reflex responses evoked by tooth pulp stimulation. J-Dent-Res. 1997;76(9):1561-8. | Biomaterials not compared |
| Kerkhove BC, Jr., Herman SC, Klein AI, McDonald RE. A clinical and television densitometric evaluation of the indirect pulp capping technique. Journal of Dentistry for Children. 1967;34(3):192-201. | Indirect pulp capping only |
| Kerkis I, Ambrosio CE, Kerkis A, Martins DS, Zucconi E, Fonseca SAS, et al. Early transplantation of human immature dental pulp stem cells from baby teeth to golden retriever muscular dystrophy (GRMD) dogs: Local or systemic? Journal of Translational Medicine. 2008;6. | Surgery |
| Kerr AR, Drexel CA, Spielman AI. The efficacy and safety of 50 mg penicillin G potassium troches for recurrent aphthous ulcers. Oral Surgery, Oral Medicine, Oral Pathology, Oral Radiology, and Endodontics. 2003;96(6):685-94. | General medical problem |
| Kervin D, Coffey KA, Tech Assoc P, Paper IND. Contaminant control on a high speed paper machine. Engineering & Papermakers: Forming Bonds for Better Papermaking, Books 1-3. 1997:661-8. | Irrelevant |
| Kesler G, Koren R, Gal R. Histological changes induced by 15 F CO2 laser microprobe especially designed for root canal sterilization; in vivo study. Lasers in Dentistry Iv, Proceedings Of. 1998:182-90. | Permanent teeth |
| Kesler G, Koren R, Kesler A, Hay N, Gal R. Histological changes induced by CO(2) laser microprobe specially designed for root canal sterilization: In vivo study. Journal of Clinical Laser Medicine & Surgery. 1998;16(5):263-7. | Permanent teeth |
| Keszler A, Dominguez FV. [Current status of pulp treatment with formocresol]. [Spanish]. Revista Espanola de Endodoncia. 1987;5(2):63-70. | No RCT |
| Keszler A, Dominguez FV. [Histomorphometry of dentigerous cysts in children, associated or not with formocresol]. [Spanish]. Revista de la Asociacion Odontologica Argentina. 1990;78(4):214-7. | Cysts |
| Khademi AA, Atbaee A, Razavi SM, Shabanian M. Periodontal healing of replanted dog teeth stored in milk and egg albumen. Dental Traumatology. 2008;24(5):510-4. | Surgery |
| Kharkar VR, Kotrashetti SM. Transport dentoalveolar distraction osteogenesis-assisted rapid orthodontic canine retraction. Oral Surgery, Oral Medicine, Oral Pathology, Oral Radiology, and Endodontics. 2010;109(5):687-93. | Orthodontics |
| Kharkar VR, Rudagi BM, Halli R, Kini Y. Comparison of the modified lateral orbitotomy approach and modified hemicoronal approach in the treatment of unstable malunions of zygomatic complex fractures. Oral Surgery, Oral Medicine, Oral Pathology, Oral Radiology, and Endodontics. 2010;109(4):504-9. | Irrelevant |
| Khurshudian AV. A pilot study to test the efficacy of oral administration of interferon-alpha lozenges to patients with Sjogren's syndrome. Oral Surgery, Oral Medicine, Oral Pathology, Oral Radiology, and Endodontics. 2003;95(1):38-44. | Irrelevant |
| Kielbassa AM, Uchtmann H, Wrbas KT, Bitter K. In vitro study assessing apical leakage of sealer-only backfills in root canals of primary teeth. Journal of Dentistry. 2007;35(7):607-13. | In vitro study |
| Kierat A, Laszczynska M, Kowalska E, Weyna E. [Comparison of the influence of mineral trioxide aggregate and calcium hydroxide on dental pulp of permanent teeth in biological treatment and cell cultures]. [Polish]. Annales Academiae Medicae Stetinensis. 2010;56(2):89-96. | Permanent teeth |
| Kim JH, Kim Y, Shin SJ, Park JW, Jung IY. Tooth discoloration of immature permanent incisor associated with triple antibiotic therapy: a case report. Journal of Endodontics. 2010;36(6):1086-91. | Permanent teeth |
| Kim YK, Kim SG, Park JY, Yi YJ, Bae JH. Comparison of clinical outcomes of sinus bone graft with simultaneous implant placement: 4-month and 6-month final prosthetic loading. Oral Surgery, Oral Medicine, Oral Pathology, Oral Radiology, and Endodontics. 2011;111(2):164-9. | Implant |
| King SR, McWhorter AG, Seale NS. Concentration of formocresol used by pediatric dentists in primary tooth pulpotomy. Pediatric Dentistry. 2002;24(2):157-9. | Survey |
| Kirkevang LL, Vaeth M, Horsted-Bindslev P, Bahrami G, Wenzel A. Risk factors for developing apical periodontitis in a general population. International Endodontic Journal. 2007;40(4):290-9. | Risk factors study |
| Kirzioglu Z, Gurbuz T, Yilmaz Y. Clinical evaluation of chemomechanical and mechanical caries removal: status of the restorations at 3, 6, 9 and 12 months. Clinical oral investigations. 2007;11(1):69-76. | Restorative dentistry |
| Kitamura C, Ogawa Y, Morotomi T, Terashita M. Differential effects of capping agents on apoptosis during pulp wound healing. Dentin/Pulp Complex. 2002:135-6. | In vitro study |
| Klein U, Hunzeker C, Hutfless S, Galloway A. Quality of anesthesia for the maxillary primary anterior segment in pediatric patients: comparison of the P-ASA nerve block using CompuMed delivery system vs traditional supraperiosteal injections. Journal of Dentistry for Children (Chicago, Ill. 2005). 72(3):119-25. | Anesthesia |
| Koagel SO, Mines P, Apicella M, Sweet M. In vitro study to compare the coronal microleakage of Tempit UltraF, Tempit, IRM, and Cavit by using the fluid transport model. Journal of Endodontics. 2008;34(4):442-4. | In vitro study |
| Koba K, Kimura Y, Matsumoto K, Gomyoh H, Komi S, Harada S, et al. A clinical study on the effects of pulsed Nd:YAG laser irradiation at root canals immediately after pulpectomy and shaping. Journal of Clinical Laser Medicine & Surgery. 1999;17(2):53-6. | Permanent teeth |
| Kobayashi S, Baba H, Takeno K, Shimada S, Kubota M, Yayama T, et al. Blood Flow Analysis of Compressed Nerve Root after Intravenous Injection of Lipo-Prostaglandin E(1). Journal of Orthopaedic Research. 2009;27(9):1252-7. | Irrelevant |
| Kohut B, Mankodi S, Yost K. Chemical vs mechanical oral hygiene in treating interdental gingivitis [abstract]. Journal of Dental Research. 1988;67(Spec Iss):246, Abstract no: 1069. | Periodontology |
| Kortmann RD, Timmermann B, Kuhl J, Willich N, Flentje P, Meisner C, et al. HIT '91 (Prospective, co-operative study for the treatment of malignant brain tumors in childhood): Accuracy and acute toxicity of the irradiation of the craniospinal axis - Results of the quality assurance program. Strahlentherapie Und Onkologie. 1999;175(4):162-9. | Irrelevant |
| Kouri EM, Matthews JL, Taylor PP. Epinephrine in pulpotomy. ASDC Journal of Dentistry for Children. 1969;36(2):123-8. | No RCT |
| Koyam N, Okubo Y, Nakao K, Bessho K. Evaluation of Pluripotency in Human Dental Pulp Cells. Journal of Oral and Maxillofacial Surgery. 2009;67(3):501-6. | Biological study |
| Krage TL, Stiefel A, Stephan BM, Zimmer S, Lambrichts I, Raab WHM. Microhardness changes in dentine after neonatal capsaicin application. International Endodontic Journal. 2005;38(8):570-4. | In vitro study |
| Kramer N, Frankenberger R. Clinical performance of a condensable metal-reinforced glass ionomer cement in primary molars. British Dental Journal. 2001;190(6):317-21. | Restorative dentistry |
| Krupinska A, Krupinski J. [Late results of treatment of teeth with periapical changes by one-time method using Rezoform paste for filling root canals]. [Polish]. Czasopismo Stomatologiczne. 1971;24(8):877-85. | Permanent teeth |
| Kulkarni VV, Damle SG. Endodontic treatment of primary teeth using combination of antibacterial drugs: an in vivo study. Journal of Indian Society of Pedodontics and Preventive Dentistry. 2003;21(3):98-104. | Title only |
| Kummer TR, Calvo MC, Cordeiro MM, de Sousa Vieira R, de Carvalho Rocha MJ. Ex vivo study of manual and rotary instrumentation techniques in human primary teeth. Oral Surgery Oral Medicine Oral Pathology Oral Radiology & Endodontics. 2008;105(4):e84-92. | Ex vivo study |
| Kuvvetli SS, Sandalli N, Topcuoglu N, Kulekci G. Antibacterial Efficacy of Diode and Er:YAG Laser Irradiation in Experimentally Contaminated Primary Molar Root Canals. Journal of Clinical Pediatric Dentistry. 2009;34(1):43-8. | Experimentally contamination |
| Kwok-Tung L, King NM. Retrospective audit of caries management techniques for children under general anesthesia over an 18-year period. Journal of Clinical Pediatric Dentistry. 2006;31(1):58-62. | Retrospective audit |
| Kwon TY, Fujishima T, Imai Y. FT-Raman spectroscopy of calcium hydroxide medicament in root canals. International Endodontic Journal. 2004;37(7):489-93. | In vitro study |
| LaFond JF, Krygsveld D, Heimrich J, Hayes A, Cameron A, Tech Assoc P, et al. Irving pulp and paper recovery boiler upgrade: Advanced design techniques to increase capacity and improve cleanability. Engineering & Papermakers: Forming Bonds for Better Papermaking, Books 1-3. 1997:1365-93. | Irrelevant |
| Lai TN, Lin CP, Kok SH, Yang PJ, Kuo YS, Lan WH, et al. Evaluation of mandibular block using a standardized method. Oral Surgery, Oral Medicine, Oral Pathology, Oral Radiology, and Endodontics. 2006;102(4):462-8. | Anaesthesia |
| Lai YY, Pai L, Chen CP. Marginal leakage of different temporary restorations in standardized complex endodontic access preparations. Journal of Endodontics. 2007;33(7):875-8. | In vitro study |
| Langer M, Ulmansky M, Sela J. Behaviour of human dental pulp to Calxyl with or without zinc oxide eugenol. Archives of Oral Biology. 1970;15(3):189-94. | Permanent premolars |
| Lapa R, Watanabe I. Synaptic contacts established by inferior alveolar nerve fibres in the paratrigeminal nucleus: an electron microscopic study in the rat. Archives of Oral Biology. 2005;50(1):73-9. | In vitro study |
| Laudahn R, Kohlhoff H, Bromm B. MAGNETOENCEPHALOGRAPHY IN THE INVESTIGATION OF CORTICAL PAIN PROCESSING. Pain and the Brain: From Nociception to Cognition. 1995:267-82. | Irrelevant |
| Law K, Daneault C, Guimond R. Enhancement of TMP long fibres. Journal of Pulp and Paper Science. 2007;33(3):138-42. | Irrelevant |
| Le T, Nassery K, Kahler B, Heithersay AOG. A comparative diagnostic assessment of anterior tooth and bone status using panoramic and periapical radiography. Australian Orthodontic Journal. 2011;27(2):162-8. | Anterior tooth |
| Lee LW, Hsiao SH, Chang CC, Chen LK. Duration for apical barrier formation in necrotic immature permanent incisors treated with calcium hydroxide apexification using ultrasonic or hand filing. Journal of the Formosan Medical Association. 2010;109(8):596-602. | Permanent incisors |
| Lee SH, Kim KK, Choi BK. Upregulation of intercellular adhesion molecule 1 and proinflammatory cytokines by the major surface proteins of Treponema maltophilum and Treponema lecithinolyticum, the phylogenetic group IV oral spirochetes associated with periodontitis and endodontic infections. Infection and Immunity. 2005;73(1):268-76. | Immunology |
| Lee SH, Sohn YS, Choi YW, Park NH, Yoon HJ. Culture of Mesenchymal Stromal Cells from Dental Pulp: Culture Medium Study for Effective Expansion and Characterization. Tissue Engineering and Regenerative Medicine. 2010;7(2):248-54. | In vitro study |
| Lee YC, Yang SF, Hwang YF, Chueh LH, Chung KH. Microleakage of endodontic temporary restorative materials. Journal of Endodontics. 1993;19(10):516-20. | In vitro study |
| Leites AB, Baldissera EZ, Silva AF, Tarquinio S, Botero T, Piva E, et al. Histologic Response and Tenascin and Fibronectin Expression After Pulp Capping in Pig Primary Teeth With Mineral Trioxide Aggregate or Calcium Hydroxide. Operative Dentistry. 2011;36(4):448-56. | Animal study |
| Leksell E, Ridell K, Cvek M, Mejare I. Pulp exposure after stepwise versus direct complete excavation of deep carious lesions in young posterior permanent teeth. Endodontics and Dental Traumatology. 1996;12(4):192-6. | Permanent teeth |
| Lele GS, Subba Reddy VV. Comparison of antibacterial efficacy of intracanal medicaments in multiple visit pulpectomies in primary molars-an in vivo study. Journal of Indian Society of Pedodontics and Preventive Dentistry. 2010;28(1):18-24. | Bacteriology |
| Leonardo MR, Dasilva LAB, Utrilla LS, Leonardo RD, Consolaro A. EFFECT OF INTRACANAL DRESSINGS ON REPAIR AND APICAL BRIDGING OF TEETH WITH INCOMPLETE ROOT-FORMATION. Endodontics & Dental Traumatology. 1993;9(1):25-30. | Permanent teeth |
| Levin L, Coval M, Geiger SB. Cross-sectional radiographic survey of amalgam and resin-based composite posterior restorations. Quintessence International. 2007;38(6):511-4. | Survey |
| Li AS. Comparative study on curative effect of vitapex and root canal paste. Chinese Journal of Primary Medicine and Pharmacy. 2002;9(10):899-900. | Permanent teeth |
| Li J, Liu H, Ge LH. [Study on dental pulp stem cells from patients with hypophosphatasia]. [Chinese]. Beijing da Xue Xue Bao. 2009;Yi Xue Ban/Journal of Peking University. Health Sciences. 41(1):66-70. | Permanent teeth |
| Li S, Xu M. Comparison of mineral trioxide aggregate and Vitapex paste in the treatment of permanent teeth apexification. Journal of Clinical Rehabilitative Tissue Engineering Research. 2009;13(47):9381-4. | Permanent teeth |
| Liberman R, Ben-Amar A, Frayberg E, Abramovitz I, Metzger Z. Effect of repeated vertical loads on microleakage of IRM and calcium sulfate-based temporary fillings. Journal of Endodontics. 2001;27(12):724-9. | In vitro study |
| Libotte M. [Treatment of the dentition of the 7-year-old child]. [French]. Acta Stomatologica Belgica. 1967;64(1):71-98. | Biomaterials not compared |
| Lilly J, Vincent S, et al. 10% acyclovir in 80% dimethyl sulfoxide for the treatment of recurrent herpes labial lesions: A double-blind placebo-controlled study [abstract]. Oral Surgery, Oral Medicine, Oral Pathology, Oral Radiology, and Endodontics. 1995;80(4):423. | General medical problem |
| Lim MY, Lum SO, Poh RS, Lee GP, Lim KC. An in vitro comparison of the bleaching efficacy of 35% carbamide peroxide with established intracoronal bleaching agents. International Endodontic Journal. 2004;37(7):483-8. | In vitro study |
| Lindeboom JA, Frenken JW, Valkenburg P, van den Akker HP. The role of preoperative prophylactic antibiotic administration in periapical endodontic surgery: a randomized, prospective double-blind placebo-controlled study. International endodontic journal. 2005;38(12):877-81. | Biomaterials not compared |
| Lindemann M, Reader A, Nusstein J, Drum M, Beck M. Effect of sublingual triazolam on the success of inferior alveolar nerve block in patients with irreversible pulpitis. Journal of Endodontics. 2008;34(10):1167-70. | Anaesthesia |
| Lisson SN, Banham PW, Mendham NJ. Studies of fibre hemp and flax pulps as a feedstock for Australian newsprint production. Appita Journal. 2001;54(5):449-56. | Irrelevant |
| Lisson SN, Mendham NJ, Carberry PS. Development of a hemp (Cannabis sativa L.) simulation model 1. General introduction and the effect of temperature on the pre-emergent development of hemp. Australian Journal of Experimental Agriculture. 2000;40(3):405-11. | Irrelevant |
| Liu JF. Nd : YAG laser pulpotomy of human primary teeth. Lasers in Dentistry, Proceedings: Revolution of Dental Treatment in the New Millennium. 2003:251-6. | Abstract only |
| Liu JF. Effects of Nd:YAG laser pulpotomy on human primary molars. Journal of Endodontics. 2006;32(5):404-7. | No RCT |
| Liu Y, Li J, Hu J, Zhu S, Luo E, Hsu Y. Autogenous coronoid process pedicled on temporal muscle grafts for reconstruction of the mandible condylar in patients with temporomandibular joint ankylosis. Oral Surgery, Oral Medicine, Oral Pathology, Oral Radiology, and Endodontics. 2010;109(2):203-10. | Irrelevant |
| Lockhart PB, Brennan MT, Kent ML, Fox PC. Impact of amoxicillin on the incidence nature and duration of dental bacteremia [abstract]. Oral Surgery, Oral Medicine, Oral Pathology, Oral Radiology, and Endodontics. 2002;93(4):415. | Oral antibiotics |
| Loh A, O'Hoy P, Tran X, Charles R, Hughes A, Kubo K, et al. Evidence-based assessment: evaluation of the formocresol versus ferric sulfate primary molar pulpotomy. Pediatric Dentistry. 2004;26(5):401-9. | Review |
| Loo WTY, Dou YD, Chou WKJ, Wang M. Thymosin alpha 1 provides short-term and long-term benefits in the reimplantation of avulsed teeth: a double-blind randomized control pilot study. American Journal of Emergency Medicine. 2008;26(5):574-7. | Surgery |
| Louw AJ, Sarvan I, Chikte UM, Honkala E. One-year evaluation of atraumatic restorative treatment and minimum intervention techniques on primary teeth. SADJ : journal of the South African Dental Association = tydskrif van die Suid-Afrikaanse Tandheelkundige Vereniging. 2002;57(9):366-71. | Restorative dentistry |
| Loyola-Rodriguez JP, Zavala-Alonso V, Gonzalez-Alvarez CL, Juarez-Lopez LA, Patino-Marin N, Gonzalez CD. Dental treatment under general anesthesia in healthy and medically compromised developmentally disabled children: a comparative study. Journal of Clinical Pediatric Dentistry. 2009;34(2):177-82. | General medical problem |
| Lucas JSA, Grimshaw KEC, Collins K, Warner JO, Hourihane JO. Kiwi fruit is a significant allergen and is associated with differing patterns of reactivity in children and adults. Clinical and Experimental Allergy. 2004;34(7):1115-21. | Irrelevant |
| Lutfi AN, Kannan TP, Fazliah MN, Jamaruddin MA, Saidi J. Proliferative activity of cells from remaining dental pulp in response to treatment with dental materials. Australian Dental Journal. 2010;55(1):79-85. | Biological study |
| Lutrick DJ, Roehne N, Bacca M, Tech Assoc P, Paper IND. Pulping, black liquor and papermaking characteristics of plantation grown E-Camaldulensis and E-Viminalis. 1997 Pulping Conference, Books 1 and 2. 1997:635-51. | Irrelevant |
| Lux HC, Goetz F, Hellwig E. Case report: endodontic and surgical treatment of an upper central incisor with external root resorption and radicular cyst following a traumatic tooth avulsion. Oral Surgery Oral Medicine Oral Pathology Oral Radiology and Endodontology. 2010;110(5):E61-E7. | Case report |
| Lygidakis NA, Marinou D, Katsaris N. Analysis of dental emergencies presenting to a community paediatric dentistry centre. International Journal of Paediatric Dentistry. 1998;8(3):181-90. | Biomaterials not compared |
| Lyroudia K, Pantelidou O, Mikrogeorgis G, Chatzikallinikidis C, Nikopoulos N, Pitas I. The use of 3D computerized reconstruction for the study of coronal microleakage. International Endodontic Journal. 2000;33(3):243-7. | In vitro study |
| Ma Q, Hu X, Yu P. [Studies on aging enzyme activities of the human dental pulp blood vessels]. [Chinese]. Chung-Hua Kou Chiang i Hsueh Tsa Chih Chinese Journal of Stomatology. 1997;32(2):81-3. | In vitro study |
| Machiulskiene V, Richards A, Nyvad B, Baelum V. Prospective study of the effect of post-brushing rinsing behaviour on dental caries. Caries Research. 2002;36(5):301-7. | Biomaterials not compared |
| Mackie IC, Hill FJ, Worthington HV. Comparison of two calcium hydroxide pastes used for endodontic treatment of non-vital immature incisor teeth. Endodontics & Dental Traumatology. 1994;10(2):88-90. | Permanent teeth |
| Macnab I. Cervical spondylosis. Clinical Orthopaedics & Related Research. 1975(109):69-77. | Irrelevant |
| Madarati A, Rekab MS, Watts DC, Qualtrough A. Time-dependence of coronal seal of temporary materials used in endodontics. Australian Endodontic Journal: the Journal of the Australian Society of Endodontology. 2008;34(3):89-93. | Permanent teeth |
| Mahajan SK, Sidhu SS. Effect of fluoride on root resorption of autogenous dental replants. Clinical study. Australian Dental Journal. 1981;26(1):42-5. | Permanent teeth |
| Majcherczyk A, Johannes C, Huttermann A. Oxidation of aromatic alcohols by laccase from Trametes versicolor mediated by the 2,2 '-azino-bis-(3-ethylbenzothiazoline-6-sulphonic acid) cation radical and dication. Applied Microbiology and Biotechnology. 1999;51(2):267-76. | Irrelevant |
| Maltz M, Alves LS, Jardim JJ, Moura MD, de Oliveira EF. Incomplete caries removal in deep lesions: A 10-year prospective study. American Journal of Dentistry. 2011;24(4):211-4. | Biomaterials not compared |
| Mandari GJ, Truin GJ, van't Hof MA, Frencken JE. Effectiveness of three minimal intervention approaches for managing dental caries: survival of restorations after 2 years. Caries Research. 2001;35(2):90-4. | Permanent teeth |
| Mani SA, Chawla HS, Goel A. znOE or Ca(OH)2 as root canal filling material in primary teeth [abstract]. International Journal of Paediatric Dentistry. 1999;9(Suppl 1):113, Abstract no: P6.28. | No RCT |
| Mannocci F, Ferrari M, Watson TF. Microleakage of endodontically treated teeth restored with fiber posts and composite cores after cyclic loading: a confocal microscopic study. Journal of Prosthetic Dentistry. 2001;85(3):284-91. | In vitro study |
| Manzur A, Gonzalez AM, Pozos A, Silva-Herzog D, Friedman S. Bacterial quantification in teeth with apical periodontitis related to instrumentation and different intracanal medications: a randomized clinical trial. Journal of Endodontics. 2007;33(2):114-8. | Permanent teeth |
| Mao LS, Law K, Claude D, Francois B. Effects of carboxyl content on the characteristics of TMP long fibers. Industrial & Engineering Chemistry Research. 2008;47(11):3809-12. | Irrelevant |
| Marchenko AI, Likhota TF, Ruban AI, Sokolovskaia E, Levitskaia EV. [Use of antianaerobic agents in treating periodontitis in children]. [Russian]. Stomatologiia. 1986;65(3):67-8. | Periodontology |
| Marchi JJ, de Araujo FB, Froner AM, Straffon LH, Nor JE. Indirect pulp capping in the primary dentition: a 4 year follow-up study. Journal of Clinical Pediatric Dentistry. 2006;31(2):68-71. | Indirect pulp capping only |
| Marchi JJ, Froner AM, Alves HL, Bergmann CP, Araujo FB. Analysis of primary tooth dentin after indirect pulp capping. Journal of Dentistry for Children (Chicago, Ill. 2008). 75(3):295-300. | In vitro study |
| Marks LA, Faict N, Welbury RR. Literature review: Restorations of class II cavities in the primary dentition with compomers. [Review]. European Archives of Paediatric Dentistry: Official Journal of the European Academy of Paediatric Dentistry. 2010;11(3):109-14. | Review |
| Marks LA, van Amerongen WE, Borgmeijer PJ, Groen HJ, Martens LC. Ketac Molar Versus Dyract Class II restorations in primary molars: twelve month clinical results. ASDC journal of dentistry for children. 2000;67(1):37-41, 8-419. | Restorative dentistry |
| Marks LA, van Amerongen WE, Kreulen CM, Weerheijm KL, Martens LC. Conservative interproximal box-only polyacid modified composite restorations in primary molars, twelve-month clinical results. ASDC journal of dentistry for children. 1999;66(1):23-9, 12. | Restorative dentistry |
| Marks LA, Weerheijm KL, van Amerongen WE, Groen HJ, Martens LC. Dyract versus Tytin Class II restorations in primary molars: 36 months evaluation. Caries research. 1999;33(5):387-92. | Restorative dentistry |
| Marques-Ferreira M, Rabaca-Botelho MF, Carvalho L, Oliveiros B, Palmeirao-Carrilho EV. Autogenous tooth transplantation: Evaluation of pulp tissue regeneration. Medicina Oral, Patologia Oral y Cirugia Bucal. 2011;16(7):e984-e9. | Surgery |
| Martin M, Nusstein J, Drum M, Reader A, Beck M. Anesthetic efficacy of 1.8 mL versus 3.6 mL of 4% articaine with 1:100,000 epinephrine as a primary buccal infiltration of the mandibular first molar. Journal of Endodontics. 2011;37(5):588-92. | Anesthesia |
| Massler M. Preventive endodontics: vital pulp therapy. Journal of the Dental Association of South Africa. 1968;23(1):208-15. | No RCT |
| Mathias S, Koerber A, Fadavi S, Punwani I. Specialty and sex as predictors of depression in dentists. Journal of the American Dental Association. 2005;136(10):1388-95. | Epidemiologic study |
| Mazhari F, Gharaghahi M. Effect of thickness of cavity wall on fracture strength of pulpotomized primary molar teeth with class capital PE, Cyrillic amalgam restorations. European Archives of Paediatric Dentistry: Official Journal of the European Academy of Paediatric Dentistry. 2008;9(1):31-6. | In vitro study |
| McDonald NJ, Friedberg BH, Dumsha TC, Hovland EJ, et al. An in vivo evaluation of temporary endodontic restorative material (T.E.R.M) [abstract]. Journal of Dental Research. 1988;67(Spec Iss):313, Abstract no: 1600. | Permanent teeth |
| McDougal RA, Delano EO, Caplan D, Sigurdsson A, Trope M. Success of an alternative for interim management of irreversible pulpitis. Journal of the American Dental Association. 2004;135(12):1707-12. | Permanent teeth |
| McEntire M, Nusstein J, Drum M, Reader A, Beck M. Anesthetic efficacy of 4% Articaine with 1:100,000 epinephrine versus 4% articaine with 1:200,000 epinephrine as a primary buccal infiltration in the mandibular first molar. Journal of Endodontics. 2011;37(4):450-4. | Anesthesia |
| McWhorter AG. Treatment planning for the pediatric patient. Texas Dental Journal. 2010;127(12):1275-80. | Review |
| Mehl A, Folwaczny M, Haffner C, Hickel R. Bactericidal effects of 2.94 microns Er:YAG-laser radiation in dental root canals. Journal of Endodontics. 1999;25(7):490-3. | Biological study |
| Mejare I. Pulpotomy of primary molars with coronal or total pulpitis using formocresol technique. Scandinavian Journal of Dental Research. 1979;87(3):208-16. | Restorative dentistry |
| Mejare I, Cvek M. Partial pulpotomy in young permanent teeth with deep carious lesions. Endodontics & Dental Traumatology. 1993;9(6):238-42. | Permanent teeth |
| Melczer K. [Advantages of the N2 root canal filling material in the treatment of pulpitis in deciduous molars]. [Hungarian]. Fogorvosi Szemle. 1972;65(11):348-50. | Translation problem |
| Melton D, Cobb S, Krell KV. A comparison of two temporary restorations: light-cured resin versus a self-polymerizing temporary restoration. Oral Surgery, Oral Medicine, Oral Pathology. 1990;70(2):221-5. | Restorative dentistry |
| Mendoza AM, Reina JE, Garcia-Godoy F. Evolution and prognosis of necrotic primary teeth after pulpectomy. American Journal of Dentistry. 2010;23(5):265-8. | No RCT |
| Mente J, Geletneky B, Ohle M, Koch MJ, Friedrich Ding PG, Wolff D, et al. Mineral trioxide aggregate or calcium hydroxide direct pulp capping: an analysis of the clinical treatment outcome. Journal of Endodontics. 2010;36(5):806-13. | Retrospective study |
| Meon R. Review of currently used medicaments in vital pulpotomy. [Review] [31 refs]. Dental Journal of Malaysia. 1987;9(2):23-7. | Review |
| Meon R. Management of an avulsed tooth with severe root resorption. Singapore Dental Journal. 1988;13(1):53-6. | Surgery |
| Merglova V. The treatment of non-vital immature permanent teeth by filling of root canals with calcium hydroxide. European Journal of Paediatric Dentistry. 2000;2(1):38. | Permanent teeth |
| Merlen JF. [Thrombophlebitis migrans]. [French]. Phlebologie. 1977;30(2):127-32. | Irrelevant |
| Mertz-Fairhurst EJ, Curtis Jr JW, Ergle JW, Rueggeberg FA, Adair SM. Ultraconservative and cariostatic sealed restorations: results at year 10. Journal of the American Dental Association (1939). 1998;129(1):55-66. | Restorative dentistry |
| Messer LB, Cline JT, Korf NW. Long term effects of primary molar pulpotomies on succedaneous bicuspids. Journal of Dental Research. 1980;59(2):116-23. | Biomaterials not compared |
| Mhaville RJ, van Amerongen WE, Mandari GJ. Residual caries and marginal integrity in relation to Class II glass ionomer restorations in primary molars. European archives of paediatric dentistry : official journal of the European Academy of Paediatric Dentistry. 2006;7(2):81-4. | Restorative dentistry |
| Mikhailovskaia VP, Khariton VS, Ostromentskaia TK, Fraint IP. [Use of paraformaldehyde in treating pulpitis of the deciduous teeth in children]. [Russian]. Stomatologiia. 1982;61(6):26-7. | Translation problem |
| Millns B, Martin MV, Williams MC. Raised salivary endotoxin concentration as a predictor of infection in pediatric leukemia patients. Oral Surgery, Oral Medicine, Oral Pathology, Oral Radiology, and Endodontics. 1999;88(1):50-5. | General medical problem |
| Minagawa M. [Clinico-pathological studies on the effects of calcium hydroxide eugenol preparations to the human vital pulp tissues]. [Japanese]. Shikwa Gakuho. 1989;89(5):889-930. | Permanent teeth |
| Mitic A, Mitic N, Zivkovic S, Tosic G, Savic V, Dacic S, et al. [Efficiency of final irrigation of root canal in removal of smear layer]. [Serbian]. Srpski Arhiv Za Celokupno Lekarstvo. 2009;137(9-10):482-9. | Permanent teeth |
| Mohamed N. A comparison of two liner materials for use in the ferric sulfate pulpotomy. SADJ. 2008;63(6):338. | Restorative dentistry |
| Mohammadi Z, Shahriari S. Residual antibacterial activity of chlorhexidine and MTAD in human root dentin in vitro. Journal of Oral Science. 2008;50(1):63-7. | In vitro study |
| Moore A, Howley MF, O'Connell AC. Treatment of open apex teeth using two types of white mineral trioxide aggregate after initial dressing with calcium hydroxide in children. Dental Traumatology. 2011;27(3):166-73. | Permanent teeth |
| Moore PA, Finder RL, Jackson DL. Multidrug intravenous sedation: determinants of the sedative dose of midazolam. Oral Surgery, Oral Medicine, Oral Pathology, Oral Radiology, and Endodontics. 1997;84(1):5-10. | Anesthesia |
| Mor C, Rotstein I, Friedman S. Incidence of interappointment emergency associated with endodontic therapy. Journal of Endodontics. 1992;18(10):509-11. | Permanent teeth |
| Morand MA. [Study on the causes of post-endodontic failures: a descriptive analysis of 198 cases of reoperation]. [French]. Journal (Canadian Dental Association). 1990;56(10):927-32. | Permanent teeth |
| Morand MA. [The causes of post-endodontic failure: a descriptive analysis of a population of 198 cases of surgical retreatment]. [French]. Journal (Canadian Dental Association). 1990;56(6):491-6. | Permanent teeth |
| Moritz A, Schoop U, Goharkhay K, Sperr W. Advantages of a pulsed CO2 laser in direct pulp capping: a long-term in vivo study. Lasers in Surgery & Medicine. 1998;22(5):288-93. | Permanent teeth |
| Moritz A, Schoop U, Goharkhay K, Sperr W. The CO2 laser as an aid in direct pulp capping. Journal of Endodontics. 1998;24(4):248-51. | Permanent teeth |
| Morse DR, Furst ML, Belott RM, Lefkowitz RD, Spritzer IB, Sideman BH. A prospective randomized trial comparing periapical instrumentation to intracanal instrumentation in cases of asymptomatic pulpal-periapical lesions. Oral Surgery, Oral Medicine, Oral Pathology. 1987;64(6):734-41. | Permanent teeth |
| Morse DR, Furst ML, Belott RM, Lefkowitz RD, Spritzer IB, Sideman BH. Infectious flare-ups and serious sequelae following endodontic treatment: a prospective randomized trial on efficacy of antibiotic prophylaxis in cases of asymptomatic pulpal-periapical lesions. Oral Surgery, Oral Medicine, Oral Pathology. 1987;64(1):96-109. | Permanent teeth |
| Morse DR, Furst ML, Lefkowitz RD, D'Angelo D, Esposito JV. A comparison of erythromycin and cefadroxil in the prevention of flare-ups from asymptomatic teeth with pulpal necrosis and associated periapical pathosis. Oral Surgery, Oral Medicine, Oral Pathology. 1990;69(5):619-30. | Permanent teeth |
| Moskovitz M, Sammara E, Holan G. Success rate of root canal treatment in primary molars. Journal of Dentistry. 2005;33(1):41-7. | No RCT |
| Moura MD, Haddad JP, Senna MI, Ferreira E, Mesquita RA. A new topical treatment protocol for oral hairy leukoplakia. Oral Surgery, Oral Medicine, Oral Pathology, Oral Radiology, and Endodontics. 2010;110(5):611-7. | General medical problem |
| Mousavinasab M, Namazikhah MS, Sarabi N, Jajarm HH, Bidar M, Ghavamnasiri M. Histopathology study on pulp response to glass ionomers in human teeth. Journal of the California Dental Association. 2008;36(1):51-5. | In vitro study |
| Mulder GR, van Amerongen WE, Vingerling PA. Consequences of endodontic treatment of primary teeth. Part II: a clinical investigation into the influence of formocresol pulpotomy on the permanent successor. ASDC J Dent Child. 1987;54(1):35-9. | Biomaterials not compared |
| Muniz MA, Keszler A, Dominguez FV. The formocresol technique in young permanent teeth. A histopathologic study. Oral Surgery, Oral Medicine, Oral Pathology. 1983;55(6):611-21. | Permanent teeth |
| Munshi AK, Hegde AM, Girdhar D. Clinical evaluation of electronic dental anesthesia for various procedures in pediatric dentistry. Journal of Clinical Pediatric Dentistry. 2000;24(3):199-204. | Anaesthesia |
| Murad C, Fariniuk LF, Fidel S, Fidel RA, Sassone LM. Bacterial leakage in root canals filled with calcium hydroxide paste associated with different vehicles. Brazilian Dental Journal. 2008;19(3):232-7. | In vitro study |
| Murali Mohan S, Kaushik SK. Root canal treatment using thermoplasticized carrier condensation technique. Medical Journal Armed Forces India. 2009;65(4):336-41. | Permanent teeth |
| Murray PE, About I, Lumley PJ, Franquin JC, Windsor LJ, Smith AJ. Odontoblast morphology and dental repair. Journal of dentistry. 2003;31(1):75-82. | Anatomy study |
| Murray PE, Farber RM, Namerow KN, Kuttler S, Garcia-Godoy F. Evaluation of Morinda citrifolia as an endodontic irrigant. Journal of Endodontics. 2008;34(1):66-70. | Biological study |
| Nadin G, Goel BR, Yeung CA, Glenny AM. Pulp treatment for extensive decay in primary teeth. [Review] [99 refs]. Cochrane Database of Systematic Reviews. 2003(1):CD003220. | Review |
| Nagaratna PJ, Shashikiran ND, Subbareddy VV. In vitro comparison of NiTi rotary instruments and stainless steel hand instruments in root canal preparations of primary and permanent molar. Journal of the Indian Society of Pedodontics & Preventive Dentistry. 2006;24(4):186-91. | In vitro study |
| Naidu S, Loughlin P, Coldwell SE, Noonan CJ, Milgrom P. A randomized controlled trial comparing mandibular local anesthesia techniques in children receiving nitrous oxide-oxygen sedation. Anesthesia Progress. 2004;51(1):19-23. | Anesthesia |
| Nair PNR, Duncan HF, Ford TRP, Luder HU. Histological, ultrastructural and quantitative investigations on the response of healthy human pulps to experimental capping with mineral trioxide aggregate: a randomized controlled trial. International Endodontic Journal. 2008;41(2):128-50. | Permanent teeth |
| Nair PNR, Duncan HF, Pitt Ford TR, Luder HU. Histological, ultrastructural and quantitative investigations on the response of healthy human pulps to experimental capping with Mineral Trioxide Aggregate: a randomized controlled trial (Reprinted). International Endodontic Journal. 2009;42(5):422-44. | Permanent teeth |
| Nakai TR, Peterson JC, Jr., Law DB. Current concepts in the management of the hemophilic pedodontic patient. Journal of Dentistry for Children. 1974;41(5):361-6. | General medical problem |
| Nakashima M, Nobuke H, Miyake Y, Nagasaka N. Radiographic follow up examination on pulpotomy in primary teeth. Treatment with formocresol paste. Shoni shikagaku zasshi. 1989;The Japanese journal of pedodontics. 27(2):537-45. | Biomaterials not compared |
| Nayyar S, Tewari S, Arora B. Comparison of human pulp response to total-etch and self-etch bonding agents. Oral Surgery, Oral Medicine, Oral Pathology, Oral Radiology, and Endodontics. 2007;104(2):e45-52. | Permanent teeth |
| Newcomb BE, Clark SJ, Eleazer PD. Degradation of the sealing properties of a zinc oxide-calcium sulfate-based temporary filling material by entrapped cotton fibers. Journal of Endodontics. 2001;27(12):789-90. | Permanent teeth |
| Ng FK, Messer LB. Mineral trioxide aggregate as a pulpotomy medicament: an evidence-based assessment. [Review] [81 refs]. European Archives of Paediatric Dentistry: Official Journal of the European Academy of Paediatric Dentistry. 2008;9(2):58-73. | Review |
| Ng YL, Mann V, Gulabivala K. Outcome of secondary root canal treatment: a systematic review of the literature. International Endodontic Journal. 2008;41(12):1026-46. | Review |
| Ng YL, Mann V, Gulabivala K. Tooth survival following non-surgical root canal treatment: a systematic review of the literature. International Endodontic Journal. 2010;43(3):171-89. | Review |
| Ng YL, Mann V, Rahbaran S, Lewsey J, Gulabivala K. Outcome of primary root canal treatment: systematic review of the literature - part 1. Effects of study characteristics on probability of success. [Review] [148 refs]. International Endodontic Journal. 2007;40(12):921-39. | Review |
| Ng YL, Mann V, Rahbaran S, Lewsey J, Gulabivala K. Outcome of primary root canal treatment: systematic review of the literature - Part 2. Influence of clinical factors. International Endodontic Journal. 2008;41(1):6-31. | Review |
| Nguyen PM, Kenny DJ, Barrett EJ. Socio-economic burden of permanent incisor replantation on children and parents. Dental Traumatology. 2004;20(3):123-33. | Permanent incisor |
| Nickenig HJ, Spiekermann H, Wichmann M, Andreas SK, Eitner S. Survival and complication rates of combined tooth-implant-supported fixed and removable partial dentures. International Journal of Prosthodontics. 2008;21(2):131-7. | Prosthodontics |
| Nihtila A, Widstrom E, Elonheimo O. Heavy consumption of dental services among Finnish adults. Community Dental Health. 2010;27(4):227-32. | Adults |
| Noguera AP, McDonald NJ. Comparative in vitro coronal microleakage study of new endodontic restorative materials. Journal of Endodontics. 1990;16(11):523-7. | In vitro study |
| Nordstrom DO, Wei SH, Johnson R. Use of stannous fluoride for indirect pulp capping. Journal of the American Dental Association. 1974;88(5):997-1003. | Indirect pulp capping only |
| Nothdurft FP, Schmitt T, Motter PJ, Pospiech PR. Influence of fatigue testing and cementation mode on the load-bearing capability of bovine incisors restored with crowns and zirconium dioxide posts. Clinical Oral Investigations. 2008;12(4):331-6. | Animal study |
| Ntima-Nsiemi K, Mbuila C, Bascoulard M, Lemasson P, Abbar H. [Sickle cell anemia patients in oral medicine. What treatment? Apropos of a case and review of the literature]. [French]. Revue de Stomatologie et de Chirurgie Maxillo-Faciale. 1998;98(6):382-6. | Irrelevant |
| Nusstein J, Berlin J, Reader A, Beck M, Weaver JM. Comparison of injection pain, heart rate increase, and postinjection pain of articaine and lidocaine in a primary intraligamentary injection administered with a computer-controlled local anesthetic delivery system. Anesthesia Progress. 2004;51(4):126-33. | Anesthesia |
| Nusstein J, Burns Y, Reader A, Beck M, Weaver J. Injection pain and postinjection pain of the palatal-anterior superior alveolar injection, administered with the Wand Plus system, comparing 2% lidocaine with 1:100,000 epinephrine to 3% mepivacaine. Oral Surgery, Oral Medicine, Oral Pathology, Oral Radiology, and Endodontics. 2004;97(2):164-72. | Anesthesia |
| Nusstein J, Lee S, Reader A, Beck M, Weaver J. Injection pain and postinjection pain of the anterior middle superior alveolar injection administered with the Wand or conventional syringe. Oral Surgery, Oral Medicine, Oral Pathology, Oral Radiology, and Endodontics. 2004;98(1):124-31. | Anesthesia |
| Ochoa-Romero T, Mendez-Gonzalez V, Flores-Reyes H, Pozos-Guillen AJ. Comparison between rotary and manual techniques on duration of instrumentation and obturation times in primary teeth. Journal of Clinical Pediatric Dentistry. 2011;35(4):359-63. | Biomaterials not compared |
| Odabas ME, Bodur H, Baris E, Demir C. Clinical, radiographic, and histopathologic evaluation of Nd:YAG laser pulpotomy on human primary teeth. Journal of Endodontics. 2007;33(4):415-21. | No RCT |
| Odabas ME, Cinar C, Tulunoglu O, Isik B. A New Haemostatic Agent's Effect on the Success of Calcium Hydroxide Pulpotomy in Primary Molars. Pediatric Dentistry. 2011;33(7):529-34. | Biomaterials not compared |
| Odabas ME, Tulunoglu O, Ozalp SO, Bodur H. Microleakage of different temporary filling materials in primary teeth. Journal of Clinical Pediatric Dentistry. 2009;34(2):157-60. | In vitro study |
| OdrÌa R, Castillo R. Comparison of the efficacy of the non-vital formocresol pulpotomy combined with a quirurgic curettement of the furcation area vs. the non-vital formocresol pulpotomy without quirurgic curettement of the furcation for fistulited primary molars [abstract]. Journal of Dental Research. 1992;71(4 [Divisional Abstracts]):1080, Abstract no: O-18. | Curettement associated with pulpotomy evaluated |
| Ogura Y, Katsuumi I. Setting properties and sealing ability of hydraulic temporary sealing materials. Dental Materials Journal. 2008;27(5):730-5. | Permanent teeth |
| Olender D, Wild P, Byrnes P, Ouellet D, Sabourin M. Forces on bars in high-consistency mill-scale refiners: Effect of consistency. Nordic Pulp & Paper Research Journal. 2008;23(2):218-23. | Irrelevant |
| Oliveira C, Dias PF, dos Santos MPA, Maia LC. Split mouth randomized controlled clinical trial of beveled cavity preparations in primary molars: an 18-Month follow up. Journal of Dentistry. 2008;36(9):754-8. | Restorative dentistry |
| Olivi G, Genovese MD, Maturo P, Docimo R. Pulp capping: advantages of using laser technology. European Journal of Paediatric Dentistry. 2007;8(2):89-95. | Permanent teeth |
| Olmez A, Tuna D, Ozdogan YT, Ulker AE. The effectiveness of different thickness of mineral trioxide aggregate on coronal leakage in endodontically treated deciduous teeth. Journal of Dentistry for Children (Chicago, Ill. 2008). 75(3):260-3. | In vitro study |
| Olsson H, Davies JR, Holst KE, Schroder U, Petersson K. Dental pulp capping: effect of Emdogain Gel on experimentally exposed human pulps. International Endodontic Journal. 2005;38(3):186-94. | Experimental study |
| Onçag O, Cogulu D, Gogulu D, Uzel A. Efficacy of various intracanal medicaments against Enterococcus faecalis in primary teeth: an in vivo study. The Journal of clinical pediatric dentistry. 2006;30(3):233-7. | Bacteriology |
| Ong KS, Seymour RA, Tan JM. A prospective randomized crossover study of the preemptive analgesic effect of nitrous oxide in oral surgery. Oral Surgery, Oral Medicine, Oral Pathology, Oral Radiology, and Endodontics. 2004;98(6):637-42. | Anesthesia |
| Orhan AI, Oz FT, Orhan K. Pulp exposure occurrence and outcomes after 1- or 2-visit indirect pulp therapy vs complete caries removal in primary and permanent molars. Pediatric Dentistry. 2010;32(4):347-55. | Biomaterials not compared |
| Orhan AI, Oz FT, Ozcelik B, Orhan K. A clinical and microbiological comparative study of deep carious lesion treatment in deciduous and young permanent molars. Clinical Oral Investigations. 2008;12(4):369-78. | Biomaterials not compared |
| Orihel TC, Eberhard ML. Loa loa: development and course of patency in experimentally-infected primates. Tropical Medicine & Parasitology. 1985;36(4):215-24. | Animal study |
| Orstavik D, Qvist V, Stoltze K. A multivariate analysis of the outcome of endodontic treatment. European Journal of Oral Sciences. 2004;112(3):224-30. | Permanent teeth |
| Oulis CJ, Vadiakas GP, Vasilopoulou A. The effectiveness of mandibular infiltration compared to mandibular block anesthesia in treating primary molars in children. Pediatric Dentistry. 1996;18(4):301-5. | Anesthesia |
| Overdevest G, Vleggeert-Lankamp C, Luijsterburg P, Brand R, Eekhof J, Westendorp R, et al. (Cost) effectiveness of surgery versus prolonged conservative treatment in lumbar stenosis: Design of a randomized controlled trial. Osteoarthritis and Cartilage Conference: 2010 Osteoarthritis Research Society International, OARSI World Congress Brussels Belgium Conference Start: 20100923 Conference End: 20100926. 2010;18:S230-1. | Irrelevant |
| Ozbek SM, Ozbek A, Erdogan AS. ANALYSIS OF ENTEROCOCCUS FAECALIS IN SAMPLES FROM TURKISH PATIENTS WITH PRIMARY ENDODONTIC INFECTIONS AND FAILED ENDODONTIC TREATMENT BY REAL-TIME PCR SYBR GREEN METHOD. Journal of Applied Oral Science. 2009;17(5):370-4. | In vitro study |
| Oztas N, Ulusu T, Bodur H, Dogan C. The wand in pulp therapy: an alternative to inferior alveolar nerve block. Quintessence International. 2005;36(7-8):559-64. | Anesthesia |
| Pacios MG, de la Casa ML, de los Angeles Bulacio M, Lopez ME. Calcium hydroxide's association with different vehicles: In vitro action on some dentinal components. Oral Surgery Oral Medicine Oral Pathology Oral Radiology & Endodontics. 2003;96(1):96-101. | In vitro study |
| Pai SF, Yang SF, Sue WL, Chueh LH, Rivera EM. Microleakage between endodontic temporary restorative materials placed at different times. Journal of Endodontics. 1999;25(6):453-6. | In vitro study |
| Pandey RK, Padmanabhan MY, Saksena AK, Chandra G. Midazolam-fentanyl analgo-sedation in pediatric dental patients--a pilot study. Journal of Clinical Pediatric Dentistry. 2010;35(1):105-10. | Anesthesia |
| Pane ES, Messer HH. The quality of clinical fit of stainless steel bands used in endodontics. Australian Endodontic Journal: the Journal of the Australian Society of Endodontology. 2001;27(3):105-8. | Restorative dentistry |
| Pascon FM, Kantovitz KR, Borges AF, Puppin-Rontani RM. Effect of cleansers and irrigation methods on primary root dentin permeability. Journal of dentistry for children (Chicago, Ill. 2007). 74(1):30-5. | In vitro stusy |
| Pascon FM, Kantovitz KR, Caldo-Teixeira AS, Borges AFS, Silva TN, Puppin-Rontani RM, et al. Clinical evaluation of composite and compomer restorations in primary teeth: 24-month results. Journal of Dentistry. 2006;34(6):381-8. | Restorative dentistry |
| Pascon FM, Kantovitz KR, Sinhoreti MA, Puppin-Rontani RM. Is the presence of the smear layer a limiting factor for root dentin permeability in primary teeth? Journal of Dentistry for Children (Chicago, Ill. 2007). 74(3):182-8. | In vitro study |
| Passi S, Pandit IK, Srivastava N, Gugnani N, Gupta M. A comparative evaluation of the fracture strength of pulpotomized primary molars restored with various restorative materials. Journal of Clinical Pediatric Dentistry. 2007;31(3):164-6. | In vitro study |
| Paterson SA, Curzon ME. The effect of amoxycillin versus penicillin V in the treatment of acutely abscessed primary teeth. British Dental Journal. 1993;174(12):443-9. | Oral antibiotics |
| Pelcowa M. [Evaluation of a polyantibiotic mixture with hydrocortisone for direct covering of accidentally denuded dental pulp and in the treatment of pulpitis]. [Polish]. Folia Medica Cracoviensia. 1968;10(1):109-42. | Permanent teeth |
| Peltola JS. A PANORAMATOMOGRAPHIC STUDY OF THE TEETH AND JAWS OF FINNISH UNIVERSITY-STUDENTS. Community Dentistry and Oral Epidemiology. 1993;21(1):36-9. | Epidemiologic study |
| Peltola JS, Venta I, Haahtela S, Lakoma A, Ylipaavalniemi P, Turtola L. Dental and oral radiographic findings in first-year university students in 1982 and 2002 in Helsinki, Finland. Acta Odontologica Scandinavica. 2006;64(1):42-6. | Epidemiologic study |
| Penesis VA, Fitzgerald PI, Fayad MI, Wenckus CS, BeGole EA, Johnson BR. Outcome of one-visit and two-visit endodontic treatment of necrotic teeth with apical periodontitis: a randomized controlled trial with one-year evaluation. Journal of Endodontics. 2008;34(3):251-7. | Permanent teeth |
| Peng L, Ye L, Guo X, Tan H, Zhou X, Wang C, et al. Evaluation of formocresol versus ferric sulphate primary molar pulpotomy: a systematic review and meta-analysis. [Review] [49 refs]. International Endodontic Journal. 2007;40(10):751-7. | Review |
| Peng L, Ye L, Tan H, Zhou X. Evaluation of the formocresol versus mineral trioxide aggregate primary molar pulpotomy: a meta-analysis. Oral Surgery, Oral Medicine, Oral Pathology, Oral Radiology, and Endodontics. 2006;102(6):e40-e4. | Meta-analysis |
| Peppel P, Anton F. RESPONSES OF RAT MEDULLARY DORSAL HORN NEURONS FOLLOWING INTRANASAL NOXIOUS CHEMICAL-STIMULATION - EFFECTS OF STIMULUS-INTENSITY, DURATION, AND INTERSTIMULUS-INTERVAL. Journal of Neurophysiology. 1993;70(6):2260-75. | Irrelevant |
| Percinoto C, Castro AM, Pinto L. Clinical and radiographic assessment of pulpotomies in primary teeth using calcium hydroxide paste and mineral trioxide aggregate [abstract]. Journal of Dental Research. 2003;82(Spec Iss B):B-322, Abstract no: 2493. | Abstract only |
| Percinoto C, de Castro AM, Pinto LM. Clinical and radiographic evaluation of pulpotomies employing calcium hydroxide and trioxide mineral aggregate. General Dentistry. 2006;54(4):258-61. | No RCT |
| Peters DD, Baumgartner JC, Lorton L. Adult pulpal diagnosis. I. Evaluation of the positive and negative responses to cold and electrical pulp tests. Journal of Endodontics. 1994;20(10):506-11 | Adult |
| Peters MC, Bresciani E, Barata TJ, Fagundes TC, Navarro RL, Navarro MF, et al. In vivo dentin remineralization by calcium-phosphate cement. Journal of Dental Research. 2010;89(3):286-91. | In vitro study |
| Peters O, Gohring TN, Lutz F. Effect of eugenol-containing sealer on marginal adaptation of dentine-bonded resin fillings. International Endodontic Journal. 2000;33(1):53-9. | Permanent teeth |
| Peters OA, Barbakow F, Peters CI. An analysis of endodontic treatment with three nickel-titanium rotary root canal preparation techniques. International Endodontic Journal. 2004;37(12):849-59. | Permanent teeth |
| Pettiette M, Hupp J, Mesaros S, Trope M. Periodontal healing of extracted dogs' teeth air-dried for extended periods and soaked in various media. Endodontics & Dental Traumatology. 1997;13(3):113-8. | Animal study |
| Phaneuf RA, Frankl SN, Ruben MP. A comparative histological evaluation of three calcium hydroxide preparations on the human primary dental pulp. Journal of Dentistry for Children. 1968;35(1):61-76. | In vitro study |
| Philippe FX, Remience V, Dourmad JY, Cabaraux JF, Vandenheede M, Nicks B. Food fibers in gestating sows: effects on nutrition, behaviour, performances and waste in the environment. Productions Animales. 2008;21(3):277-90. | Irrelevant |
| Phillips RW, Hamilton AI, Jendresen MD, McHorris WH, Schallhorn RG. Report of Committee on Scientific Investigation of the American Academy of Restorative Dentistry. [Review] [415 refs][Erratum appears in J Prosthet Dent 1986 Oct;56(4):515]. Journal of Prosthetic Dentistry. 1986;55(6):736-72. | Review |
| Pichler JW, Beirne OR. Lingual flap retraction and prevention of lingual nerve damage associated with third molar surgery: a systematic review of the literature [review]. Oral Surgery, Oral Medicine, Oral Pathology, Oral Radiology, and Endodontics. 2001;91(4):395-401. | Review |
| Pieper CM, Zanchi CH, Rodrigues-Junior SA, Moraes RR, Pontes LS, Bueno M. Sealing ability, water sorption, solubility and toothbrushing abrasion resistance of temporary filling materials. International Endodontic Journal. 2009;42(10):893-9. | Permanent teeth |
| Pilo R, Cardash HS, Levin E, Assif D. Effect of core stiffness on the in vitro fracture of crowned, endodontically treated teeth. Journal of Prosthetic Dentistry. 2002;88(3):302-6. | In vitro study |
| Pinheiro SL, Oda M, Matson E, Duarte DA, Guedes-Pinto AC. Simultaneous activation technique: an alternative for bonding composite resin to glass ionomer. Pediatric dentistry. 2003;25(3):270-4. | Restorative dentistry |
| Pinto AS, de Araujo FB, Franzon R, Figueiredo MC, Henz S, Garcia-Godoy F, et al. Clinical and microbiological effect of calcium hydroxide protection in indirect pulp capping in primary teeth. American Journal of Dentistry. 2006;19(6):382-6. | Indirect pulp capping only |
| Pinto DN, de Sousa DL, Araujo RB, Moreira-Neto JJ. Eighteen-month clinical and radiographic evaluation of two root canal-filling materials in primary teeth with pulp necrosis secondary to trauma. Dental Traumatology. 2011;27(3):221-4. | Incisors |
| Poeschl PW, Crepaz V, Russmueller G, Seemann R, Hirschl AM, Ewers R. Endodontic Pathogens Causing Deep Neck Space Infections: Clinical Impact of Different Sampling Techniques and Antibiotic Susceptibility. Journal of Endodontics. 2011;37(9):1201-5. | Biomaterials not compared |
| Pohl Y, Krema M, Kirschner H. Interrelation between endodontic status, pathologic cemental granules and periodontal ligament adhering to the root of extracted teeth. Oral Surgery, Oral Medicine, Oral Pathology, Oral Radiology, and Endodontics. 2007;103(1):127-33. | Surgery |
| Pollick HF, Pawson IG, Martorell R, Mendoza FS. THE ESTIMATED COST OF TREATING UNMET DENTAL RESTORATIVE NEEDS OF MEXICAN-AMERICAN CHILDREN FROM SOUTHWESTERN UNITED-STATES-HHANES, 1982-83. Journal of Public Health Dentistry. 1991;51(4):195-204. | Cost |
| Ponova M. [Treatment of gangrene in deciduous teeth]. [Bulgarian]. Stomatologiia. 1973;55(2):146-9. | Translation problem |
| Poussa M, Remes V, Lamberg T, Tervahartiala P, Schlenzka D, Yrjonen T, et al. Treatment of severe spondylolisthesis in adolescence with reduction or fusion in situ: long-term clinical, radiologic, and functional outcome. Spine. 2006;31(5):583-90; discussion 91-2. | Irrelevant |
| Pozzobon MH, Vieira RD, Alves AMH, Reyes-Carmona J, Teixeira CS, de Souza BDM, et al. Assessment of pulp blood flow in primary and permanent teeth using pulse oximetry. Dental Traumatology. 2011;27(3):184-8. | Diagnostic study |
| Prabhakar A, Kiran NK. Clinical evaluation of polyamide polymer burs for selective carious dentin removal. The journal of contemporary dental practice. 2009;10(4):26-34. | Restorative dentistry |
| Prabhakar AR, Bedi S. Effect of glutaraldehyde and ferric sulfate on shear bond strength of adhesives to primary dentin. Journal of the Indian Society of Pedodontics & Preventive Dentistry. 2008;26(Suppl 3):S109-13. | Restorative dentistry |
| Pradhan DP, Chawla HS, Gauba K, Goyal A. Comparative evaluation of endodontic management of teeth with unformed apices with mineral trioxide aggregate and calcium hydroxide. Journal of Dentistry for Children (Chicago, Ill. 2006). 73(2):79-85. | Permanent teeth |
| Punwani I, Fadavi S. The Efficacy of PulpfixÆ Glutaraldehyde Pulpotomy Agent for the Treatment of Vital Primary Teeth with Carious Pulp Exposure (IADR Abstract). Journal of Dental Research. 1993;72(Special Issue IADR Abstracts):212 (Abs No 869). | Abstract only |
| Qudeimat MA, Al-Saiegh FA, Al-Omari Q, Omar R. Restorative treatment decisions for deep proximal carious lesions in primary molars. European archives of paediatric dentistry : official journal of the European Academy of Paediatric Dentistry. 2007;8(1):37-42. | Restorative dentistry |
| Qudeimat MA, Barrieshi-Nusair KM, Owais AI. Calcium hydroxide vs mineral trioxide aggregates for partial pulpotomy of permanent molars with deep caries. European Archives of Paediatric Dentistry: Official Journal of the European Academy of Paediatric Dentistry. 2007;8(2):99-104. | Permanent teeth |
| Quijano G, Siminovich M, Drut R. Histopathologic findings in the lymphoid and reticuloendothelial system in pediatric HIV infection: A postmortem study. Pediatric Pathology & Laboratory Medicine. 1997;17(6):845-56. | Irrelevant |
| Qvist V, Laurberg L, Poulsen A, Teglers PT. Class II restorations in primary teeth: 7-year study on three resin-modified glass ionomer cements and a compomer. European Journal of Oral Sciences. 2004;112(2):188-96. | Restorative dentistry |
| Qvist V, Laurberg L, Poulsen A, Teglers PT. Eight-year study on conventional glass ionomer and amalgam restorations in primary teeth. Acta Odontologica Scandinavica. 2004;62(1):37-45. | Restorative dentistry |
| Raborn GW, Martel AY, Grace MG, McGaw WT. Herpes labialis in skiers: randomized clinical trial of acyclovir cream versus placebo. Oral Surgery, Oral Medicine, Oral Pathology, Oral Radiology, and Endodontics. 1997;84:641-5. | Irrelevant |
| Ram D, Moskovitz M, Fuks AB. Ferric sulfate versus formocresol in pulpotomized primary molars: clinical and radiographic results (Divisional abstracts 2000). Journal of Dental Research. 2001;80(April 2001 Issue 4):1311 (Abs 20). | Abstract only |
| Ramakrishna Y. Dental considerations in the management of children suffering from sickle cell disease: a case report. Journal of the Indian Society of Pedodontics & Preventive Dentistry. 2007;25(3):140-3. | Case report |
| Ramirez-Perez AH, Sauvant D, Meschy F. Effect of phosphate solubility on phosphorus kinetics and ruminal fermentation activity in dairy goats. Animal Feed Science and Technology. 2009;149(3-4):209-27. | Irrelevant |
| Rashad A, el-Attar A. Dysrhythmias during oral surgery--effect of combined local and general anesthesia. Middle East Journal of Anesthesiology. 1990;10(5):499-505. | Anesthesia |
| Rasimick BJ, Wan J, Musikant BL, Deutsch AS. A review of failure modes in teeth restored with adhesively luted endodontic dowels [review]. Journal of Prosthodontics. 2010;19(8):639-46. | Review |
| Rasimick BJ, Wan J, Musikant BL, Deutsch AS. A Review of Failure Modes in Teeth Restored with Adhesively Luted Endodontic Dowels. Journal of Prosthodontics-Implant Esthetic and Reconstructive Dentistry. 2010;19(8):56-63. | Review |
| Raslan N, Wetzel WE. Exposed human pulp caused by trauma and/or caries in primary dentition: a histological evaluation. Dental Traumatology. 2006;22(3):145-53. | In vitro study |
| Ravindranathan N. Allergic reaction to lignocaine. A case report. British Dental Journal. 1975;138(3):101-2. | Anesthesia |
| Ravn JJ. Follow-up study of permanent incisors with complicated crown fractures after acute trauma. Scandinavian Journal of Dental Research. 1982;90(5):363-72. | Permanent incisors |
| Ravn JJ, Svarrer M. [A clinicoradiographic follow-up study of coronal vital pulpotomy in 200 primary molars treated with zinc-oxide-eugenol paste]. [Danish]. Tandlaegebladet. 1968;72(8):718-26. | No RCT |
| Raygot CG, Chai J, Jameson DL. Fracture resistance and primary failure mode of endodontically treated teeth restored with a carbon fiber-reinforced resin post system in vitro. International Journal of Prosthodontics. 2001;14(2):141-5. | In vitro study |
| Reali-Forster L. [Clinical results with a new product for root canal obturation]. [French]. Chirurgien-Dentiste de France. 1967;37(38):45-51. | Permanent teeth |
| Reddy S, Ramakrishna Y. Evaluation of antimicrobial efficacy of various root canal filling materials used in primary teeth: a microbiological study. Journal of Clinical Pediatric Dentistry. 2007;31(3):193-8. | In vitro study |
| Reddy VV, Fernandes. Clinical and radiological evaluation of zinc oxide-eugenol and Maisto's paste as obturating materials in infected primary teeth--nine months study. Journal of the Indian Society of Pedodontics & Preventive Dentistry. 1996;14(2):39-44. | Abstract only |
| Redig DF. A comparison and evaluation of two formocresol pulpotomy technics utilizing "Buckley's" formocresol. Journal of Dentistry for Children. 1968;35(1):22-30. | No RCT |
| Reichart P, Tantiniran D. Dens evaginatus in the Thai. An evaluation of fifty-one cases. Oral Surgery, Oral Medicine, Oral Pathology. 1975;39(4):615-21. | Dens invaginatus |
| Remmers T, Glickman G, Spears R, He J. The efficacy of IntraFlow intraosseous injection as a primary anesthesia technique. Journal of endodontics. 2008;34(3):280-3. | Anesthesia |
| Replogle K, Reader A, Nist R, Beck M, Weaver J, Meyers WJ. Anesthetic efficacy of the intraosseous injection of 2% lidocaine (1:100,000 epinephrine) and 3% mepivacaine in mandibular first molars. Oral Surgery, Oral Medicine, Oral Pathology, Oral Radiology, and Endodontics. 1997;83:30-7. | Anesthesia |
| Ribeiro DA, Bazo AP, Franchi CAD, Marques MEA, Salvadori DMF. Chlorhexidine induces DNA damage in rat peripheral leukocytes and oral mucosal cells. Journal of Periodontal Research. 2004;39(5):358-61. | Animal study |
| Riccioli GA. [Conservative therapy of the deciduous teeth]. [Italian]. Mondo Odontostomatologico. 1971;14(1):65-7. | Case report |
| Riccitiello F, Stabile P, Amato M, Rengo S, D'Ambrosio C. The treatment of the large periradicular endodontic injury. Minerva Stomatologica. 2011;60(9):417-26. | Stomatology |
| Ricieri CB, Sonoda CK, Aranega AM, Panzarini SR, Poi WR, Sundefeld M, et al. Healing process of incisor teeth of diabetic rats replanted after storage in milk. Dental Traumatology. 2009;25(3):284-9. | Incisors |
| Rickoff B, Trowbridge H, Baker J, Fuss Z, Bender IB. Effects of thermal vitality tests on human dental pulp. Journal of Endodontics. 1988;14(10):482-5. | Biomaterials not compared |
| Ricucci D, Siqueira JF, Bate AL, Ford TRP. Histologic Investigation of Root Canal-treated Teeth with Apical Periodontitis: A Retrospective Study from Twenty-four Patients. Journal of Endodontics. 2009;35(4):493-502. | Retrospective study |
| Rifai K, Chidiac JJ, Hawwa N, Baliki M, Jabbur SJ, Saade NE. Occlusion of dentinal tubules and selective block of pulp innervation prevent the nociceptive behaviour induced in rats by intradental application of irritants. Archives of Oral Biology. 2004;49(6):457-68. | Biological study |
| Ring KC, Murray PE, Namerow KN, Kuttler S, Garcia-Godoy F. The comparison of the effect of endodontic irrigation on cell adherence to root canal dentin. Journal of Endodontics. 2008;34(12):1474-9. | In vitro study |
| Ringel AM, Patterson SS, Newton CW, Miller CH, Mulhern JM. In vivo evaluation of chlorhexidine gluconate solution and sodium hypochlorite solution as root canal irrigants. Journal of Endodontics. 1982;8(5):200-4. | Permanent teeth |
| Riordan PJ. Secular changes in treatment in a school dental service. Community Dental Health. 1995;12(4):221-5. | Epidemiologic study |
| Ripa LW. [Pulp therapy in live deciduous teeth]. [Spanish]. Fauchard. 1971;2(6):114-7. | Review |
| Risso PA, Cunha AJ, Araujo MC, Luiz RR. Postobturation pain and associated factors in adolescent patients undergoing one- and two-visit root canal treatment. Journal of Dentistry. 2008;36(11):928-34. | Permanent teeth |
| Risso PA, da Cunha A, de Araujo MCP, Luiz RR. Postoperative pain and associated factors in adolescent patients undergoing two-visit root canal therapy. Australian Endodontic Journal. 2009;35(2):89-92. | Permanent teeth |
| Ritwik P, Cuisia ZV, Dabir P, Musselman RJ. MTA pulpotomies in the primary molars of children: Results after 3 years. (Abstract). International journal of paediatric dentistry / the British Paedodontic Society [and] the International Association of Dentistry for Children. 2003;13(Suppl 1):11. | Abstract only |
| Rivera N, Reyes E, Mazzaoui S, Moron A. Pulpal therapy for primary teeth: formocresol vs electrosurgery: a clinical study. Journal of Dentistry for Children (Chicago, Ill. 2003). 70(1):71-3. | No RCT |
| Roach RP, Hatton JF, Gillespie MJ. Prevention of the ingress of a known virulent bacterium into the root canal system by intracanal medications. Journal of Endodontics. 2001;27(11):657-60. | Permanent teeth |
| Roberts SC, Brilliant JD. Tricalcium phosphate as an adjunct to apical closure in pulpless permanent teeth. Journal of endodontics. 1975;1(8):263-9. | Permanent teeth |
| Robertson D, Smith AJ. The microbiology of the acute dental abscess. Journal of Medical Microbiology. 2009;58(2):155-62. | In vitro study |
| Rocas IN, Siqueira JE, Debelian GJ. Analysis of Symptomatic and Asymptomatic Primary Root Canal Infections in Adult Norwegian Patients. Journal of Endodontics. 2011;37(9):1206-12. | Adult |
| Rocas IN, Siqueira JF. Characterization of Dialister species in infected root canals. Journal of Endodontics. 2006;32(11):1057-61. | Bacteriology |
| Rocha CT, Rossi MA, Leonardo MR, Rocha LB, Nelson P, Silva LAB. Biofilm on the apical region of roots in primary teeth with vital and necrotic pulps with or without radiographically evident apical pathosis. International Endodontic Journal. 2008;41(8):664-9. | In vitro study |
| Rocha M, Baroni R, Santos L, Girardi K. Ca(OH)2 and MTA pulpotomies in primary teeth: One year results [abstract]. International Journal of Paediatric Dentistry. 1999;9(Suppl 1):102 (Abst P5.32). | Abstract only |
| Rodd HD, Davidson LE, Livesey S, Cooke ME. Survival of intentionally retained permanent incisor roots following crown root fractures in children. Dental Traumatology. 2002;18(2):92-7. | Permanent incisor |
| Rodriguez-Lozano FJ, Bueno C, Insausti CL, Meseguer L, Ramirez MC, Blanquer M, et al. Mesenchymal stem cells derived from dental tissues. International Endodontic Journal. 2011;44(9):800-6. | Biological study |
| Roeleveld AC, van Amerongen WE, Mandari GJ. Influence of residual caries and cervical gaps on the survival rate of Class II glass ionomer restorations. European archives of paediatric dentistry : official journal of the European Academy of Paediatric Dentistry. 2006;7(2):85-91. | Restorative dentistry |
| Rosenberg PA, Babick PJ, Schertzer L, Leung A. The effect of occlusal reduction on pain after endodontic instrumentation. Journal of Endodontics. 1998;24(7):492-6. | Permanent teeth |
| Rossi-Fedele G, De Figueiredo JAP. Use of a bottle warmer to increase 4% sodium hypochlorite tissue dissolution ability on bovine pulp. Australian Endodontic Journal. 2008;34(1):39-42. | Animal study |
| Rost A. [Further information on the subject of ocalexic therapy. Histopathologic studies on periapical healing following conservative treatment of teeth affected with extensive chronic periapical lesions]. [Italian]. Dental Cadmos. 1966;34(5):637-54. | In vitro study |
| Rothman MS. Formocresol pulpotomy: a practical procedure for permanent teeth. General Dentistry. 1977;25(5):39-41. | Permanent teeth |
| Ru YJ, Bao YM. Feeding dry sows ad libitum with high fibre diets. Asian-Australasian Journal of Animal Sciences. 2004;17(2):283-300. | Irrelevant |
| Ruiz-Esparza CL, Garrocho-Rangel A, Gonzalez-Amaro AM, Flores-Reyes H, Pozos-Guillen AJ. Reduction in bacterial loading using 2% chlorhexidine gluconate as an irrigant in pulpectomized primary teeth: a preliminary report. Journal of Clinical Pediatric Dentistry. 2011;35(3):265-70. | Biomaterials not compared |
| Rule DC, Winter GB. Root growth and apical repair subsequent to pulpal necrosis in children. British Dental Journal. 1966;120(12):586-90. | Case report |
| Rush DE, Abdel-Haq N, Zhu JF, Aamar B, Malian M. Clindamycin versus Unasyn in the treatment of facial cellulitis of odontogenic origin in children. Clinical Pediatrics. 2007;46(2):154-9. | Cellulitis |
| Russo Mde C, Okamoto T, Holland R. Treatment of inflamed pulp in deciduous teeth. Histological study in dog. Bulletin of Tokyo Dental College. 1972;13(1):9-20. | In vitro study |
| Rutledge RE, Montgomery S. Effect of intracanal medicaments on the sealing ability of TERM. Journal of Endodontics. 1990;16(6):260-4. | Permanent teeth |
| Sacramento PA, De Carvalho FG, Pascon FM, Borges AF, Alves MC, Hosoya Y, et al. Influence of NaOCl irrigation and water storage on the degradation and microstructure of the resin/primary dentin interface. Journal of Adhesive Dentistry. 2011;13(3):213-20. | Restorative dentistry |
| Sadeghi S, Dibaei M. Prevalence of odontogenic sinus tracts in 728 endodontically treated teeth. Medicina Oral Patologia Oral Y Cirugia Bucal. 2011;16(2):E296-E9. | Biomaterials not compared |
| Sagiassif O, Douer D, Shaked N, Russell SW, Witz IP. MYELOPROLIFERATION IN LONG-TERM PLASMACYTOMA-REGRESSOR MICE. International Journal of Cancer. 1994;56(2):208-13. | Irrelevant |
| Saito D, Coutinho LL, Saito CPB, Tsai SM, Hoefling JF, Goncalves RB. Real-time Polymerase Chain Reaction Quantification of Porphyromonas gingivalis and Tannerella forsythia in Primary Endodontic Infections. Journal of Endodontics. 2009;35(11):1518-24. | In vitro study |
| Sakai VT, Moretti ABS, Oliveira TM, Fornetti APC, Santos CF, Machado M, et al. Summary of: Pulpotomy of human primary molars with MTA and Portland cement: a randomised controlled trial. British Dental Journal. 2009;207(3):128-9. | Editor’s summary and comments |
| Salama FS. Influence of zinc-oxide eugenol, formocresol, and ferric sulfate on bond strength of dentin adhesives to primary teeth. Journal of Contemporary Dental Practice [Electronic Resource]. 2005;6(3):14-21. | In vitro study |
| Salgado RJ, Moura-Netto C, Yamazaki AK, Cardoso LN, de Moura AA, Prokopowitsch I. Comparison of different irrigants on calcium hydroxide medication removal: microscopic cleanliness evaluation. Oral Surgery Oral Medicine Oral Pathology Oral Radiology & Endodontics. 2009;107(4):580-4. | Permanent teeth |
| Salim S, Santini A, Husham A. An in-vitro study of microleakage around class V cavities bonded with a self-etching material versus a conventional two-bottle system. Primary dental care : journal of the Faculty of General Dental Practitioners (UK). 2006;13(3):107-11. | In vitro study |
| Salim S, Santini A, Safar KN. Microleakage around glass-ceramic insert restorations luted with a high-viscous or flowable composite. Journal of esthetic and restorative dentistry : official publication of the American Academy of Esthetic Dentistry. 2005;.. [et al.]. 17(1):30-8; discussion 9. | In vitro study |
| Saloum FS, Baumgartner JC, Marshall G, Tinkle J. A clinical comparison of pain perception to the Wand and a traditional syringe. Oral Surgery, Oral Medicine, Oral Pathology, Oral Radiology, and Endodontics. 2000;89(6):691-5. | Anesthesia |
| Sampaio FC, Freitas C, Cabral MBD, Machado A. Dental caries and treatment needs among indigenous people of the Potiguara Indian reservation in Brazil. Revista Panamericana De Salud Publica-Pan American Journal of Public Health. 2010;27(4):246-51. | Epidemiologic study |
| Sandalli N, Cildir S, Guler N. Clinical investigation of traumatic injuries in Yeditepe University, Turkey during the last 3 years. Dental Traumatology. 2005;21(4):188-94. | Traumatology |
| Sandler ES, Frankl SN, Ruben MP. Te histological response of the dental pulp to cresatin. Journal of Dentistry for Children. 1971;38(1):49-58. | In vitro study |
| Sandor GK, Nish IA, Carmichael RP. Comparison of conventional surgery with motorized trephine in bone harvest from the anterior iliac crest. Oral Surgery, Oral Medicine, Oral Pathology, Oral Radiology, and Endodontics. 2003;95(2):150-5. | Irrelevant |
| Santini A. Assessment of the pulpotomy technique in human first permanent mandibular molars. Use of two direct inspection criteria. British Dental Journal. 1983;155(5):151-4. | Permanent teeth |
| Santini A. Long-term clinical assessment of pulpotomies with calcium hydroxide containing ledermix in human permanent premolars and molars. Acta odontol pediatr. 1986;7:45-50. | Permanent teeth |
| Santini A, Ivanovic V, Tan CL, Ibbetson R. Effect of prolonged thermal cycling on microleakage around Class V cavities restored with glass-ceramic inserts with different coefficients of thermal expansion: an in vitro study. Primary dental care : journal of the Faculty of General Dental Practitioners (UK). 2006;13(4):147-53. | In vitro study |
| Santini A, Milia E. Microleakage around a low-shrinkage composite cured with a high-performance light. American journal of dentistry. 2004;17(2):118-22. | In vitro study |
| Santini AH. Intraoral comparison of calcium hydroxide (Calnex) alone and in combination with Ledermix in first permanent mandibular molars using two direct inspection criteria. Journal of Dentistry. 1985;13(1):52-9. | Permanent teeth |
| Sargenti A. [Rational root therapy of deciduous teeth with N2]. [German]. Zahnarztliche Praxis. 1975;26(9):198-200. | Review |
| Sari S, Duruturk L. Radiographic evaluation of periapical healing of permanent teeth with periapical lesions after extrusion of AH Plus sealer. Oral Surgery Oral Medicine Oral Pathology Oral Radiology and Endodontology. 2007;104(3):E54-E9. | Permanent teeth |
| Sari S, Ozalp N, Ozer L. The effect of formocresol on bond strength of adhesive materials to primary dentine. Journal of Oral Rehabilitation. 2004;31(7):671-4. | In vitro study |
| Sari S, Sonmez D. Internal resorption treated with mineral trioxide aggregate in a primary molar tooth: 18-month follow-up. Journal of Endodontics. 2006;32(1):69-71. | Case report |
| Sassone LM, Fidel RA, Faveri M, Guerra R, Figueiredo L, Fidel SR, et al. A microbiological profile of symptomatic teeth with primary endodontic infections. Journal of Endodontics. 2008;34(5):541-5. | In vitro study |
| Sathorn C, Parashos P, Messer H. Antibacterial efficacy of calcium hydroxide intracanal dressing: a systematic review and meta-analysis. [Review] [45 refs]. International Endodontic Journal. 2007;40(1):2-10. | Review |
| Sathorn C, Parashos P, Messer H. The prevalence of postoperative pain and flare-up in single- and multiple-visit endodontic treatment: a systematic review. International Endodontic Journal. 2008;41(2):91-9. | Review |
| Sathorn C, Parashos P, Messer H. Australian endodontists' perceptions of single and multiple visit root canal treatment. International Endodontic Journal. 2009;42(9):811-8. | Permanent teeth |
| Sathorn C, Parashos P, Messer HH. Effectiveness of single- versus multiple-visit endodontic treatment of teeth with apical periodontitis: a systematic review and meta-analysis. [Review] [55 refs]. International Endodontic Journal. 2005;38(6):347-55. | Review |
| Sawicki L, Pameijer CH, Emerich K, Adamowicz-Klepalska B. Histological evaluation of mineral trioxide aggregate and calcium hydroxide in direct pulp capping of human immature permanent teeth. American Journal of Dentistry. 2008;21(4):262-6. | Permanent teeth |
| Say R, Birlik E, Erdemgil Z, Denizli A, Ersoz A. Removal of mercury species with dithiocarbamate-anchored polymer/organosmectite composites. Journal of Hazardous Materials. 2008;150(3):560-4. | Irrelevant |
| Sayegh FS, Reed AJ. Correlated clinical and histological evaluation of Hydrex in pulp therapy. Journal of Dentistry-Child. 1967;34(6, Nov):471-7. | Intact human teeth, no RCT |
| Scelza MZ, Linhares AB, da Silva LE, Granjeiro JM, Alves GG. A multiparametric assay to compare the cytotoxicity of endodontic sealers with primary human osteoblasts. International Endodontic Journal. 2012;45(1):12-8. | In vitro study |
| Schafer E, Bossmann K. Antimicrobial effect of camphorated chloroxylenol (ED 84) in the treatment of infected root canals. Journal of Endodontics. 1999;25(8):547-51. | Permanent teeth |
| Schafer E, Bossmann K. Antimicrobial efficacy of chloroxylenol and chlorhexidine in the treatment of infected root canals. American Journal of Dentistry. 2001;14(4):233-7. | Permanent teeth |
| Schmitzer V, Slatnar A, Mikulic-Petkovsek M, Veberic R, Krska B, Stampar F. Comparative study of primary and secondary metabolites in apricot (Prunus armeniaca L.) cultivars. Journal of the Science of Food and Agriculture. 2011;91(5):860-6. | Irrelevant |
| Schmoldt SJ, Kirkpatrick TC, Rutledge RE, Yaccino JM. Reinforcement of Simulated Immature Roots Restored with Composite Resin, Mineral Trioxide Aggregate, Gutta-percha, or a Fiber Post after Thermocycling. Journal of Endodontics. 2011;37(10):1390-3. | Permanent teeth |
| Schneider DW. Triamcinolone acetonide-demethylchlortetracycline HCl treatment in endodontic practice. Journal of Oral Medicine. 1968;23(2):51-5. | Biomaterials not compared |
| Schroeder A, Asal E. [Addition of corticoids with calcium hydroxide and dentin formation]. [German]. Deutsche Zahnarztliche Zeitschrift. 1971;26(4):449-55. | Biomaterials not compared |
| Schwab O, Maness T, Bull G, Roberts D. Modeling the effect of changing market conditions on mountain pine beetle salvage harvesting and structural changes in the British Columbia forest products industry. Canadian Journal of Forest Research-Revue Canadienne De Recherche Forestiere. 2009;39(10):1806-20. | Irrelevant |
| Schwartz-Arad D, Levin L. Post-traumatic use of dental implants to rehabilitate anterior maxillary teeth. Dental Traumatology. 2004;20(6):344-7. | Implant |
| Seale NS, Glickman GN. Contemporary perspectives on vital pulp therapy: views from the endodontists and pediatric dentists. Pediatric Dentistry. 2008;30(3):261-7. | Review |
| Seemann R, Passek G, Zimmer S, Roulet JF. The effect of an oral hygiene program on oral levels of volatile sulfur compounds (VSC). Journal of Clinical Dentistry. 2001;12(4):104-7. | Biomaterials not compared |
| Segura JJ, Baldizon-Rodriguez C, Toledo-Balladares S, Flores-TreviÒo JJ, Calzado-Flores C. A new therapeutic scheme of ibuprofen to treat postoperatory endodontic dental pain. Proceedings of the Western Pharmacology Society. 2000;43:89-91. | Oral medication |
| Sevimay S, Oztan MD, Dalat D. Effects of calcium hydroxide paste medication on coronal leakage. Journal of Oral Rehabilitation. 2004;31(3):240-4. | In vitro study |
| Shafik A. Magnetic stimulation: a novel method for the treatment of chronic constipation. Minimally Invasive Therapy & Allied Technologies. 1998;7(5):477-81. | Irrelevant |
| Shah N, Logani A, Bhaskar U, Aggarwal V. Efficacy of revascularization to induce apexification/apexogensis in infected, nonvital, immature teeth: a pilot clinical study.[Erratum appears in J Endod. 2008 Oct;34(10):1263]. Journal of Endodontics. 2008;34(8):919-25; Discussion 1157. | Permanent teeth |
| Shahani MN, Subba Reddy VV. Comparison of antimicrobial substantivity of root canal irrigants in instrumented root canals up to 72 h: an in vitro study. Journal of the Indian Society of Pedodontics & Preventive Dentistry. 2011;29(1):28-33. | In vitro study |
| Sharaf AA. Evaluation of mandibular infiltration versus block anesthesia in pediatric dentistry. ASDC J Dent Child. 1997;64(4):276-81. | Anesthesia |
| Sharma DS, Barjatya K, Agrawal A. Intra-coronal bleaching in young permanent and primary tooth with biologic perspectives. Journal of Clinical Pediatric Dentistry. 2011;35(4):349-52. | Bleaching |
| Shelley PQ, Johnson BR, BeGole EA. Use of an electronic patient record syatem to evaluate restorative treatment followingroot canal therapy. Journal of Dental Education. 2007;71(10):1333-9. | Restorative dentistry |
| Shen YY, Chen K, Xu N. [Osteogenic capacity of human deciduous dental pulp stem cells in vitro]. [Chinese]. Nan Fang Yi Ke Da Xue Xue Bao = Journal of Southern Medical University. 2010;30(1):96-9. | In vitro study |
| Shiflett K, White SN. Microleakage of cements for stainless steel crowns. Pediatric dentistry. 1997;19(4):262-6. | In vitro study |
| Shiki A, Ogata K. [Evaluation of day-care propofol anesthesia with low-dose sevoflurane for dental treatment of the mentally handicapped]. Journal of Japanese Dental Society of Anesthesiology. 2005;33(2):180-5. | Anesthesia |
| Shin SY, Albert JS, Mortman RE. One step pulp revascularization treatment of an immature permanent tooth with chronic apical abscess: a case report. International Endodontic Journal. 2009;42(12):1118-26. | Permanent teeth |
| Shinkai K, Taira Y, Suzuki M, Kato C, Yamauchi J, Suzuki S, et al. Dentin bond strength of an experimental adhesive system containing calcium chloride, synthetic peptides derived from dentin matrix protein 1 (pA and pB), and hydroxyapatite for direct pulp capping and as a bonding agent. Odontology/The Society of the Nippon Dental University. 2010;98(2):110-6. | In vitro study |
| Silva GA, Lanza LD, Lopes-Junior N, Moreira A, Alves JB. Direct pulp capping with a dentin bonding system in human teeth: a clinical and histological evaluation. Operative Dentistry. 2006;31(3):297-307. | In vitro study |
| Silva LA, Leonardo MR, Nelson-Filho P, Tanomaru JM. Comparison of rotary and manual instrumentation techniques on cleaning capacity and instrumentation time in deciduous molars. Journal of Dentistry for Children (Chicago, Ill. 2004). 71(1):45-7. | In vitro study |
| Simancas-Pallares MA, Diaz-Caballero AJ, Luna-Ricardo LM. Mineral trioxide aggregate in primary teeth pulpotomy. A systematic literature review. Medicina Oral Patologia Oral Y Cirugia Bucal. 2010;15(6):E942-E6. | Review |
| Simon S, Rilliard F, Berdal A, Machtou P. The use of mineral trioxide aggregate in one-visit apexification treatment: a prospective study. International Endodontic Journal. 2007;40(3):186-97. | Permanent teeth |
| Simsek S, Duruturk L. A flow cytometric analysis of the biodefensive response of deciduous tooth pulp to carious stimuli during physiological root resorption. Archives of Oral Biology. 2005;50(5):461-8. | In vitro study |
| Siqueira JF, Jr., Guimaraes-Pinto T, Rocas IN. Effects of chemomechanical preparation with 2.5% sodium hypochlorite and intracanal medication with calcium hydroxide on cultivable bacteria in infected root canals. Journal of Endodontics. 2007;33(7):800-5. | Permanent teeth |
| Siqueira JF, Jr., Paiva SS, Rocas IN. Reduction in the cultivable bacterial populations in infected root canals by a chlorhexidine-based antimicrobial protocol. Journal of Endodontics. 2007;33(5):541-7. | Permanent teeth |
| Siqueira JF, Rocas IN. Dialister pneumosintes can be a suspected endodontic pathogen. Oral Surgery Oral Medicine Oral Pathology Oral Radiology and Endodontics. 2002;94(4):494-8. | Bacteriology |
| Siqueira JF, Rocas IN. Molecular detection and identification of Synergistes phylotypes in primary endodontic infections. Oral Diseases. 2007;13(4):398-401. | Bacteriology |
| Siqueira JF, Rocas IN. Optimising single-visit disinfection with supplementary approaches: A quest for predictability. Australian Endodontic Journal. 2011;37(3):92-8. | Permanent teeth |
| Siqueira JF, Jr., Rocas IN, Paiva SS, Guimaraes-Pinto T, Magalhaes KM, Lima KC. Bacteriologic investigation of the effects of sodium hypochlorite and chlorhexidine during the endodontic treatment of teeth with apical periodontitis. Oral Surgery Oral Medicine Oral Pathology Oral Radiology & Endodontics. 2007;104(1):122-30. | Permanent teeth |
| Smadi L. Comparison between two methods of working length determination and its effect on radiographic extent of root canal filling: A clinical study [ISRCTN71486641]. BMC Oral Health. 2006;6(pp 6P):4. | Permanent teeth |
| Smadi LM, Khraisat AS, Al-Tarawneh SK. Choice of intracanal medication and obturation techniques amongst Jordanian dentists. Jordan Medical Journal. 2007;41(1):28-36. | Public health |
| Smith M, Lennon MA, Robinson PG. Students' clinical experience on outreach placements. European Journal of Dental Education. 2010;14(1):7-11. | Public health |
| Soares F, Varella CH, Pileggi R, Adewumi A, Guelmann M. Impact of Er,Cr:YSGG laser therapy on the cleanliness of the root canal walls of primary teeth. Journal of Endodontics. 2008;34(4):474-7. | In vitro study |
| Sogbe de Agell R. [Clinical and radiographic evaluation of deciduous molars with necrotic pulp treated with two concentrations of formocresol]. [Spanish]. Acta Odontologica Venezolana. 1989;27(1):3-9. | No RCT |
| Song JS, Stefanik D, Damek-Poprawa M, Alawi F, Akintoye SO. Differentiation and regenerative capacities of human odontoma-derived mesenchymal cells. Differentiation. 2009;77(1):29-37. | In vitro study |
| Sonmez D, Duruturk L. Ca(OH)(2) pulpotomy in primary teeth. Part I: internal resorption as a complication following pulpotomy. Oral Surgery Oral Medicine Oral Pathology Oral Radiology and Endodontology. 2008;106(2):E94-E8. | Biomaterials not compared |
| Sonmez D, Duruturk L. Summary of: Success rate of calcium hydroxide pulpotomy in primary molars restored with amalgam and stainless steel crowns. British Dental Journal. 2010;208(9):408-9. | Editor’s comment |
| Sonmez D, Duruturk L. Success rate of calcium hydroxide pulpotomy in primary molars restored with amalgam and stainless steel crowns. British Dental Journal. 2010;208(9):E18; discussion 408-9. | Resrorative dentistry |
| Sortino F, Lombardo C, Sciacca A. Silk and polyglycolic acid in oral surgery: a comparative study. Oral Surgery, Oral Medicine, Oral Pathology, Oral Radiology, and Endodontics. 2008;105(3):e15-8. | Surgery |
| Spedding RH. Pulp therapy for primary teeth. Survey of the North American Dental Schools. Journal of Dentistry for Children. 1968;35(5):360-7. | Survey |
| Srinivasan V, Patchett CL, Waterhouse PJ. Is there life after Buckley's Formocresol? Part I -- a narrative review of alternative interventions and materials. [Review] [118 refs]. International Journal of Paediatric Dentistry. 2006;16(2):117-27. | Review |
| Starkey DL, Anderson RW, Pashley DH. An evaluation of the effect of methylene blue dye pH on apical leakage. Journal of Endodontics. 1993;19(9):435-9. | In vitro study |
| Stoll R, Remes H, Kunzelmann KH, Stachniss V. Marginal characteristics of different filling materials and filling methods with standardized cavity preparation. The journal of adhesive dentistry. 2000;2(2):129-38. | Permanent teeth |
| Straffon LH, Corpron RL, Bruner FW, Daprai F. Twenty-four-month clinical trial of visible-light-activated cavity liner in young permanent teeth. Journal of Dentistry for Children. 1991;58(2):124-8. | Permanent teeth |
| Sunnegardh-Gronberg K, van Dijken JW, Lindberg A, Horstedt P. Interfacial adaptation of a calcium aluminate cement used in class II cavities, in vivo. Clinical oral investigations. 2004;8(2):75-80. | Restorative dentistry |
| Svec P, Sedlacek I, Zackova L, Novakova D, Kukletova M. Lactobacillus spp. Associated with Early Childhood Caries. Folia Microbiologica. 2009;54(1):53-8. | Bacteriology |
| Svensson CI, Medicherla S, Malkmus S, Jiang Y, Ma JY, Kerr I, et al. Role of p38 mitogen activated protein kinase in a model of osteosarcoma-induced pain. Pharmacology Biochemistry and Behavior. 2008;90(4):664-75. | Irrelevant |
| Swanson KS, Dowd SE, Suchodolski JS, Middelbos IS, Vester BM, Barry KA, et al. Phylogenetic and gene-centric metagenomics of the canine intestinal microbiome reveals similarities with humans and mice. Isme Journal. 2011;5(4):639-49. | Irrelevant |
| Swanson TK, Feigal RJ, Tantbirojn D, Hodges JS. Effect of adhesive systems and bevel on enamel margin integrity in primary and permanent teeth. Pediatric dentistry. 2008;30(2):134-40. | Restorative dentistry |
| Szabo H. [Experience with the treatment of deep dental caries and pulpitis in deciduous molars with N2]. [German]. Zahnarztliche Praxis. 1969;20(19):217-8. | Title only |
| Szabo H, Beczner S, Matavovszky M. [Dental pulp treatment with "N2" material in deciduous molars]. [Hungarian]. Fogorvosi Szemle. 1968;61(12):268-70. | Translation problem |
| Szpringer M, Janicha J, Augustyniak L. [Ledermix in the treatment of pulp inflammation in children]. [Polish]. Czasopismo Stomatologiczne. 1977;30(6):445-53. | Translation problem |
| Tag-Eldin MA, Gadallah MA, Ai-Tayeb MN, Abdel-Aty M, Mansour E, Sallem M. Prevalence of female genital cutting among Egyptian girls. Bulletin of the World Health Organization. 2008;86(4):269-74. | Irrelevant |
| Takushige T, Cruz EV, Asgor Moral A, Hoshino E. Endodontic treatment of primary teeth using a combination of antibacterial drugs. International Endodontic Journal. 2004;37(2):132-8. | Biomaterials not compared |
| Tanaka M, Kamiya Y, Suzuki T, Nakai Y. Effect of citrus pulp silage feeding on concentration of beta-cryptoxanthin in plasma and milk of dairy cows. Animal Science Journal. 2010;81(5):569-73. | Irrelevant |
| Tang G, Samaranayake LP, Yip HK. Molecular evaluation of residual endodontic microorganisms after instrumentation, irrigation and medication with either calcium hydroxide or Septomixine. Oral Diseases. 2004;10(6):389-97. | Permanent teeth |
| Tang W, Song B, Zhou ZS, Lu GS. Intrathecal administration of resiniferatoxin produces analgesia against prostatodynia in rats. Chinese Medical Journal. 2007;120(18):1616-21. | Animal study |
| Tannure PN, Azevedo CP, Barcelos R, Gleiser R, Primo LG. Long-term outcomes of primary tooth pulpectomy with and without smear layer removal: a randomized split-mouth clinical trial. Pediatric Dentistry. 2011;33(4):316-20. | Anterior teeth |
| Tannure PN, Barcelos R, Portela MB, Gleiser R, Primo LG. Histopathologic and SEM analysis of primary teeth with pulpectomy failure. Oral Surgery Oral Medicine Oral Pathology Oral Radiology and Endodontology. 2009;108(1):E29-E33. | In vitro study |
| Tanomaru-Filho M, Spinola SG, Reis JM, Chavez-Andrade GM, Guerreiro-Tanomaru JM. In vitro sealing ability of temporary restorative materials used in endodontics. General Dentistry. 2009;57(6):622-5. | In vitro study |
| Tchaou WS, Turng BF, Minah GE, Coll JA. Inhibition of pure cultures of oral bacteria by root canal filling materials. Pediatric Dentistry. 1996;18(7):444-9. | In vitro study |
| Teixeira LS, Demarco FF, Coppola MC, Bonow ML. Clinical and radiographic evaluation of pulpotomies performed under intrapulpal injection of anaesthetic solution. International Endodontic Journal. 2001;34(6):440-6. | Permanent teeth |
| Tervit C, Paquette L, Torneck CD, Basrani B, Friedman S. Proportion of healed teeth with apical periodontitis medicated with two percent chlorhexidine gluconate liquid: a case-series study. Journal of endodontics. 2009;35(9):1182-5. | Case-series |
| Thomson A, Kahler B. Regenerative endodontics--biologically-based treatment for immature permanent teeth: a case report and review of the literature. [Review]. Australian Dental Journal. 2010;55(4):446-52. | Permanent teeth |
| Thylstrup A, Boyar RM, Holmen L, Bowden GH. A light and scanning electron microscopic study of enamel decalcification in children living in a water-fluoridated area. Journal of Dental Research. 1990;69(10):1626-33. | In vitro study |
| Tianviwat S, Chongsuvivatwong V, Birch S. Estimating unit costs for dental service delivery in institutional and community-based settings in southern Thailand. Asia-Pacific Journal of Public Health. 2009;21(1):84-93. | Cost |
| Togay B, Atac A, Cehreli ZC. Microleakage and micromorphology of the resin-dentin interface in primary molars following different endodontic irrigation regimens. Journal of Clinical Pediatric Dentistry. 2006;31(2):98-103. | In vitro study |
| Topaloglu-Ak A, Eden E, Frencken JE. Association between intra-radicular posts and periapical lesions in endodontically treated teeth. Journal of Applied Oral Science. 2007;15(3):235-40. | Permanent teeth |
| Torppa H. [A control study on vital amputations of mandibular first molars in school dentistry]. [Finnish]. Suomen Hammaslaakariseuran Toimituksia. 1971;67(3):202-8. | Surgery |
| Torstenson B. Pulpal reaction to a dental adhesive in deep human cavities. Endodontics and Dental Traumatology. 1995;11:172-6. | Permanent teeth |
| Townsend JA, Ganzberg S, Thikkurissy S. The effect of local anesthetic on quality of recovery characteristics following dental rehabilitation under general anesthesia in children. Anesthesia Progress. 2009;56(4):115-22. | Anesthesia |
| Trairatvorakul C, Piwat S. Comparative clinical evaluation of slot versus dovetail Class III composite restorations in primary anterior teeth. The Journal of clinical pediatric dentistry. 2004;28(2):125-9. | Restorative dentistry |
| Trankmann J, Claus U, Deutscher D. [Physiological and pathological root resorption in devitalized molars of the 1st dentition after root canal therapy]. [German]. Deutsche Zahnarztliche Zeitschrift. 1974;29(11):986-90. | No RCT |
| Trasatti C, Spears R, Gutmann JL, Opperman LA. Increased Tgf-beta 1 production by rat osteoblasts in the presence of PepGen P-15 in vitro. Journal of Endodontics. 2004;30(4):213-7. | Animal study |
| Trask PA. Formocresol pulpotomy on (young) permanent teeth. Journal of the American Dental Association. 1972;85(6):1316-23. | Permanent teeth |
| Trevisan CL, Panzarini SR, Brandini DA, Poi WR, Luvizuto ER, Dos Santos CLV, et al. Calcium Hydroxide Mixed With Camphoric p-Monochlorophenol or Chlorhexidine in Delayed Tooth Replantation. Journal of Craniofacial Surgery. 2011;22(6):2097-101. | Surgery |
| Trope M, Friedman S. PERIODONTAL HEALING OF REPLANTED DOG TEETH STORED IN VIASPAN, MILK AND HANK BALANCED SALT SOLUTION. Endodontics & Dental Traumatology. 1992;8(5):183-8. | Surgery |
| Trope M, Hupp JG, Mesaros SV. The role of the socket in the periodontal healing of replanted dogs' teeth stored in ViaSpan for extended periods. Endodontics & Dental Traumatology. 1997;13(4):171-5. | Surgery |
| Tsai TP, Su HL, Tseng LH. Glutaraldehyde preparations and pulpotomy in primary molars. Oral Surgery, Oral Medicine, Oral Pathology. 1993;76(3):346-50. | No RCT |
| Tselnik M, Baumgartner JC, Marshall JG. Bacterial leakage with mineral trioxide aggregate or a resin-modified glass ionomer used as a coronal barrier. Journal of Endodontics. 2004;30(11):782-4. | In vitro study |
| Tuna EB, Dincol ME, Gencay K, Aktoren O. Fracture resistance of immature teeth filled with BioAggregate, mineral trioxide aggregate and calcium hydroxide. Dental Traumatology. 2011;27(3):174-8. | Permanent teeth |
| Tunc ES, Saroglu I, Sari S, Gunhan O. The effect of sodium hypochlorite application on the success of calcium hydroxide pulpotomy in primary teeth. Oral Surgery Oral Medicine Oral Pathology Oral Radiology & Endodontics. 2006;102(2):e22-6. | Biomaterials not compared |
| Turner CL, Eggleston GW, Lunos S, Johnson N, Wiedmann TS, Bowles WR. Sniffing out endodontic pain: use of an intranasal analgesic in a randomized clinical trial. Journal of Endodontics. 2011;37(4):439-44. | Biomaterials not compared |
| Twetman S, Stecksen-Blicks C. Probiotics and oral health effects in children. [Review] [47 refs]. International Journal of Paediatric Dentistry. 2008;18(1):3-10. | Review |
| Tziafas D. The future role of a molecular approach to pulp-dentinal regeneration. Caries Research. 2004;38(3):314-20. | Biological study |
| Tziafas D, Koliniotou-Koumpia E, Tziafa C, Papadimitriou S. Effects of a new antibacterial adhesive on the repair capacity of the pulp-dentine complex in infected teeth. International Endodontic Journal. 2007;40(1):58-66. | Permanent teeth |
| Uctasli MB, Tinaz AC. Microleakage of different types of temporary restorative materials used in endodontics. Journal of Oral Science. 2000;42(2):63-7. | In vitro study |
| Ulmansky M, Sela J, Langer M, Yaari A. Response of pulpotomy wounds in normal human teeth to successively applied Ledermix and Calxyl. Arch Oral Biol. 1971;16(12):1393-8. | Premolars |
| Uloth VC, Dorris GM, Thring RW, Hogikyan RM, Wearing JT. In-situ production of polysulphide liquor in a kraft mill's causticizers .2. Process scale-up and economics. Pulp & Paper-Canada. 1996;97(5):43-6. | Irrelevant |
| Uzun O, Topuz O, Tinaz C, Nekoofar MH, Dummer PM. Accuracy of two root canal length measurement devices integrated into rotary endodontic motors when removing gutta-percha from root-filled teeth. International Endodontic Journal. 2008;41(9):725-32. | Permanent teeth |
| Vamnes JS, Lygre GB, Gronningsaeter AG, Gjerdet NR. Four years of clinical experience with an adverse reaction unit for dental biomaterials. Community Dentistry and Oral Epidemiology. 2004;32(2):150-7. | Epidemiologic study |
| van Amerongen WE, Mulder GR, Vingerling PA. Consequences of endodontic treatment in primary teeth. Part I: a clinical and radiographic study of the influence of formocresol pulpotomy on the life-span of primary molars. ASDC J Dent Child. 1986;53(5):364-70. | Biomaterials not compared |
| van Dijken JW, Pallesen U. Clinical performance of a hybrid resin composite with and without an intermediate layer of flowable resin composite: a 7-year evaluation. Dental Materials. 2011;27(2):150-6. | Restorative dentistry |
| van Gemert-Schriks MCM, van Amerongen WE, ten Cate JM, Aartman IHA. The effect of different dental treatment strategies on the oral health of children: a longitudinal randomised controlled trial. Clinical Oral Investigations. 2008;12(4):361-8. | No pulp treatment |
| Velkova A. [Treatment of pulpitis in deciduous molars]. [Czech]. Ceskoslovenska Stomatologie. 1977;77(4):277-81. | No RCT |
| Vergnes JN, Arrive E, Gourdy P, Hanaire H, Rigalleau V, Gin H, et al. Periodontal treatment to improve glycaemic control in diabetic patients: study protocol of the randomized, controlled DIAPERIO trial. Trials. 2009;10:Article no: 65. | Periodontology |
| Vernieks AA, Messer LB. Calcium hydroxide induced healing of periapical lesions: a study of 78 non-vital teeth. Journal of the British Endodontic Society. 1978;11(2):61-9. | Permanent teeth |
| Viada S, Rivera N, Nava S, Hernandez N, Moron A, Contreras J. Temporary and permanent restorations for fractured permanent teeth with immature apices: a clinical study. ASDC Journal of Dentistry for Children. 1997;64(6):414-6. | Permanent teeth |
| Vianna ME, Conrads G, Gomes B, Horz HP. Identification and quantification of Archaea involved in primary endodontic infections. Journal of Clinical Microbiology. 2006;44(4):1274-82. | Bacteriology |
| Vianna ME, Conrads G, Gomes B, Horz HP. Quantification and characterization of Synergistes in endodontic infections. Oral Microbiology and Immunology. 2007;22(4):260-5. | Bacteriology |
| Vianna ME, Horz HP, Gomes BP, Conrads G. In vivo evaluation of microbial reduction after chemo-mechanical preparation of human root canals containing necrotic pulp tissue. International endodontic journal. 2006;39(6):484-92. | Permanent teeth |
| Villa PA, Oberti G, Moncada CA, Vasseur O, Jaramillo A, TobÛn D, et al. Pulp-dentine complex changes and root resorption during intrusive orthodontic tooth movement in patients prescribed nabumetone. Journal of endodontics. 2005;31(1):61-6. | Orthodontics |
| Vinnichenko AV, Shkurina VA, Ruskova OI. [Treatment of chronic periodontitis of the deciduous and permanent teeth in children using Genis' silver paste]. [Russian]. Stomatologiia. 1981;60(3):67-9. | Periodontology |
| von Fraunhofer JA, Kurtzman GM, Norby CE. Resin-based sealing of root canals in endodontic therapy. General Dentistry. 2006;54(4):243-6. | Permanent teeth |
| Walker JD, Pinkham JR, Jakobsen J. Comparison of undergraduate pediatric dentistry clinical procedures from 1982-83 through 1996-97. Journal of Dentistry for Children. 1999;66(6):411-4. | Epidemiologic study |
| Walter K, Paulsson M, Wackerberg E. Energy efficient refining of black spruce TMP by using acid hydrogen peroxide: Part 1. A pilot plant study. Nordic Pulp & Paper Research Journal. 2009;24(3):255-65. | Irrelevant |
| Wan K, Jing Q, Zhao JZ. Evaluation of oral midazolam as conscious sedation for pediatric patients in oral restoration. Chinese medical sciences journal = Chung-kuo i hs¸eh k'o hs¸eh tsa chih / Chinese Academy of Medical Sciences. 2006;21(3):163-6. | Anesthesia |
| Wang H, Liu XY, Li L, Chen WM, Wang HY. [Application of adjustable oblique position for microendoscopic discectomy]. [Chinese]. Chung-Hua Wai Ko Tsa Chih [Chinese Journal of Surgery]. 2005;43(16):1080-3. | Irrelevant |
| Wang MY, Liu H, Li SL, Qin M. [Effects of mineral trioxide aggregate and calcium hydroxide on the proliferation and differentiation capacity of pulp cells of primary teeth]. [Chinese]. Chung-Hua Kou Chiang i Hsueh Tsa Chih Chinese Journal of Stomatology. 2008;43(9):524-7. | In vitro study |
| Wang XL, Yang PS, Yu Y, Sun SZ. Clinical evaluation of pulpotomy in the treatment of deciduous teeth with deep dentine caries. Shanghai kou qiang yi xue = Shanghai journal of stomatology. 2001;10(3):199-200, 42. | No RCT |
| Washington JT, Schneiderman E, Spears R, Fernandez CR, He JN, Opperman LA. Biocompatibility and Osteogenic Potential of New Generation Endodontic Materials Established by Using Primary Osteoblasts. Journal of Endodontics. 2011;37(8):1166-70. | In vitro study |
| Watamoto T, Egusa H, Mizumori T, Yashiro K, Takada K, Yatani H. Restoration of occlusal and proximal contacts by a single molar crown improves the smoothness of the masticatory movement. Journal of Dentistry. 2008;36(12):984-92. | Restorative dentistry |
| Waterhouse PJ, Nunn JH, Whitworth JM, Soames JV. Primary molar pulp therapy--histological evaluation of failure. International Journal of Paediatric Dentistry. 2000;10(4):313-21. | In vitro study |
| Watts AK, Thikkurissy S, Smiley M, McTigue DJ, Smith T. Local anesthesia affects physiologic parameters and reduces anesthesiologist intervention in children undergoing general anesthesia for dental rehabilitation. Pediatric Dentistry. 2009;31(5):414-9. | Anaesthesia |
| Weiger R, Rosendahl R, Lost C. Influence of calcium hydroxide intracanal dressings on the prognosis of teeth with endodontically induced periapical lesions. International Endodontic Journal. 2000;33(3):219-26. | Permanent teeth |
| Welbury RR, Shaw AJ, Murray JJ, Gordon PH, McCabe JF. Clinical evaluation of paired compomer and glass ionomer restorations in primary molars: final results after 42 months. British dental journal. 2000;189(2):93-7. | Restorative dentistry |
| Whitworth JM, Myers PM, Smith J, Walls AW, McCabe JF. Endodontic complications after plastic restorations in general practice. International Endodontic Journal. 2005;38(6):409-16. | Biomaterials not compared |
| Wigen TI, Agnalt R, Jacobsen I. Intrusive luxation of permanent incisors in Norwegians aged 6-17 years: a retrospective study of treatment and outcome. Dental Traumatology. 2008;24(6):612-8. | Permanent incisors |
| Wilcox LR, Diaz-Arnold A. Coronal microleakage of permanent lingual access restorations in endodontically treated anterior teeth. Journal of Endodontics. 1989;15(12):584-7. | In vitro study |
| Williams CE, Reid JS, Sharkey SW, Saunders WP. In-vitro measurement of apically extruded irrigant in primary molars. International Endodontic Journal. 1995;28(4):221-5. | In vitro study |
| Wilson TG, Primosch RE, Melamed B, Courts FJ. Clinical effectiveness of 1 and 2% lidocaine in young pediatric dental patients. Pediatric Dentistry. 1990;12(6):353-9. | Anesthesia |
| Winter GB. The root treatment of infected permanent incisors in children. British Dental Journal. 1966;120(1):11-3. | Permanent incisors |
| Wochna-Sobanska M. [Experimental studies on the bactericidal activity of various agents used in the treatment of dental pulp diseases in deciduous teeth]. [Polish]. Czasopismo Stomatologiczne. 1987;40(1):7-12. | Experimental study |
| Wochna-Sobanska M. [Treatment of pulp diseases in milk teeth with chlorocamphomenthol]. [Polish]. Czasopismo Stomatologiczne. 1990;43(7):401-3. | No RCT |
| Wright KJ, Derkson GD, Riding KH. Tissue-space emphysema, tissue necrosis, and infection following use of compressed air during pulp therapy: case report. Pediatric Dentistry. 1991;13(2):110-3. | Case report |
| Wu MK, Wesselink PR. A primary observation on the preparation and obturation of oval canals. International Endodontic Journal. 2001;34(2):137-41. | Permanent teeth |
| Wu SJ, Julliard K. Children's preference of benzocaine gel versus the lidocaine patch. Pediatric Dentistry. 2003;25(4):401-5. | Anesthesia |
| Wu YH, Hutton JE, Marshall GW. In vitro enamel demineralization and the marginal gap of simulated cast restorations with three different cements. Journal of prosthodontics : official journal of the American College of Prosthodontists. 1997;6(2):96-103. | In vitro study |
| Wu YN. [Clinical evaluation of the ultrasonic root canal instrumentation]. [Chinese]. Chung-Hua Kou Chiang i Hsueh Tsa Chih Chinese Journal of Stomatology. 1993;28(1):20-2. | Permanent teeth |
| Xie XL, Fang CY, Liu H. [Garlicin and formocresol in the treatment of chronic periapical periodontitis]. [Chinese]. Zhong Nan da Xue Xue Bao. 2004;Yi Xue Ban = Journal of Central South University. Medical Sciences. 29(2):221-3. | Permanent teeth |
| Xu X, Zhao JJ, Han JL. Self-solidifying hydroxyapatite/norvancomycin composite for pulp capping. Journal of Clinical Rehabilitative Tissue Engineering Research. 2008;12(45):8973-6. | Permanent teeth |
| Yaksi A, Ozgonenel L, Ozgonenel B. The efficiency of gabapentin therapy in patients with lumbar spinal stenosis. Spine. 2007;32(9):939-42. | Irrelevant |
| Yakushiji M, Sasamoto K, Imanishi T, Machida Y, Sekine N. [Clinical study of vital pulpotomy with Calvital on deciduous teeth]. [Japanese]. Shikwa Gakuho. 1969;69(2):271-5. | No RCT |
| Yamamoto K, Nagashima H, Yamachika S, Hoshiba D, Yamaguchi K, Yamada H, et al. The application of a night guard for sleep-related xerostomia. Oral Surgery, Oral Medicine, Oral Pathology, Oral Radiology, and Endodontics. 2008;106(3):e11-4. | Xerostomia |
| Yamashita JC, Tanomaru Filho M, Leonardo MR, Rossi MA, Silva LA. Scanning electron microscopic study of the cleaning ability of chlorhexidine as a root-canal irrigant. International Endodontic Journal. 2003;36(6):391-4. | In vitro study |
| Yang BR, Kalimo KO, Mattila LM, Kallio SE, Katajisto JK, Peltota OJ, et al. Effects of dietary supplementation with sea buckthorn (Hippophae rhamnoides) seed and pulp oils on atopic dermatitis. Journal of Nutritional Biochemistry. 1999;10(11):622-30. | Irrelevant |
| Yang TY, Zheng X. [Comparison of autosolidifying calcium phosphates cement and calcium hydroxide in repairs of deciduous molars with perforation in pulp chamber floor]. Journal of Clinical Rehabilitative Tissue Engineering Research. 2008;12(23):4534-6. | No pulp treatment |
| Yassen GH. Evaluation of mandibular infiltration versus mandibular block anaesthesia in treating primary canines in children. International Journal of Paediatric Dentistry. 2010;20(1):43-9. | Anesthesia |
| Yee R. An ART field study in western Nepal. International dental journal. 2001;51(2):103-8. | Restorative dentistry |
| Yeung SY, Lan WH, Huang CS, Lin CP, Chan CP, Chang MC, et al. Scavenging property of three cresol isomers against H2O2, hypochlorite, superoxide and hydroxyl radicals. Food and Chemical Toxicology. 2002;40(10):1403-13. | Irrelevant |
| Yilmaz S, Efeoglu E, Kilic AR. Alveolar ridge reconstruction and/or preservation using root form bioglass cones. Journal of Clinical Periodontology. 1998;25(10):832-9. | Periodontology |
| Yilmaz Y, Belduz N, Eyuboglu O. A two-year evaluation of four different fissure sealants. European archives of paediatric dentistry : official journal of the European Academy of Paediatric Dentistry. 2010;11(2):88-92. | Restorative dentistry |
| Yilmaz Y, Eyuboglu O, Keles S. Comparison of the efficacy of articaine and prilocaine local anaesthesia for pulpotomy of maxillary and mandibular primary molars. European Journal of Paediatric Dentistry. 2011;12(2):117-22. | Anesthesia |
| Yilmaz Y, Guler C, Sahin H, Eyuboglu O. Evaluation of tooth-fragment reattachment: a clinical and laboratory study. Dental Traumatology. 2010;26(4):308-14. | Traumatology |
| Yoke PC, Tin GB, Kim MJ, Rajaseharan A, Ahmed S, Thongprasom K, et al. A randomized controlled trial to compare steroid with cyclosporine for the topical treatment of oral lichen planus. Oral Surgery, Oral Medicine, Oral Pathology, Oral Radiology, and Endodontics. 2006;102(1):47-55. | Irrelevant |
| Yoldas O, Topuz A, Isci AS, Oztunc H. Postoperative pain after endodontic retreatment: single- versus two-visit treatment. Oral Surgery Oral Medicine Oral Pathology Oral Radiology & Endodontics. 2004;98(4):483-7. | Permanent teeth |
| Yoon RK, Chussid S, Davis MJ, Bruckman KC. Preferred treatment methods for primary tooth vital pulpotomies. A survey. New York State Dental Journal. 2008;74(2):47-9. | Survey |
| Yuasa H, Kurita K, Treatment Group on Temporomandibular D. Randomized clinical trial of primary treatment for temporomandibular joint disk displacement without reduction and without osseous changes: a combination of NSAIDs and mouth-opening exercise versus no treatment. Oral Surgery, Oral Medicine, Oral Pathology, Oral Radiology, and Endodontics. 2001;91(6):671-5. | Irrelevant |
| Zamany A, Safavi K, Spangberg LS. The effect of chlorhexidine as an endodontic disinfectant. Oral Surgery Oral Medicine Oral Pathology Oral Radiology & Endodontics. 2003;96(5):578-81. | Biomaterials not compared |
| Zarzar PA, Rosenblatt A, Takahashi CS, Takeuchi PL, Costa Junior LA. Formocresol mutagenicity following primary tooth pulp therapy: an in vivo study. Journal of Dentistry. 2003;31(7):479-85. | Case-control study |
| Zehnder M, Guggenheim B. The mysterious appearance of enterococci in filled root canals. International Endodontic Journal. 2009;42(4):277-87. | Bacteriology |
| Zehnder M, Luder HU, Sch‰tzle M, Kerosuo E, Waltimo T. A comparative study on the disinfection potentials of bioactive glass S53P4 and calcium hydroxide in contra-lateral human premolars ex vivo. International endodontic journal. 2006;39(12):952-8. | Premolars |
| Zerella JA, Fouad AF, SpÂngberg LS. Effectiveness of a calcium hydroxide and chlorhexidine digluconate mixture as disinfectant during retreatment of failed endodontic cases. Oral Surgery, Oral Medicine, Oral Pathology, Oral Radiology, and Endodontics. 2005;100(6):756-61. | Permanent teeth |
| Zhang J, Li JZ, Zhao ZG, Zhang LP. [Comparison of different materials during apexification of young permanent teeth]. Journal of Clinical Rehabilitative Tissue Engineering Research. 2009;13(16):3141-4. | Permanent teeth |
| Zhang Q, Mulder J, Truin GJ, van Palenstein Helderman WH. Effect of 40% chlorhexidine varnish on mutans streptococci counts in pits and fissures of permanent first molars. Journal of dentistry. 2007;35(7):588-92. | Permanent teeth |
| Zhang SH, Sun B, Wang W, Zhu MF, Chen JH. Adsorption of Cd2+ and Cu2+ by oxidized cellulose from TEMPO-mediated selective oxidation of alkaline natural cellulose pulp. Journal of Macromolecular Science Part a-Pure and Applied Chemistry. 2006;43(11):1895-906. | Irrelevant |
| Zheng Y, Liu Y, Zhang CM, Zhang HY, Li WH, Shi S, et al. Stem Cells from Deciduous Tooth Repair Mandibular Defect in Swine. Journal of Dental Research. 2009;88(3):249-54. | Animal study |
| Zimmer S, ÷zt¸rk M, Barthel CR, Bizhang M, Jordan RA. Cleaning efficacy and soft tissue trauma after use of manual toothbrushes with different bristle stiffness. Journal of Periodontology. 2011;82(2):267-71. | Periodontology |
| Ziskind D, Adell I, Teperovich E, Peretz B. The effect of an intermediate layer of flowable composite resin on microleakage in packable composite restorations. International Journal of Paediatric Dentistry. 2005;15(5):349-54. | In vitro study |
| Zmener O, Banegas G, Pameijer CH. Coronal microleakage of three temporary restorative materials: An in vitro study. Journal of Endodontics. 2004;30(8):582-4. | In vitro study |
| Zoremchhingi, Joseph T, Varma B, Mungara J. A study of root canal morphology of human primary molars using computerised tomography: an in vitro study. Journal of the Indian Society of Pedodontics & Preventive Dentistry. 2005;23(1):7-12. | In vitro study |
| Zulfikaroglu BT, Atac AS, Cehreli ZC. Clinical performance of Class II adhesive restorations in pulpectomized primary molars: 12-month results. Journal of Dentistry for Children (Chicago, Ill. 2008). 75(1):33-43. | Restorative dentistry |

*“Irrelevant”: other medical fields (urology, orofacial surgery, clinical orthopedics, phlebology, herpes, cancer, etc.) and non-medical fields (paper making, etc.)*
